# Immunomodulators and immunosuppressants for progressive multiple sclerosis: a network meta‐analysis

**DOI:** 10.1002/14651858.CD015443.pub2

**Published:** 2024-09-10

**Authors:** Ben Ridley, Silvia M Minozzi, Marien Gonzalez-Lorenzo, Cinzia Del Giovane, Thomas Piggott, Graziella Filippini, Guy Peryer, Matteo Foschi, Irene Tramacere, Elisa Baldin, Francesco Nonino

**Affiliations:** IRCCS Istituto delle Scienze Neurologiche di BolognaBolognaItaly; Department of EpidemiologyLazio Regional Health ServiceRomeItaly; Laboratorio di Metodologia delle revisioni sistematiche e produzione di Linee GuidaIstituto di Ricerche Farmacologiche Mario Negri IRCCSMilanItaly; Institute of Primary Health Care (BIHAM)BernSwitzerland; Department of Medical and Surgical SciencesUniversity of Modena and Reggio EmiliaModenaItaly; Department of Health Research Methods, Evidence, and ImpactMcMaster UniversityHamilton, OntarioCanada; Department of Family MedicineQueens UniversityKingston, OntarioCanada; Scientific Director’s OfficeFondazione IRCCS, Istituto Neurologico Carlo BestaMilanItaly; School of Health SciencesUniversity of East AngliaNorwichUK; Department of Neuroscience, Multiple Sclerosis Center - Neurology Unit, S.Maria delle Croci HospitalAUSL RomagnaRavennaItaly; Department of Biotechnological and Applied Clinical SciencesUniversity of L'AquilaL'AquilaItaly; Department of Research and Clinical Development, Scientific DirectorateFondazione IRCCS Istituto Neurologico Carlo BestaMilanItaly

**Keywords:** Humans, Immunomodulating Agents, Immunomodulating Agents/administration & dosage, Immunomodulating Agents/adverse effects, Immunosuppressive Agents, Immunosuppressive Agents/administration & dosage, Immunosuppressive Agents/adverse effects, Multiple Sclerosis, Chronic Progressive, Multiple Sclerosis, Chronic Progressive/diagnosis, Multiple Sclerosis, Chronic Progressive/drug therapy, Randomized Controlled Trials as Topic, Recurrence, Rituximab, Rituximab/administration & dosage, Rituximab/adverse effects

## Abstract

**Background:**

In recent years a broader range of immunomodulatory and immunosuppressive treatment options have emerged for people with progressive forms of multiple sclerosis (PMS). While consensus supports these options as reducing relapses, their relative benefit and safety profiles remain unclear due to a lack of direct comparison trials.

**Objectives:**

To compare through network meta‐analysis the efficacy and safety of alemtuzumab, azathioprine, cladribine, cyclophosphamide, daclizumab, dimethylfumarate, diroximel fumarate, fingolimod, fludarabine, glatiramer acetate, immunoglobulins, interferon beta 1‐a and beta 1‐b, interferon beta‐1b (Betaferon), interferon beta‐1a (Avonex, Rebif), laquinimod, leflunomide, methotrexate, minocycline, mitoxantrone, mycophenolate mofetil, natalizumab, ocrelizumab, ofatumumab, ozanimod, pegylated interferon beta‐1a, ponesimod, rituximab, siponimod, corticosteroids, and teriflunomide for PMS.

**Search methods:**

We searched CENTRAL, MEDLINE, and Embase up to August 2022, as well as ClinicalTrials.gov and the WHO ICTRP.

**Selection criteria:**

Randomised controlled trials (RCTs) that studied one or more treatments as monotherapy, compared to placebo or to another active agent, for use in adults with PMS.

**Data collection and analysis:**

Two review authors independently selected studies and extracted data. We performed data synthesis by pair‐wise and network meta‐analysis. We assessed the certainty of the body of evidence according to GRADE.

**Main results:**

We included 23 studies involving a total of 10,167 participants.

The most frequent (39% of studies) reason for a rating of high risk of bias was sponsor role in study authorship and data management and analysis. Other concerns were performance, attrition, and selective reporting bias, with 8.7% of studies at high risk of bias for all three of these domains.

The common comparator for network analysis was placebo.

**Relapses over 12 months:** assessed in one study (318 participants). None of the treatments assessed showed moderate or high certainty evidence compared to placebo.

**Relapses over 24 months:** assessed in six studies (1622 participants). The number of people with clinical relapses is probably trivially reduced with rituximab (risk ratio (RR) 0.60, 95% confidence interval (CI) 0.19 to 1.95; moderate certainty evidence). None of the remaining treatments assessed showed moderate or high certainty evidence compared to placebo.

**Relapses over 36 months:** assessed in four studies (2095 participants). The number of people with clinical relapses is probably trivially reduced with interferon beta‐1b (RR 0.82, 95% CI 0.73 to 0.93; moderate certainty evidence). None of the remaining treatments assessed showed moderate or high certainty evidence compared to placebo.

**Disability worsening over 24 months:** assessed in 11 studies (5284 participants). None of the treatments assessed showed moderate or high certainty evidence compared to placebo.

**Disability worsening over 36 months:** assessed in five studies (2827 participants). None of the treatments assessed showed moderate or high certainty evidence compared to placebo.

**Serious adverse events:** assessed in 15 studies (8019 participants). None of the treatments assessed showed moderate or high certainty evidence compared to placebo.

**Discontinuation due to adverse events:** assessed in 21 studies (9981 participants). The number of people who discontinued treatment due to adverse events is trivially increased with interferon beta‐1a (odds ratio (OR) 2.93, 95% CI 1.64 to 5.26; high certainty evidence). The number of people who discontinued treatment due to adverse events is probably trivially increased with rituximab (OR 4.00, 95% CI 0.84 to 19.12; moderate certainty evidence); interferon beta‐1b (OR 2.98, 95% CI 1.92 to 4.61; moderate certainty evidence); immunoglobulins (OR 1.95, 95% CI 0.99 to 3.84; moderate certainty evidence); glatiramer acetate (OR 3.98, 95% CI 1.48 to 10.72; moderate certainty evidence); natalizumab (OR 1.02, 95% CI 0.55 to 1.90; moderate certainty evidence); siponimod (OR 1.53, 95% CI 0.98 to 2.38; moderate certainty evidence); fingolimod (OR 2.29, 95% CI 1.46 to 3.60; moderate certainty evidence), and ocrelizumab (OR 1.24, 95% CI 0.54 to 2.86; moderate certainty evidence). None of the remaining treatments assessed showed moderate or high certainty evidence compared to placebo.

**Authors' conclusions:**

The number of people with PMS with relapses is probably slightly reduced with rituximab at two years, and interferon beta‐1b at three years, compared to placebo. Both drugs are also probably associated with a slightly higher proportion of withdrawals due to adverse events, as are immunoglobulins, glatiramer acetate, natalizumab, fingolimod, siponimod, and ocrelizumab; we have high confidence that this is the case with interferon beta‐1a.

We found only low or very low certainty evidence relating to disability progression for the included disease‐modifying treatments compared to placebo, largely due to imprecision. We are also uncertain about the effect of interventions on serious adverse events, also because of imprecision.

These findings are due in part to the short follow‐up of the included RCTs, which lacked detection of less common severe adverse events. Moreover, the funding source of many included studies may have introduced bias into the results.

Future research on PMS should include head‐to‐head rather than placebo‐controlled trials, with a longer follow‐up of at least three years. Given the relative rarity of PMS, controlled, non‐randomised studies on large samples may usefully integrate data from pivotal RCTs. Outcomes valuable and meaningful to people with PMS should be consistently adopted and measured to permit the evaluation of relative effectiveness among treatments.

## Plain language summary

**What are the benefits and risks of different treatments that could delay or slow the progression of progressive multiple sclerosis?**

**Key messages**

• Overall, we are very uncertain about the effects of treatments on relapses and slowing the worsening of disability. We did find evidence that rituximab after two years and interferon beta‐1b after three years of treatment probably slightly reduce the number of people who experience relapses.

• The number of people who stop taking a drug because of harmful events is slightly higher with interferon beta‐1a, and probably slightly higher with interferon beta‐1b, rituximab, immunoglobulins, glatiramer acetate, natalizumab, fingolimod, siponimod, and ocrelizumab.

• Longer studies that make comparisons between treatments are needed to assess the benefits and harms of drugs acting on the immune system over time for people with progressive multiple sclerosis. Future studies should also consider other effects that are important to people with progressive multiple sclerosis, such as quality of life and ability to think, learn, remember, use judgement, and make decisions.

**Background**

Multiple sclerosis is caused by inflammation of the brain and spine due to an impairment of the immune system, resulting in damage that gradually limits activities of daily living. People with multiple sclerosis typically experience tiredness, pain, cramps in their muscles, and reduction or loss of sensitivity and strength in parts of their body. The appearance of symptoms is called 'relapse', and is usually followed by gradual recovery ('remission'), in what is known as 'relapsing‐remitting' multiple sclerosis. When recovery doesn't happen or is incomplete between relapses, it is known as 'progressive' multiple sclerosis.

Over the years, in most people with relapsing‐remitting multiple sclerosis, worsening of disability will become continuous, without recovery. This is known as 'secondary‐progressive' multiple sclerosis. In about 15 out of 100 cases, multiple sclerosis shows a progressive course from the onset, without relapse and recovery. This is called 'primary progressive' multiple sclerosis.

Multiple sclerosis affects males and females in equal proportion, with onset occurring most often between the ages of 30 and 50 years.

**How is multiple sclerosis treated?**

Although there is no cure for multiple sclerosis, so‐called disease‐modifying drugs can reduce the frequency of relapses and slow or delay the progression of disability. Fewer treatments are available for progressive multiple sclerosis than for other forms of the disease, but more have been approved in recent years.

**What did we want to find out?**

We wanted to find out:

• which treatments produce the most benefit, in terms of the number of people with a reduction of relapses or disability worsening; and

• if any drug is better tolerated than any other drug or causes fewer unwanted effects.

**What did we do?**

We searched for studies that compared different disease‐modifying treatments with each other or to placebo ('dummy' or sham treatment). We compared and summarised the results and rated our confidence in the evidence based on factors such as study methods and sizes.

**What did we find?**

We found 23 studies involving a total of 10,167 people with progressive multiple sclerosis who were treated with a disease‐modifying drug or placebo for at least one year. The number of participants enrolled in the studies ranged from 27 to 1651. Most studies lasted 12 or 24 months, with only four studies lasting more than 24 months. Most of the included studies were conducted by drug companies to obtain regulatory approval to sell the drug. Twenty studies compared disease‐modifying treatments to placebo, and three studies compared different disease‐modifying treatments to each other.

We are confident that slightly more people stop taking interferon beta‐1a because of unwanted effects when compared to placebo.

We are moderately confident that rituximab after two years and interferon beta‐1b after three years of treatment slightly reduce the number of people with relapses, and slightly more people stop taking interferon beta‐1b, rituximab, immunoglobulins, glatiramer acetate, natalizumab, fingolimod, siponimod, and ocrelizumab because of unwanted effects, when compared to placebo.

We are very uncertain about the effect of the other treatments studied on number of people with relapses, number of people with a worsening of disability, number of people who stop taking the drug because of unwanted effects, and number of people with serious unwanted effects.

**What are the limitations of the evidence?**

Our confidence in the effects of disease‐modifying drugs is very limited because the evidence was based on relatively low numbers of people experiencing events like relapses and worsening of disability, and because we were concerned that the interests of drug companies may have influenced the reporting of results.

**How up‐to‐date is this evidence**?

The evidence is current to 8 August 2022.

## Summary of findings

**Summary of findings 1:** Relapse at 12 months

**Patient or population:** People with PMS **Interventions:** Immunoglobulins **Comparator (reference):** Placebo **Outcome:** Relapses at 12 months **Setting(s):** Outpatient					
**Total studies; total participants**	Relative effect* (95% CI)	Anticipated absolute effect**(95% CI)			**Certainty of the evidence**
		**With placebo**	**With intervention**	**Difference**	
Immunoglobulins (direct evidence; 1 RCT; 318 participants)	**RR 1.04** (0.76 to 1.41)	**333 per 1000**	**347 per 1000**	**13 more per 1000** (from 80 fewer to 137 more)	⊕◯◯◯ Very low due to imprecision^1^
Placebo	Reference comparator	Not estimable	Not estimable	Not estimable	Reference comparator
*Network meta‐analysis estimates are reported as risk ratio (RR). CI: confidence interval. **Anticipated absolute effect compares 2 risks by calculating the difference between the risk of the intervention group and the risk of the control group. **PMS:** progressive multiple sclerosis; **RCT:** randomised controlled trial					
**GRADE Working Group grades of evidence** **High certainty:** We are very confident that the true effect lies close to that of the estimate of the effect. **Moderate certainty:** We are moderately confident in the effect estimate: the true effect is likely to be close to the estimate of the effect, but there is a possibility that it is substantially different. **Low certainty:** Our confidence in the effect estimate is limited: the true effect may be substantially different from the estimate of the effect. **Very low certainty:** We have very little confidence in the effect estimate: the true effect is likely to be substantially different from the estimate of effect.					

^1^Absolute observed point estimate falls in the trivial negative effect, 95% CI ranges from small positive effect to moderate negative effect; downgraded three levels.

**Summary of findings 2:** Relapses at 24 months

**Patient or population:** People with PMS **Interventions:** Rituximab, methotrexate, immunoglobulins, interferon beta‐1a (Avonex, Rebif) **Comparator (reference):** Placebo **Outcome:** Relapse at 24 months **Setting(s):** Outpatient					
**Total studies; total participants**	Relative effect* (95% CI)	Anticipated absolute effect**(95% CI)			**Certainty of the evidence**
		**With placebo**	**With intervention**	**Difference**	
Rituximab (direct evidence; 1 RCT; 439 participants)	**RR 0.60** (0.19 to 1.95)	**34 per 1000**	**20 per 1000**	**14 fewer per 1000** (from 28 fewer to 32 more)	⊕⊕⊕◯ Moderate due to imprecision^1^
Methotrexate (direct evidence; 1 RCT; 60 participants)	**RR 1.12** (0.38 to 3.28)	**172 per 1000**	**193 per 1000**	**21 more per 1000** (from 107 fewer to 393 more)	⊕◯◯◯ Very low due to imprecision^2^
Immunoglobulins (direct evidence; 2 RCTs; 549 participants)	**RR 0.96** (0.79 to 1.16)	**431 per 1000**	**413 per 1000**	**17 fewer per 1000** (from 90 fewer to 69 more)	⊕◯◯◯ Very low due to imprecision^3^
Interferon beta‐1a (Avonex, Rebif) (direct evidence; 1 RCT; 436 participants)	**RR 0.72** (0.54 to 0.95)	**365 per 1000**	**263 per 1000**	**102 fewer per 1000** (from 168 fewer to 18 fewer)	⊕◯◯◯ Very low due to imprecision^4^
Placebo	Reference Comparator	Not estimable	Not estimable	Not estimable	Reference Comparator
*Network meta‐analysis estimates are reported as risk ratio (RR). CI: confidence interval. **Anticipated absolute effect compares 2 risks by calculating the difference between the risk of the intervention group and the risk of the control group. **PMS:** progressive multiple sclerosis; **RCT:** randomised controlled trial					
**GRADE Working Group grades of evidence** **High certainty:** We are very confident that the true effect lies close to that of the estimate of the effect. **Moderate certainty:** We are moderately confident in the effect estimate: the true effect is likely to be close to the estimate of the effect, but there is a possibility that it is substantially different. **Low certainty:** Our confidence in the effect estimate is limited: the true effect may be substantially different from the estimate of the effect. **Very low certainty:** We have very little confidence in the effect estimate: the true effect is likely to be substantially different from the estimate of effect.					

^1^Absolute observed point estimate falls in the trivial positive effect, 95% CI ranges from trivial positive effect to trivial negative effect; downgraded one level.  ^2^Absolute observed point estimate falls in the trivial negative effect, 95% CI ranges from moderate positive effect to large negative effect; downgraded three levels. ^3^Absolute observed point estimate falls in the trivial positive effect, 95% CI ranges from moderate positive effect to small negative effect; downgraded three levels. ^4^Absolute observed point estimate falls in the moderate positive effect, 95% CI ranges from large positive effect to trivial positive effect; downgraded three levels.

**Summary of findings 3:** Disability at 24 months

**Patient or population:** People with PMS **Interventions:** Glatiramer acetate, immunoglobulins, interferon beta‐1b (Betaferon), interferon beta‐1a (Avonex, Rebif), methotrexate, natalizumab, siponimod, rituximab **Comparator (reference):** Placebo **Outcome:** Disability at 24 months **Setting(s):** Outpatient					
**Total studies; total participants**	Relative effect* (95% CI)	Anticipated absolute effect**(95% CI)			**Certainty of the evidence**
		**With placebo**	**With intervention**	**Difference**	
Glatiramer acetate (direct evidence; 2 RCTs; 1049 participants)	**RR 0.84** (0.59 to 1.20)	**423 per 1000**	**355 per 1000**	**68 fewer per 1000** (from 174 fewer to 85 more)	**⊕◯◯◯** Very low due to imprecision^1^
Immunoglobulins (direct evidence; 2 RCTs; 549 participants)	**RR 0.92** (0.68 to 1.25)	**518 per 1000**	**477 per 1000**	**41 fewer per 1000** (from 166 fewer to 130 more)	⊕◯◯◯ Very low due to imprecision^2^
Interferon beta‐1b (Betaferon) (direct evidence; 1 RCT; 73 participants)	**RR 0.69** (0.29 to 1.60)	**324 per 1000**	**224 per 1000**	**101 fewer per 1000** (from 230 fewer to 195 more)	⊕◯◯◯ Very low due to imprecision^3^
Interferon beta‐1a (Avonex, Rebif) (direct evidence; 1 RCT; 436 participants)	**RR 0.85** (0.54 to 1.33)	**338 per 1000**	**287 per 1000**	**51 fewer per 1000** (from 155 fewer to 112 more)	⊕◯◯◯ Very low due to imprecision^4^
Methotrexate (direct evidence; 1 RCT; 60 participants)	**RR 0.69** (0.34 to 1.37)	**517 per 1000**	**357 per 1000**	**160 fewer per 1000** (from 341 fewer to 191 more)	⊕◯◯◯ Very low due to imprecision^5^
Natalizumab (direct evidence; 1 RCT; 889 participants)	**RR 0.83** (0.55 to 1.27)	**294 per 1000**	**244 per 1000**	**50 fewer per 1000** (from 132 fewer to 79 more)	⊕◯◯◯ Very low due to imprecision^6^
Siponimod (direct evidence; 1 RCT; 1651 participants)	**RR 0.77** (0.52 to 1.16)	**255 per 1000**	**196 per 1000**	**59 fewer per 1000** (from 122 fewer to 41 more)	⊕◯◯◯ Very low due to imprecision^7^
Rituximab (direct evidence; 1 RCT; 439 participants)	**RR 0.78** (0.50 to 1.21)	**388 per 1000**	**302 per 1000**	**85 fewer per 1000** (from 194 fewer to 81 more)	**⊕◯◯◯** Very low due to imprecision^8^
Placebo	Reference comparator	Not estimable	Not estimable	Not estimable	Reference comparator
*Network meta‐analysis estimates are reported as risk ratio (RR). **Anticipated absolute effect compares 2 risks by calculating the difference between the risk of the intervention group and the risk of the control group. **CI:** confidence interval; **PMS:** progressive multiple sclerosis; **RCT:** randomised controlled trial					
**GRADE Working Group grades of evidence** **High certainty:** We are very confident that the true effect lies close to that of the estimate of the effect. **Moderate certainty:** We are moderately confident in the effect estimate: the true effect is likely to be close to the estimate of the effect, but there is a possibility that it is substantially different. **Low certainty:** Our confidence in the effect estimate is limited: the true effect may be substantially different from the estimate of the effect. **Very low certainty:** We have very little confidence in the effect estimate: the true effect is likely to be substantially different from the estimate of effect.					

^1^Absolute observed point estimate falls in the moderate positive effect, 95% CI ranges from large positive effect to moderate negative effect; downgraded three levels. ^2^Absolute observed point estimate falls in the small positive effect, 95% CI ranges from large positive effect to large negative effect; downgraded three levels. ^3^Absolute observed point estimate falls in the moderate positive effect, 95% CI ranges from large positive effect to large negative effect; downgraded three levels. ^4^Absolute observed point estimate falls in the small positive effect, 95% CI ranges from large positive effect to large negative effect; downgraded three levels. ^5^Absolute observed point estimate falls in the large positive effect, 95% CI ranges from large positive effect to large negative effect; downgraded three levels. ^6^Absolute observed point estimate falls in the small positive effect, 95% CI ranges from large positive effect to moderate negative effect; downgraded three levels. ^7^Absolute observed point estimate falls in the moderate positive effect, 95% CI ranges from large positive effect to small negative effect; downgraded three levels. ^8^Absolute observed point estimate falls in the moderate positive effect, 95% CI ranges from large positive effect to moderate negative effect; downgraded three levels.

**Summary of findings 4:** Relapses at 36 months

**Patient or population:** People with PMS **Interventions:** Azathioprine, interferon beta‐1a (Avonex, Rebif), interferon beta‐1b (Betaferon) **Comparator (reference):** Placebo **Outcome:** Relapse at 36 months **Setting(s):** Outpatient					
**Total studies; total participants**	Relative effect* (95% CI)	Anticipated absolute effect**(95% CI)			**Certainty of the evidence**
		**With placebo**	**With intervention**	**Difference**	
Azathioprine (direct evidence; 1 RCT; 67 participants)	**RR 0.54** (0.30 to 0.99)	**559 per 1000**	**302 per 1000**	**257 fewer per 1000** (from 391 fewer to 6 fewer)	⊕◯◯◯ Very low due to imprecision^1^
Interferon beta‐1a (Avonex, Rebif) (direct evidence; 1 RCT; 371 participants)	**RR 1.03** (0.79 to 1.34)	**372 per 1000**	**383 per 1000**	**11 more per 1000** (from 115 fewer to 126 more)	⊕◯◯◯ Very low due to imprecision^2^
Interferon beta‐1b (Betaferon) (direct evidence; 2 RCTs; 1657 participants)	**RR 0.82** (0.73 to 0.93)	**159 per 1000**	**131 per 1000**	**29 fewer per 1000** (from 43 fewer to 11 fewer)	⊕⊕⊕◯ Moderate due to imprecision^3^
Placebo	Reference comparator	Not estimable	Not estimable	Not estimable	Reference comparator
*Network meta‐analysis estimates are reported as risk ratio (RR). CI: confidence interval. **Anticipated absolute effect compares 2 risks by calculating the difference between the risk of the intervention group and the risk of the control group. **PMS:** progressive multiple sclerosis; **RCT:** randomised controlled trial					
**GRADE Working Group grades of evidence** **High certainty:** We are very confident that the true effect lies close to that of the estimate of the effect. **Moderate certainty:** We are moderately confident in the effect estimate: the true effect is likely to be close to the estimate of the effect, but there is a possibility that it is substantially different. **Low certainty:** Our confidence in the effect estimate is limited: the true effect may be substantially different from the estimate of the effect. **Very low certainty:** We have very little confidence in the effect estimate: the true effect is likely to be substantially different from the estimate of effect.					

^1^Absolute observed point estimate falls in the large positive effect, 95% CI ranges from large positive effect to trivial positive effect; downgraded three levels. ^2^Absolute observed point estimate falls in the trivial negative effect, 95% CI ranges from moderate negative effect to moderate positive effect; downgraded three levels. ^3^Absolute observed point estimate falls in the trivial positive effect, 95% CI ranges from small positive effect to trivial positive effect; downgraded one level.

**Summary of findings 5:** Disability at 36 months

**Patient or population:** People with PMS **Interventions:** Interferon beta‐1b (Betaferon), interferon beta‐1a (Avonex, Rebif), azathioprine, ocrelizumab **Comparator (reference):** Placebo **Outcome:** Disability at 36 months **Setting(s):** Outpatient					
**Total studies; total participants**	Relative effect* (95% CI)	Anticipated absolute effect**(95% CI)			**Certainty of the evidence**
		**With placebo**	**With intervention**	**Difference**	
Interferon beta‐1b (Betaferon) (direct evidence; 2 RCTs; 1657 participants)	**RR 0.90** (0.68 to 1.18)	**425 per 1000**	**382 per 1000**	**42 fewer per 1000** (from 136 fewer to 76 more)	⊕◯◯◯ Very low due to imprecision^1^
Interferon beta‐1a (Avonex, Rebif) (direct evidence; 1 RCT; 371 participants)	**RR 1.10** (0.72 to 1.70)	**372 per 1000**	**409 per 1000**	**37 more per 1000** (from 104 fewer to 260 more)	⊕◯◯◯ Very low due to imprecision^2^
Azathioprine (direct evidence; 1 RCT; 67 participants)	**RR 0.63** (0.28 to 1.44)	**382 per 1000**	**241 per 1000**	**141 fewer per 1000** (from 275 fewer to 168 more)	⊕◯◯◯ Very low due to imprecision^3^
Ocrelizumab (direct evidence; 1 RCT; 732 participants)	**RR 0.83** (0.55 to 1.25)	**357 per 1000**	**296 per 1000**	**61 fewer per 1000** (from 160 fewer to 89 more)	⊕◯◯◯ Very low due to imprecision^4^
Placebo	Reference comparator	Not estimable	Not estimable	Not estimable	Reference comparator
*Network meta‐analysis estimates are reported as risk ratio (RR). CI: confidence interval. **Anticipated absolute effect compares 2 risks by calculating the difference between the risk of the intervention group and the risk of the control group. **PMS:** progressive multiple sclerosis; **RCT:** randomised controlled trial					
**GRADE Working Group grades of evidence** **High certainty:** We are very confident that the true effect lies close to that of the estimate of the effect. **Moderate certainty:** We are moderately confident in the effect estimate: the true effect is likely to be close to the estimate of the effect, but there is a possibility that it is substantially different. **Low certainty:** Our confidence in the effect estimate is limited: the true effect may be substantially different from the estimate of the effect. **Very low certainty:** We have very little confidence in the effect estimate: the true effect is likely to be substantially different from the estimate of effect.					

^1^Absolute observed point estimate falls in the small positive effect, 95% CI ranges from large positive effect to moderate negative effect; downgraded three levels. ^2^Absolute observed point estimate falls in the small negative effect, 95% CI ranges from large positive effect to large negative effect; downgraded three levels. ^3^Absolute observed point estimate falls in the large positive effect, 95% CI ranges from large positive effect to large negative effect; downgraded three levels. ^4^Absolute observed point estimate falls in the moderate positive effect, 95% CI ranges from large positive effect to moderate negative effect; downgraded three levels.

**Summary of findings 6:** Serious adverse events*

**Patient or population:** People with PMS **Interventions:** Rituximab, interferon beta‐1a (Avonex, Rebif), methotrexate, immunoglobulins, interferon beta‐1b (Betaferon), glatiramer acetate, natalizumab, siponimod, fingolimod, ocrelizumab, laquinimod **Comparator (reference):** Placebo **Outcome:** Serious adverse events **Setting(s):** Outpatient					
**Total studies; total participants**	Relative effect** (95% CI)	Anticipated absolute effect***(95% CI)			**Certainty of the evidence**
		**With placebo**	**With intervention**	**Difference**	
Rituximab (direct evidence; 2 RCTs; 466 participants)	**OR 1.05** (0.37 to 3.01)	**154 per 1000**	**160 per 1000**	**6 more per 1000** (from 91 fewer to 200 more)	⊕◯◯◯ Very low due to imprecision^1^
Interferon beta‐1a (Avonex, Rebif) (direct evidence; 1 RCT; 364 participants)	**OR 0.99** (0.51 to 1.92)	**275 per 1000**	**273 per 1000**	**2 fewer per 1000** (from 113 fewer to 146 more)	⊕◯◯◯ Very low due to imprecision^2^
Methotrexate (direct evidence; 1 RCT; 62 participants)	**OR 0.94** (0.02 to 50.12)	**17 per 1000**	**16 per 1000**	**1 fewer per 1000** (from 16 fewer to 443 more)	⊕◯◯◯ Very low due to imprecision^3^
Immunoglobulins (direct evidence; 2 RCTs; 549 participants)	**OR 7.13** (1.23 to 41.34)	**5 per 1000**	**38 per 1000**	**32 more per 1000** (from 1 more to 179 more)	⊕◯◯◯ Very low due to imprecision^4^
Interferon beta‐1b (Betaferon) (direct evidence; 1 RCT; 939 participants)	**OR 0.98** (0.56 to 1.72)	**279 per 1000**	**275 per 1000**	**4 fewer per 1000** (from 59 fewer to 61 more)	⊕⊕◯◯ Low due to imprecision^5^
Glatiramer acetate (direct evidence; 1 RCT; 943 participants)	**OR 1.50** (0.54 to 4.14)	**19 per 1000**	**28 per 1000**	**9 more per 1000** (from 9 fewer to 55 more)	⊕⊕◯◯ Low due to imprecision^6^
Natalizumab (direct evidence; 1 RCT; 888 participants)	**OR 0.90** (0.51 to 1.59)	**223 per 1000**	**205 per 1000**	**18 fewer per 1000** (from 95 fewer to 90 more)	⊕◯◯◯ Very low due to imprecision^7^
Siponimod (direct evidence; 1 RCT; 1646 participants)	**OR 1.22** (0.70 to 2.10)	**152 per 1000**	**179 per 1000**	**27 more per 1000** (from 41 fewer to 121 more)	⊕◯◯◯ Very low due to imprecision^8^
Fingolimod (direct evidence; 1 RCT; 823 participants)	**OR 1.05** (0.60 to 1.87)	**240 per 1000**	**249 per 1000**	**9 more per 1000** (from 81 fewer to 131 more)	⊕◯◯◯ Very low due to imprecision^9^
Ocrelizumab (direct evidence; 1 RCT; 725 participants)	**OR 0.90** (0.49 to 1.64)	**222 per 1000**	**204 per 1000**	**18 more per 1000** (from 99 fewer to 97 more)	⊕◯◯◯ Very low due to imprecision^10^
Laquinimod (direct evidence; 1 RCT; 373 participants)	**OR 1.32** (0.44 to 3.95)	**43 per 1000**	**56 per 1000**	**13 more per 1000** (from 24 fewer to 107 more)	⊕◯◯◯ Very low due to imprecision^11^
Placebo	Reference comparator	Not estimable	Not estimable	Not estimable	Reference comparator
*Network meta‐analysis estimates including only available comparisons vs placebo (common comparator) are reported. The only available study on methotrexate vs placebo, Goodkin 1995, reported zero events in both groups relative to serious adverse events. Network meta‐analysis was performed by means of STATA. In order to retain methotrexate in the network for indirect comparisons, a value of 0.5 events was imputed, giving an odds ratio (OR) value of 0.94 (95% CI 0.02 to 50.12). In Analysis 2.1 (pair‐wise meta‐analysis), the pair‐wise OR was calculated using RevMan, allowing only the value of zero events. Therefore, the forest plot reports zero events and the 'not estimable' warning. **Network meta‐analysis estimates are reported as risk ratio (RR). CI: confidence interval. ***Anticipated absolute effect compares 2 risks by calculating the difference between the risk of the intervention group and the risk of the control group. **PMS:** progressive multiple sclerosis; **RCT:** randomised controlled trial					
**GRADE Working Group grades of evidence** **High certainty:** We are very confident that the true effect lies close to that of the estimate of the effect. **Moderate certainty:** We are moderately confident in the effect estimate: the true effect is likely to be close to the estimate of the effect, but there is a possibility that it is substantially different. **Low certainty:** Our confidence in the effect estimate is limited: the true effect may be substantially different from the estimate of the effect. **Very low certainty:** We have very little confidence in the effect estimate: the true effect is likely to be substantially different from the estimate of effect.					

^1^Absolute observed point estimate falls in the trivial negative effect (below small effect threshold), 95% CI ranges from moderate positive effect to large negative effect; downgraded three levels.  ^2^Absolute observed point estimate falls in the trivial positive effect, 95% CI ranges from moderate positive effect to moderate negative effect; downgraded three levels. ^3^Absolute observed point estimate falls in the trivial positive effect, 95% CI ranges from trivial positive effect to large negative effect; downgraded three levels. ^4^Absolute observed point estimate falls in the trivial negative effect, 95% CI ranges from trivial negative effect to large negative effect; downgraded three levels. ^5^Absolute observed point estimate falls in the trivial positive effect, 95% CI ranges from large positive effect to small positive effect; downgraded two levels. ^6^Absolute observed point estimate falls in the trivial negative effect, 95% CI ranges from trivial positive effect to small negative effect; downgraded two levels. ^7^Absolute observed point estimate falls in the trivial positive effect, 95% CI ranges from moderate positive effect to moderate negative effect; downgraded three levels. ^8^Absolute observed point estimate falls in the trivial negative effect, 95% CI ranges from small positive effect to moderate negative effect; downgraded three levels. ^9^Absolute observed point estimate falls in the trivial negative effect, 95% CI ranges from moderate positive effect to moderate negative effect; downgraded three levels. ^10^Absolute observed point estimate falls in the trivial negative effect, 95% CI ranges from moderate positive effect to moderate negative effect; downgraded three levels. ^11^Absolute observed point estimate falls in the trivial negative effect, 95% CI ranges from trivial positive effect to moderate negative effect; downgraded three levels.

**Summary of findings 7:** Treatment discontinuation due to adverse events

**Patient or population:** People with PMS **Interventions:** Azathioprine, rituximab, interferon beta‐1a (Avonex, Rebif), interferon beta‐1b, immunoglobulins, glatiramer acetate, natalizumab, siponimod, fingolimod, ocrelizumab, laquinimod **Comparator (reference):** Placebo **Outcome:** Treatment discontinuation due to adverse events **Setting(s):** Outpatient					
**Total studies; total participants**	Relative effect* (95% CI)	Anticipated absolute effect**(95% CI)			**Certainty of the evidence**
		**With placebo**	**With intervention**	**Difference**	
Azathioprine (direct evidence; 1 RCT; 67 participants)	**OR 8.47** (0.42 to 170.95)	**14 per 1000**	**109 per 1000**	**95 more per 1000** (from 8 fewer to 698 more)	⊕◯◯◯ Very low due to imprecision^1^
Rituximab (direct evidence; 2 RCTs; 470 participants)	**OR 4.00** (0.84 to 19.12)	**11 per 1000**	**41 per 1000**	**30 more per 1000** (from 2 fewer to 60 more)	⊕⊕⊕◯ Moderate due to imprecision^2^
Interferon beta‐1a (direct evidence; 4 RCTs; 1455 participants)	**OR 2.93** (1.64 to 5.26)	**25 per 1000**	**70 per 1000**	**45 more per 1000** (from 15 more to 93 more)	⊕⊕⊕⊕ High^3^
Interferon beta‐1b (direct evidence; 2 RCTs; 1657 participants)	**OR 2.98** (1.92 to 4.61)	**41 per 1000**	**112 per 1000**	**71 more per 1000** (from 34 more to 122 more)	⊕⊕⊕◯ Moderate due to imprecision^4^
Immunoglobulins (direct evidence; 2 RCTs; 549 participants)	**OR 1.95** (0.99 to 3.84)	**51 per 1000**	**95 per 1000**	**44 more per 1000** (from 0 more to 120 more)	⊕⊕⊕◯ Moderate due to imprecision^5^
Glatiramer acetate (direct evidence; 1 RCT; 943 participants)	**OR 3.98** (1.48 to 10.72)	**13 per 1000**	**49 per 1000**	**36 more per 1000** (from 6 more to 108 more)	⊕⊕⊕◯ Moderate due to imprecision^6^
Natalizumab (direct evidence; 1 RCT; 888 participants)	**OR 1.02** (0.55 to 1.90)	**47 per 1000**	**74 per 1000**	**1 more per 1000** (from 20 fewer to 39 more)	⊕⊕⊕◯ Moderate due to imprecision^7^
Siponimod (direct evidence; 1 RCT; 1646 participants)	**OR 1.53** (0.98 to 2.38)	**51 per 1000**	**76 per 1000**	**25 more per 1000** (from 1 fewer to 63 more)	⊕⊕⊕◯ Moderate due to imprecision^8^
Fingolimod (direct evidence; 1 RCT; 823 participants)	**OR 2.29** (1.46 to 3.60)	**74 per 1000**	**155 per 1000**	**81 more per 1000** (from 30 more to 149 more)	⊕⊕⊕◯ Moderate due to imprecision^9^
Ocrelizumab (direct evidence; 1 RCT; 725 participants)	**OR 1.24** (0.54 to 2.86)	**33 per 1000**	**41 per 1000**	**8 more per 1000** (from 15 fewer to 57 more)	⊕⊕⊕◯ Moderate due to imprecision^10^
Laquinimod (direct evidence; 1 RCT; 373 participants)	**OR 3.75** (0.83 to 16.99)	**14 per 1000**	**52 per 1000**	**37 more per 1000** (from 2 fewer to 183 more)	⊕⊕◯◯ Low due to imprecision^11^
Placebo	Reference comparator	Not estimable	Not estimable	Not estimable	Reference comparator
*Network meta‐analysis estimates are reported as risk ratio (RR). CI: confidence interval. **Anticipated absolute effect compares 2 risks by calculating the difference between the risk of the intervention group and the risk of the control group. **PMS:** progressive multiple sclerosis; **RCT:** randomised controlled trial					
**GRADE Working Group grades of evidence** **High certainty:** We are very confident that the true effect lies close to that of the estimate of the effect. **Moderate certainty:** We are moderately confident in the effect estimate: the true effect is likely to be close to the estimate of the effect, but there is a possibility that it is substantially different. **Low certainty:** Our confidence in the effect estimate is limited: the true effect may be substantially different from the estimate of the effect. **Very low certainty:** We have very little confidence in the effect estimate: the true effect is likely to be substantially different from the estimate of effect.					

^1^Absolute observed point estimate falls in the trivial negative effect, 95% CI ranges from trivial positive effect to large negative effect; downgraded three levels. ^2^Absolute observed point estimate falls in the trivial negative effect, 95% CI ranges from trivial positive effect to trivial negative effect; downgraded one level.  ^3^Absolute observed point estimate falls in the trivial positive effect, 95% CIs contained within positive effect. ^4^Absolute observed point estimate falls in the trivial negative effect, 95% CI ranges from trivial negative effect to small negative effect; downgraded one level. ^5^Absolute observed point estimate falls in the trivial negative effect, 95% CI ranges from trivial negative effect to small negative effect; downgraded one level. ^6^Absolute observed point estimate falls in the trivial negative effect, 95% CI ranges from trivial negative effect to small negative effect; downgraded one level. ^7^Absolute observed point estimate falls in the trivial negative effect, 95% CI ranges from trivial negative effect to trivial positive effect; downgraded one level. ^8^Absolute observed point estimate falls in the trivial negative effect, 95% CI ranges from trivial negative effect to trivial positive effect; downgraded one level. ^9^Absolute observed point estimate falls in the trivial negative effect, 95% CI ranges from trivial negative effect to small negative effect; downgraded one level. ^10^Absolute observed point estimate falls in the trivial negative effect, 95% CI ranges from trivial negative effect to trivial positive effect; downgraded one level. ^11^Absolute observed point estimate falls in the trivial negative effect, 95% CI ranges from trivial positive effect to small negative effect; downgraded two levels.

## Background

### Description of the condition

Multiple sclerosis (MS) is the most common immune‐mediated, chronic inflammatory demyelinating disease of the central nervous system (CNS). In 85% of affected people, the disease is characterised at onset by relapses followed by complete or partial recovery (relapsing‐remitting phase). Relapses correspond to the clinical expression of focal inflammation and subsequent loss of the myelin sheath surrounding axons in the CNS. In a proportion of patients, increasing with time, the course turns into a secondary progressive phase (SPMS), typically 15 to 20 years from onset. In about 10% to 15% of people affected by MS, the progressive course is not preceded by relapses (primary progressive MS (PPMS)). About 11% of people with PPMS (Montalban 2009) and 40% of those with SPMS (Confavreux 2006) show relapses during the course of the disease. However, new activity becomes less frequent over time, while microglial activation and neurodegeneration become more relevant (Calabrese 2012).

The age of onset of PPMS is typically ~10 years older than relapsing‐remitting MS (RRMS) with a balanced female to male ratio (1:1) (Miller 2007). Other typical features include the clinical presentation with symptoms related to spinal cord involvement (~80%), especially motor, early spinal cord atrophy and the high burden of cortical demyelination (Bieniek 2006; Kutzelnigg 2008), which is usually associated with significant and early impairment of cognition (Chiaravallotti 2008). Of note, PPMS and SPMS share several characteristics, including similar age of presentation and rate of progression over time. These observations support the notion that progressive phenotypes of MS fall within a single disease entity, regardless of whether disability accrual occurs from onset or after a relapsing‐remitting phase.

A recent classification of MS clinical course (or 'phenotype') introduced the concepts of 'disease activity' and 'disease progression' (Lublin 2014). The former is based on the presence of clinical relapse or new or gadolinium‐enhancing magnetic resonance imaging (MRI) lesions. Active forms of MS occur when the inflammatory process is ongoing, sometimes without corresponding clinical manifestations if the inflamed region of the CNS is clinically silent. Disease progression occurs when there is clinical evidence of disability worsening, independent of relapses, over a given period of time, in people who are in a progressive phase of the disease (Lublin 2014). The current classification includes: (i) active or inactive relapsing MS (RMS), with or without worsening; (ii) active or inactive primary progressive MS (PMS) or secondary progressive MS (SPMS), with or without progression; (iii) clinically isolated syndrome (CIS); and (iv) radiologically isolated syndrome (RIS). The definition of 'progressive‐relapsing' MS was abandoned (Lublin 2014).

Furthermore, the concept of MS as a two‐stage disease has recently been questioned by increasing evidence, both from MRI and pathological studies, of a complex interplay between inflammatory and subtle neurodegenerative processes (progression independent of relapse activity (PIRA)) even in the early stages of the disease (Giovannoni 2022). The identification of 'smouldering' progression in a consistent proportion of people with either active or inactive MS demands a more thorough assessment to define progressive MS, with relevant implications for future trials (e.g. appropriate selection of patients in trials on anti‐inflammatory drugs, evaluation of neuroprotective/neurorestorative agents).

MS represents a substantial global health burden, since it affects young people during their productive life, the mean age of diagnosis being 32 years (Walton 2020). The global incidence and prevalence of MS are increasing. From 1990 to 2016, the age‐standardised prevalence of MS increased by 10.4% (9.1 to 11.8). About 2.8 million people worldwide are affected by MS (35.9 per 100,000 population), a figure that has increased by about half‐million since 2013. The global pooled incidence rate is 2.1 per 100,000 persons/year (GBD 2019; Walton 2020).

No current treatment is effective at stopping the natural course of MS towards progressive disability. Current MS treatments include disease‐modifying treatments (DMTs) based on immune‐modulating or immune‐suppressing drugs, which are distinguished from symptomatic drugs for the treatment of specific symptoms of MS (e.g. urinary incontinence or retention, muscular spasms, painful sensitive symptoms). Providing effective and safe treatments for progressive MS (PMS) is particularly challenging due to our incomplete understanding of the pathogenesis of progression. Moreover, while inflammation seems to provide a pivotal contribution to progression, other pathological changes ‐ including cortical demyelination, axonal loss, and mitochondrial dysfunction ‐ also seem important (Dutta 2014; Lassmann 2012), and may represent different therapeutic targets in PMS. Despite several new DMTs becoming available for the treatment of RMS and PMS in recent years, uncertainty remains regarding whether some of them may represent a preferable choice when starting pharmacological treatment, and which ones should be subsequently considered for the management of more advanced stages of the disease course (Reich 2018). As relatively few studies have directly compared different DMTs or assessed the sequential use of specific DMT combinations, clinical practice guidelines on MS treatment usually do not recommend one DMT over another. The variability of recommendations concerning specific drugs among different guidelines reflects in part differences in decisions by regulatory drug agencies and local health policies (Ghezzi 2018).

A previous Cochrane review and network meta‐analysis of randomised clinical trials appraised the available evidence for the efficacy and safety of available DMTs compared to placebo and any other active drug in RMS and PMS (Filippini 2013). The authors concluded that, for the nine disease‐modifying agents used in 18 trials including people with PMS, and the three trials including both relapsing and progressive forms, few studies were of high certainty and no drug was shown to be effective in preventing disability progression in people with MS by pair‐wise or network meta‐analysis (Filippini 2013). The time elapsed since the search date of Filippini 2013 (February 2012) supports the need for an updated analysis, especially given the availability of more DMTs for progressive forms of MS.

### Description of the intervention

DMTs licenced for the treatment of people with MS include the following drugs, which will be considered in our review: beta‐1a and beta‐1b interferon (IFN), pegylated IFN beta‐1a, mitoxantrone, glatiramer acetate, natalizumab, fingolimod, teriflunomide and leflunomide, dimethylfumarate and diroximel fumarate, alemtuzumab, laquinimod, intravenous (iv) immunoglobulins, steroids, ocrelizumab, cladribine, siponimod, ozanimod, ponesimod, ofatumumab, and daclizumab.

Interferon beta (IFNβ) was the first disease‐modifying therapy available and approved in the US in 1993 to treat MS (Hu 2012; Kieseier 2011). Four IFNβ drugs are currently approved in the US and EU: subcutaneous (SC) IFNβ‐1b, SC IFNβ‐1a, intramuscular IFNβ‐1a, and, most recently, in 2014, SC peginterferon beta‐1a. IFNβ‐1b is also licenced in the US and EU for the treatment of active SPMS.

Glatiramer acetate is a synthetic amino acid copolymer, and one of the first approved DMTs for the treatment of RRMS in the US in 1996 (Aharoni 2014). Natalizumab was the first monoclonal antibody licenced for use in MS in 2004 in the US and in 2006 in the EU (Millard 2011). Since then, the monoclonal antibody alemtuzumab has received approval by regulatory agencies for the treatment of RRMS (Kappos 2011; Lycke 2015). Two anti‐CD20 monoclonal antibodies, ocrelizumab and ofatumumab, have also been approved. Ocrelizumab was approved as a treatment for RMS and PPMS (EMA 2018b; FDA 2017), and ofatumumab for RMS and active SPMS (EMA 2021d).

Daclizumab is a monoclonal antibody licenced in 2016 for the treatment of RRMS, but was withdrawn worldwide from the market by its manufacturer in 2018 due to safety concerns (EMA 2018a; FDA 2018).

Cladribine is a synthetic chlorinated deoxyadenosine analogue that was approved for the treatment of RRMS in Russia and Australia in 2010, and licenced in the EU and the US in 2017 and 2019, respectively, for highly active RRMS and active SPMS (EMA 2017; FDA 2019a; Leist 2011).

Fingolimod is a non‐selective modulator of a receptor involved in the sphingosine 1‐phosphate pathway that is administered orally (Chun 2010). It was the first oral treatment approved for RMS in the EU and US, in 2010. More recently, other compounds with a similar mechanism of action have been developed in order to increase efficacy and improve safety, such as siponimod, which was approved in 2019 for active SPMS in the EU and also for RMS in the US (EMA 2020; FDA 2019b), as well as ozanimod and ponesimod, licenced in 2020 and 2021, respectively (EMA 2021a; EMA 2021b; FDA 2020; FDA 2021).

Two other oral drugs, both with a mainly immunomodulatory mode of action, are available for the treatment of RRMS: teriflunomide (Oh 2013), the active metabolite of leflunomide, inhibiting pyrimidine de novo synthesis, and dimethyl fumarate (Linker 2011), the methyl ester of fumaric acid, converted after administration into the active metabolite monomethyl fumarate. They were approved for RRMS in the US in 2012 and in 2013, respectively. Recently, diroximel fumarate, a compound similar to dimethyl fumarate, was approved in 2019 in the US and EU for the treatment of RMS (EMA 2021c).

Laquinimod is an oral immunomodulator investigated in two phase 3 trials for the treatment of people with RRMS. Its use for RRMS was approved in Russia, but in 2014 the European Medicines Agency (EMA) refused authorisation (EMA 2014). Mitoxantrone was approved in 2000 in the US, EU, and other countries for the treatment of people with RRMS and progressive MS (Fox 2004).

Given the limited efficacy of currently available DMTs in delaying the progression of RMS, many clinicians commonly prescribe immunosuppressant drugs with registered indications for conditions other than MS (mainly in rheumatological or autoimmune diseases, or in people undergoing transplant). As such, we decided to also include in our review the following interventions used in MS as off‐label treatments: rituximab, azathioprine, iv immunoglobulins, methotrexate, cyclophosphamide, and long‐term corticosteroids. Rituximab is an anti‐CD20 monoclonal antibody similar to ocrelizumab and ofatumumab that is commonly used to treat malignant blood cell neoplasms and several autoimmune diseases, such as rheumatoid arthritis, idiopathic thrombocytopenic purpura, and pemphigus vulgaris. Its efficacy and safety have also been studied in MS and in several countries, since rituximab is frequently prescribed off‐label (Berntsson 2018; Brancati 2021; Laurson‐Doube 2021). Azathioprine is a purine analogue exerting its immunosuppressive action by affecting DNA replication through inhibition of the synthesis of nucleic acids. It has been used for the treatment of people with MS in many countries based on favourable results reported by placebo‐controlled randomised controlled trials (Laurson‐Doube 2021). Intravenous immunoglobulins are considered in clinical practice for people with RRMS, although evidence on their efficacy in progressive forms is conflicting (Pöhlau 2007; Soelberg Sorensen 2008). Methotrexate, cyclophosphamide, and long‐term corticosteroids are systemic immunosuppressors. Methotrexate is a commonly used treatment in autoimmune diseases. Since 1996, it has been used mainly in the progressive forms of MS. Cyclophosphamide, a DNA‐alkylating agent used for the treatment of people with autoimmune disorders, has also been administered to people with MS (Awad 2009). Long‐term corticosteroids have been proposed for the treatment of people with MS since 1961 with mixed results. They have been administered by different schedules as pulsed periodic high‐dose methylprednisolone or oral continuous low‐dose prednisolone (Ciccone 2008).

### How the intervention might work

The pathophysiology of MS ‐ chronic autoimmune disease of the CNS with inflammatory lesions, demyelination, axonal/neuronal damage, and metabolic changes ‐ supports the use of immunosuppressive medications. Immunosuppressive or immunomodulatory effects are common to all treatments included in this review. Immunotherapies for MS belong to different pharmacological categories, have different modalities of administration (by intramuscular or subcutaneous injection, by infusion or by oral route), and have variable metabolism characteristics. Although all of these drugs target the immune system, their effects vary as follows: (i) immunomodulation (IFNβ‐1b, IFNβ‐1a, glatiramer acetate, pegylated IFNβ‐1a, iv immunoglobulins, dimethyl fumarate and diroximel fumarate, laquinimod); (ii) systemic immunosuppression, inducing a reduction in the activation or efficacy of the immune system through cytostatic or cytotoxic effects (mitoxantrone, methotrexate, cyclophosphamide, long‐term corticosteroids, cladribine, azathioprine, teriflunomide, and leflunomide); and (iii) selective immunosuppression, as with monoclonal antibodies or biological agents directed towards specific antigenic targets (natalizumab, fingolimod, siponimod, ozanimod, ponesimod, alemtuzumab, ofatumumab, daclizumab, rituximab, and ocrelizumab). These aspects must be considered while assessing the risk of adverse events associated with the use of a drug, since safety is usually a consequence of the drug's main pharmacological effect (Compston 2002; Hauser 2020; Massacesi 2002; Meinl 2008).

### Why it is important to do this review

Although there is consensus that immunotherapies reduce the frequency of relapses in MS, the relative benefit of each DMT remains unclear. This uncertainty is in part due to the limited number of head‐to‐head trials, which provide the most rigorous and valid research evidence on the relative effectiveness and safety of different, competing treatments. The estimates from a network meta‐analysis (NMA), including both direct and indirect comparisons, may help to clarify uncertainties and provide valuable information to inform shared healthcare decisions by practitioners, policymakers, people with MS, and their families. Since the most recent Cochrane review concerning MS with NMA (Tramacere 2015), new DMTs have been approved by regulatory agencies, offering a broader spectrum of treatment options for people with PMS. Evidence of efficacy in chronic autoimmune conditions, relatively good tolerability, and reasonable cost have prompted the off‐label use of several immunosuppressants and immunomodulators for the treatment of MS in many countries, particularly in settings with budget constraints (Zeineddine 2020). This is true not only for RMS, but also for progressive forms, for which therapeutic options have been very limited until recently. We therefore decided to also include in the NMA drugs not approved by regulatory agencies.

The data underlying the current review and NMA served as the evidence base for the development of a separate clinical practice guideline on the treatment of RRMS and PMS by an international, highly representative multistakeholder panel (Multiple Sclerosis International Federation (MSIF) Essential Medicines Panel (MEMP)). The panel included people with MS and advocacy group representatives, clinicians from different speciality areas involved in the management of MS, pharmaco‐epidemiologists, and health economists. The guidelines were developed with methodological guidance by the Department of Health Research Methods, Evidence and Impact (HEI), McMaster University, Hamilton, Ontario, Canada, according to the GRADE Working Group method for guideline development (Alonso‐Coello 2016a; Alonso‐Coello 2016b). The MEMP recommendations were used as the evidence base for an application for the inclusion of DMTs in the 23rd World Health Organization Model List of Essential Medicines. The nine critical outcomes identified by MEMP were differentiated into primary and secondary outcomes in this review (see Methods).

## Objectives

To compare through network meta‐analysis the efficacy and safety of alemtuzumab, azathioprine, cladribine, cyclophosphamide, daclizumab, dimethylfumarate, diroximel fumarate, fingolimod, fludarabine, glatiramer acetate, immunoglobulins, interferon beta‐1a and beta‐1b, interferon beta‐1b (Betaferon), interferon beta‐1a (Avonex, Rebif), laquinimod, leflunomide, methotrexate, minocycline, mitoxantrone, mycophenolate mofetil, natalizumab, ocrelizumab, ofatumumab, ozanimod, pegylated interferon beta‐1a, ponesimod, rituximab, siponimod, corticosteroids, and teriflunomide for progressive multiple sclerosis.

## Methods

### Criteria for considering studies for this review

#### Types of studies

We included individually randomised parallel controlled clinical trials (RCTs). We considered studies published in abstract form when sufficient information was available on study design, characteristics of participants, interventions, and outcomes. We included studies with a follow‐up of 12 months or longer. We did not include cluster‐randomised and cross‐over trials, case reports, and studies of within‐group design, such as before‐after (pre‐post) studies with no control group or interrupted time series.

#### Types of participants

We included adult participants (18 years or older) with a diagnosis of PMS, adopting any published diagnostic criteria, of either sex, who were treatment‐naive or non‐responsive to treatment with previous DMTs, regardless of degree of disability and disease duration. We accepted any definition of non‐response reported in the included studies. We considered both treatment‐naive people with MS, and those switching from a previous different DMT, regardless of the reason for switching, method, or timing of the switching. We considered studies primarily focused on PMS but also including a subgroup of people with RMS only if the proportion of people with PMS was ≥ 80%. We considered downgrading the certainty of the evidence from studies including 80% to 99% of people with PMS for indirectness when performing the GRADE assessment (Guyatt 2011).

#### Types of interventions

We considered DMTs used to treat people with MS (even if not licenced in any country). We considered regimens as defined in primary studies, irrespective of their dose. We considered the following treatments: alemtuzumab, azathioprine, cladribine, cyclophosphamide, daclizumab, dimethylfumarate, diroximel fumarate, fingolimod, fludarabine, glatiramer acetate, immunoglobulins, interferon beta‐1a and beta‐1b, interferon beta‐1b (Betaferon), interferon beta‐1a (Avonex, Rebif), laquinimod, leflunomide, methotrexate, minocycline, mitoxantrone, mycophenolate mofetil, natalizumab, ocrelizumab, ofatumumab, ozanimod, pegylated interferon beta‐1a, ponesimod, rituximab, siponimod, corticosteroids, and teriflunomide.

We considered long‐term corticosteroids (i.e. longer than six months) of any type of corticosteroid, continuous or intermittent, provided that they were not started for relapses (i.e. started more than two months after a relapse), whatever the administration route and dosage.

We assumed that treatments were 'jointly randomisable' across trial participants (Salanti 2012).

We did not include: combination treatments, trials in which a drug regimen was compared with a different regimen of the same drug without another active agent or placebo as a control arm, all non‐pharmacological treatments, or interventions with over‐the‐counter drugs.

##### Types of comparisons

We considered placebo, no treatment, or another active agent as comparator. Studies comparing placebo and no treatment were grouped into a single node in the network plot.

#### Types of outcome measures

While defining the outcomes for our review, we searched the Core Outcome Measures in Effectiveness Trials (COMET) core outcome set (COS) database (www.comet-initiative.org) and found the following COS covering the topic of pharmacological treatments in MS: one protocol of an ongoing COS project on DMTs in RCTs on RMS (Lucchetta 2020), one unpublished ongoing COS (S.O.S.MS Project 2020), one COS for clinical trials or clinical research on children with MS (Chitnis 2013), and one COS on MS therapeutic trial aimed at identifying the most important aspects of clinical evaluation, study design, and data analysis (Whitaker 1995).

We estimated the relative effects of the competing interventions according to the following primary outcomes.

The measurement of at least one of our predefined outcomes was an inclusion criterion for the review.

##### Primary outcomes

###### Efficacy

- **Relapses:** number of participants with clinical relapses based on clinical follow‐up visits at 12, 24, and 36 months after randomisation. Relapse was defined as the appearance of one or more new symptoms due to MS, or the deterioration of pre‐existing symptoms, persisting more than 24 hours in the absence of fever, and preceded by a period of stability of at least one month (McDonald 2001).
- **Disability:** number of participants with sustained disability worsening based on clinical follow‐up visits at 24 and 36 months after randomisation. Worsening was defined as at least one increased point on the Expanded Disability Status Scale (EDSS) (Kurtzke 1983), or a 0.5‐point increase if the baseline EDSS was greater than 5.5, confirmed during two consecutive clinical examinations separated by an interval of at least six months free from relapse, and carried out by the same physician. EDSS is an ordinal scale where 0 is normal, 3 indicates mild disability, 6 indicates care requirement, 7 indicates wheelchair use, and 10 indicates death. An advantage of the EDSS over other disability measures is its international acceptance, e.g. by the EMA (EMA 2015), as a primary endpoint in clinical trials. It is also widely used in trials, enabling cross‐study comparisons (Meyer‐Moock 2014).

###### Safety

- **Serious adverse events (SAEs):** number of participants with any (one or more) SAEs during the trial, defined according to study authors. If sufficient information is available, we will specify individual SAEs.
- **Treatment discontinuation due to adverse events:** number of people who discontinued treatment due to adverse events during the trial, regardless of their severity.

##### Secondary outcomes

- **New gadolinium‐enhancing positive T1‐weighted MRI lesions:** number of participants with new gadolinium‐enhancing T1‐weighted MRI lesions at 12, 24, and 36 months after randomisation.
- **New or enlarging T2‐weighted MRI lesions:** number of participants with new or enlarging T2‐weighted MRI lesions at 12, 24, and 36 months after randomisation.
- **Cognitive decline**: assessed as a continuous outcome considering the variation in the score of the Symbol Digit Modalities Test (SDMT) when available (Benedict 2017), or alternatively, the Paced Auditory Serial Addition Test (PASAT) (Gronwall 1977). Cognitive decline measured with other validated scales was qualitatively described. We considered the longest time point reported in the study.
- **Quality of life impairment:** assessed as a continuous outcome considering the variation in the score of scales reporting quality of life impairment. We considered any available scale. We considered the longest time point reported in the study.
- **Mortality:** overall number of MS‐related deaths.

### Search methods for identification of studies

All searches were designed and conducted by Chiara Bassi, Information Specialist for Cochrane Multiple Sclerosis and Rare Diseases of the CNS Group, with input from Robin Featherstone, Information Specialist, Cochrane Central Executive Team.

#### Electronic searches

We identified eligible study references through systematic searches of the following bibliographic databases.

- Cochrane Central Register of Controlled Trials (CENTRAL; 2022, Issue 8) in the Cochrane Library (see Appendix 1 for search string).
- MEDLINE (PubMed) (January 2012 to 8 August 2022) (see Appendix 2 for search string).
- Embase (Embase.com) (January 2012 to 8 August 2022) (see Appendix 3 for search string).

We did not apply any search limitations with respect to study outcomes, methods of analysis, or language.

#### Searching other resources

To identify eligible studies prior to 2012, we consulted the identified studies in Filippini 2013, a prior Cochrane NMA review concerning immunomodulators and immunosuppressants for MS, whose search was performed February 2012.

We searched for ongoing studies on the following trial registries.

- World Health Organization International Clinical Trials Registry Platform (WHO ICTRP) (apps.who.int/trialsearch). Search terms: progressive multiple sclerosis, filtered for "Phase 2" "Phase 3" trials.
- US National Institutes of Health Ongoing Trials Register ClinicalTrials.gov (www.clinicaltrials.gov). Search term: "progressive multiple sclerosis".

We checked the reference lists of all included studies and any relevant systematic reviews identified for additional references to studies. We planned to examine any relevant retraction statements and errata for included studies.

### Data collection and analysis

#### Selection of studies

We conducted study selection using the Rayyan platform (rayyan.ai) in accordance with the methods described in the *Cochrane Handbook for Systematic Reviews of Interventions* (Higgins 2021). Six review authors in pairs (BR, EB, FN, GP, IT, MF) independently screened the titles and abstracts for potentially relevant articles. We obtained the full‐text reports of those articles deemed potentially relevant, and the same six review authors in pairs assessed these for inclusion in the review.

#### Data extraction and management

Two review authors (SM, MGL) independently extracted data from the included studies using a predefined data extraction form in a Microsoft Excel spreadsheet and piloted the data extraction form on five studies in the review (Microsoft Excel). Any disagreements were resolved by discussion or by consulting a third review author (FN) as necessary. When data were available from peer‐reviewed journals as full publication as well as from trials registries (such as ClinicalTrials.gov or the WHO ICTRP), we extracted data from the former. We extracted results data from the trials registries when these were the only available data.

We extracted the following information from each included study.

- **Study**: first author or acronym, number of centres, year of publication, years that the study was conducted (recruitment and follow‐up), publication (full‐text publication, abstract publication, unpublished data).
- **Study design:** inclusion criteria, number of randomised participants, duration of follow‐up (12, 24, or 36 months).
- **Population**: baseline mean age, gender, definition of relapse.
- **Potential effect modifiers**: diagnostic criteria (Poser or McDonald criteria); previous treatments with DMTs, by structuring four categories: 'no previous treatment with DMTs', 'previous treatment with DMTs', 'uncertain information on previous treatment with DMTs', and 'mixed population of patients, previously treated and previously untreated with DMTs'; type of MS (active versus non‐active).
- **Interventions**: active agent, dose, frequency, or duration of treatment.
- **Funding source**.

For dichotomous outcomes, we extracted the number of participants experiencing the event of interest over the number of randomised participants.

For continuous outcomes relative to the outcomes cognitive decline and quality of life impairment, we extracted mean and standard deviation of the comparison groups, where possible. We extracted data at baseline, endpoint, and change score. We used change score if endpoint scores were not reported (da Costa 2013). We extracted data at the authors' defined timing points.

When outcomes were not reported at our predefined time points, we extracted data as close as possible to that time point.

We did not seek translation of any records in order to extract data as this was not necessary.

#### Assessment of risk of bias in included studies

Two review authors (SM, MGL) independently assessed risk of bias in the included studies using Cochrane's RoB 1 tool (Higgins 2017), which is based on the following domains: random sequence generation, allocation concealment, blinding of participants and personnel, blinding of outcome assessor, incomplete outcome data, and selective outcome reporting. We judged the risk of bias for each domain as low, high, or unclear risk of bias.

Other potential risks of bias included the role of the sponsor; we judged a study as at high risk of bias if it was funded by industry, and it was stated that the funder was involved in data management, analysis, and interpretation; in writing of the study report; or where it was reported that the funders approved the final version of the paper. We also judged a study as being at high risk of bias if the first or last authors and authors who performed the statistical analysis were employed by industry.

We judged incomplete outcome data as at low risk of bias if numbers and causes of dropouts were balanced (i.e. in the absence of a significant difference) between arms. We assessed selective outcome reporting bias by comparing outcomes reported in the study protocol with the published outcome results. If a study protocol was not available, we assigned a judgement of unclear risk of bias. If the study protocol was available, but was not dated prior to the start of the study, we judged the study as at high risk of bias. We judged a study as at high risk for selective reporting if the authors failed to report complete data for one or more outcomes (e.g. reported the P value only, or simply stated that the results were or were not statistically significant).

Any disagreements between review authors were resolved by discussion to reach consensus.

#### Measures of treatment effect

##### Relative treatment effects

For dichotomous outcomes (i.e. disability and relapses), we reported risk ratio (RR) and 95% confidence intervals (CIs). If the number of observed events was small (less than 5% of sample per group), and if studies had balanced treatment groups, we reported the Peto odds ratio (OR) with 95% CI.

For continuous outcomes, we calculated mean difference (MD), or standardised mean difference (SMD) if the same continuous outcome was measured with different metrics. To interpret SMD we used the guiding principles of thresholds for small (SMD = ±0.2), moderate (SMD = ±0.5), and large effects (SMD = ±0.8) (Schünemann 2013). We presented results from NMA as summary relative effect sizes (RR, MD, or SMD) for each possible pair of treatments.

##### Relative treatment ranking

We obtained a treatment hierarchy of the included interventions using the surface under the cumulative ranking curve (SUCRA) and mean ranks. SUCRA was expressed as a percentage and represents the relative probability of a treatment to be among the best options without uncertainty (Salanti 2011).

#### Unit of analysis issues

Cluster‐randomised and cross‐over trials were not eligible for inclusion in the review.

##### Studies with multiple treatment groups

For pair‐wise meta‐analysis, we considered the multi‐arm studies as multiple independent two‐arm studies. For NMA, we accounted for the correlation between the effect sizes from multi‐arm studies (Salanti 2012). For studies with multi‐arm trials involving the same agent at different doses compared to a control treatment, we converted the treatment arms into a single arm by merging the different doses, summing the number of events, and calculating the sample size.

##### Studies with multiple outcome scales

MS‐specific scales (e.g. Multiple Sclerosis Quality of Life (MSQOL)‐54 Instrument, Multiple Sclerosis Impact Scale (MSIS‐29)) were not combined with non‐MS‐specific scales (e.g. 36‐Item Short Form Health Survey (SF‐36) or EQ‐5D index). Where several scales are used in one RCT, we selected the scale that provided lower heterogeneity in combination (via SMD) with the others across studies.

#### Dealing with missing data

We used data that reflected the intention‐to‐treat (ITT) analysis for each outcome. We performed primary analysis considering the number of participants with the event in relation to the number of randomised participants. In the case of participants with missing data, we performed primary analysis without any imputation. For adverse events, we used data from participants who received at least one dose of the study medication. Where standard deviations were missing for continuous outcomes, we calculated them according to the methods described in the *Cochrane Handbook for Systematic Reviews of Interventions* (Higgins 2019).

#### Assessment of heterogeneity

##### Assessing clinical and methodological heterogeneity within and across comparisons of drugs

In each pair‐wise comparison, patient characteristics, treatments, and outcome definition of included studies should be similar. We produced descriptive statistics for studies and assessed their similarity in each comparison. It is appropriate to use NMA if the assumption of transitivity can be defended, for example there is agreement between drug effects estimated directly and indirectly for a specific comparison. Transitivity holds when the distributions of the potential effect modifiers, like study and patient‐level covariates, are balanced across all pair‐wise comparisons; in this case, direct and indirect evidence can be combined. As such, we compared the distribution of potential effect modifiers across different pair‐wise comparisons (Cipriani 2013; Salanti 2012).

#### Assessment of reporting biases

For primary outcomes, we used a comparison‐adjusted funnel plot for active treatments versus placebo to determine the possibility of small‐study effects (Chaimani 2013; Peters 2008).

#### Data synthesis

Firstly, we conducted conventional pair‐wise meta‐analyses with a random‐effects model in RevMan software for each outcome and comparisons with at least two studies (DerSimonian 1986; RevMan 2024). Then, we performed NMA in a frequentist framework for each outcome with a random‐effects model using the 'mvmeta' command in Stata, accounting for correlations induced by multi‐arm studies (Salanti 2012; White 2011). NMA is a statistical method used to synthesise information from a network of trials addressing the same question but involving different interventions (Cipriani 2013). NMA combines direct and indirect evidence across a network of randomised trials into a single effect size, and under certain assumptions, it can increase the precision in the estimates while randomisation is respected.

When we could not pool results from included studies quantitatively via pair‐wise or NMA, we undertook narrative synthesis according to the Synthesis Without Meta‐analysis (SWiM) reporting guideline (Brennan 2020).

#### Subgroup analysis and investigation of heterogeneity

##### Assessing and investigating statistical heterogeneity and incoherence

We estimated heterogeneity variances for each pair‐wise comparison in standard pair‐wise meta‐analyses and assessed the presence of statistical heterogeneity by visually inspecting the forest plots, looking at the Chi^2^, and calculating the I^2^ statistic (Higgins 2003). In NMA, we assumed a common estimate for heterogeneity variance across comparisons and based our assessment of statistical heterogeneity in the whole network on the magnitude of the common heterogeneity parameter (Rhodes 2015; Turner 2012). We evaluated statistical disagreement between direct and indirect effect sizes (incoherence) with local and global approaches (Higgins 2012). Locally, we used the loop‐specific approach, which calculates the difference between direct and indirect estimates in all closed loops in the network (Veroniki 2013). We also applied the node‐splitting method, which separates evidence on a particular comparison into direct and indirect evidence (Higgins 2012). Globally, we planned to apply the 'design‐by‐treatment' approach (Higgins 2021).

##### Subgroup analyses

We planned to perform subgroup analyses for primary efficacy outcomes among people with previous lack of response to treatment, based on the type of PMS course (active PMS, not‐active PMS, stable PMS, worsening PMS, active and worsening PMS or indeterminate PMS).

We were unable to perform our planned subgroup analyses because all the studies included only people with active PMS, and because the definition of previous lack of response to treatment varied among studies.

#### Sensitivity analysis

We planned to perform the following sensitivity analyses for our primary efficacy outcomes:

- including only trials with low risk of selection bias (allocation concealment) and attrition bias;
- excluding trials with a total sample size of fewer than 50 randomised participants.

However, these sensitivity analyses were precluded by an insufficient number of studies meeting these criteria.

#### Summary of findings and assessment of the certainty of the evidence

We assessed the certainty of the evidence for the included RCTs for the NMA by means of the GRADE methodology, considering the following domains: risk of bias, inconsistency, indirectness, imprecision, incoherence, and publication bias. Firstly, direct and indirect estimates of effect for the pair‐wise comparisons were presented, then the certainty of both of these estimates was rated, the network estimate for the pair‐wise comparison presented, and finally the certainty of the network estimate was rated, based on the ratings of the direct and indirect estimates and the assessment of coherence (i.e. extent of similarity of direct and indirect estimates) (Puhan 2014).

Since the results of this review and NMA will serve as the evidence base for guidance on the use of DMTs in people with PMS, the certainty of the evidence for this review was assessed using a fully contextualised approach. A fully contextualised approach is important in an NMA to incorporate the value of individual outcomes in the overall interpretation of the results (Schünemann 2022). In this review, this involved predefining quantitative thresholds to determine the magnitude of each health effect (desirable or undesirable) measured by means of each outcome. The magnitudes for desirable and undesirable health effects were defined according to the GRADE wording as 'trivial', 'small', 'moderate', and 'large'.

For this NMA, we used outcomes assessed by the MSIF Essential Medicines Panel, which was convened to make recommendations on essential medicines for MS. The value of the outcomes was assessed by the guideline panel to judge both the priority of outcomes (not important/important/critical) and a health state utility value (HSUV) corresponding to the outcome in question. The panel identified nine critical outcomes. In this review, the authors further differentiated and reported those outcomes as four primary and five secondary outcomes. The HSUV was derived from a review of reviews or panel judgement if not identified from the literature. The HSUV is utilised to calculate thresholds for the magnitude of effects. The thresholds between trivial/small (T1), small/moderate (T2), and moderate/large (T3) were predefined through calculation informed by the HSUV of each outcome (Appendix 4). The threshold coefficient was derived from an interim analysis of an ongoing global survey on decision thresholds across assessing respondent judgements across varied disease category examples (Morgano 2022).

We followed GRADE guidance for assessing imprecision using a fully contextualised approach (Schünemann 2022). In order to determine the imprecision of estimates, and therefore make imprecision judgements including downgrading by one, two, or three levels for certainty, point estimates of observed effects and their 95% CIs were contextualised in relation to the predefined thresholds (Hultcrantz 2017). In accordance with the GRADE guidance on imprecision, overall imprecision of interventions was assessed across all critical/important outcomes with guideline panel input. If most outcomes were not downgraded for imprecision, the overall certainty was not necessarily downgraded to the lowest certainty (Schünemann 2022).

We performed evaluation of direct evidence from pair‐wise comparisons on the GRADEpro GDT platform (GRADEpro GDT).

We manually developed summary of findings tables for NMA presenting network geometry plots, estimates of effects, credible intervals, and certainty of the evidence according to the format suggested by the GRADE Working Group (Yepes‐Nuñez 2019). We developed a summary of findings table for each outcome, including all interventions with estimates available from direct or indirect comparisons.

We included an overall grading of the evidence for the following outcomes for the comparison with placebo as common comparators:

- proportion of participants who experienced new relapses over 12, 24, and 36 months;
- proportion of participants who experienced disability worsening over 24 and 36 months;
- proportion of participants who discontinued treatment due to adverse events;
- proportion of participants with any (one or more) SAEs, defined according to study authors.

Where we were not able to perform the NMA, we have presented results from simple pair‐wise estimates for each treatment versus placebo.

## Results

### Description of studies

#### Results of the search

We identified 17,768 reports through an electronic database search dated 8 August 2022, of which 13,031 reports remained after duplicates were removed (Figure 1). We also added for consideration 44 included reports from another relevant NMA review that included studies with people with MS (Filippini 2013). We carried forward 13,031 reports for screening, of which 12,981 were excluded based on title/abstract. We evaluated 50 full texts as potentially meeting inclusion criteria and excluded 11 with reasons provided, and identified 10 reports of ongoing trials. We included a total of 23 studies (29 reports) in the review.

**1:**
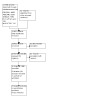
PRISMA flow chart.

#### Included studies

We included 23 studies involving 10,167 participants and published between 1989 and 2022. Fifteen of these studies (Anderson 2004; Bornstein 1991; Ellison 1989; Etemadifar 2019; European Study Group 1998; Goodkin 1995; Hawker 2009; Hommes 2004; IMPACT 2002; Leary 2003; Montalban 2009; NASP 2004; Pohlau 2007; SPECTRIMS 2001; Wolinsky 2007) were included in a previous Cochrane review and network meta‐analysis of immunomodulators and immunosuppressants in people with progressive multiple sclerosis (Filippini 2013). The current review also includes eight new studies (ARPEGGIO 2020; ASCEND 2018; Cheshmavar 2020; EXPAND 2018; INFORMS 2016; Komori 2016; ORATORIO 2017; PROMESS 2017).

All studies were performed in outpatient settings. The mean age of participants was 44 years, and 5812 participants (57%) were female. Of the 23 included studies, four studies included a mixed population of patients with and without previous treatment with DMTs (ARPEGGIO 2020; EXPAND 2018; Hawker 2009; ORATORIO 2017); one study included a population without previous treatment with DMTs (Montalban 2009); and the remaining 18 studies did not report data about previous treatments with DMTs.

Median follow‐up was 24 months (including 60‐month follow‐up (1 study); 36‐month follow‐up (5 studies); 33‐month follow‐up (1 study); 30‐month follow‐up (1 study); 27‐month follow‐up (1 study); 24‐month follow‐up (12 studies); 12‐month follow‐up (2 studies)). Twenty studies were placebo‐controlled, and three were head‐to‐head studies. Funding came from industry in 17 studies, from public sources in four cases, and was not reported in two cases. See Characteristics of included studies for further details.

We identified 10 ongoing studies that will be considered for inclusion in future updates of this review (EUCTR2012‐003056‐36; EUCTR2014‐003021‐18‐PL; EUCTR2018‐001511‐73‐ES; EUCTR2018‐001511‐73‐GB; EUCTR2018‐005038‐39‐GB; EUCTR2020‐002981‐15‐DK; IRCT20130812014333N125; NCT04035005; NCT04688788; NCT04695080). See Characteristics of ongoing studies for further details.

#### Excluded studies

We excluded 11 studies after full‐text review (see Characteristics of excluded studies). Seven of these studies were included in a previous review (Filippini 2013), but were excluded here for the following reasons: mixed samples with < 80% of participants with progressive forms of MS (British and Dutch 1988; Hartung 2002; Milanese 1993); insufficient treatment duration/follow‐up (CCMSSG 1991; Edan 1997); wrong publication type (Ghezzi 1989); MS phenotype (relapsing/progressive) unclear (Miller 1961). The remaining four excluded studies were post hoc subanalyses or extensions that did not meet our inclusion criteria (Evdoshenko 2019; Fox 2018; Kuhle 2016; Wolinsky 2018).

### Risk of bias in included studies

Risk of bias summaries are provided in Figure 2 and Figure 3.

**2:**
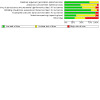
Risk of bias graph: review authors' judgements about each risk of bias item presented as percentages across all included studies.

**3:**
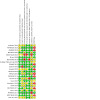
Risk of bias summary: review authors' judgements about each risk of bias item for each included study.

#### Allocation

##### Random sequence generation

Six studies (26%) provided insufficient information to assess sequence generation (unclear risk) (Anderson 2004; ARPEGGIO 2020; ASCEND 2018; Cheshmavar 2020; Hawker 2009; Wolinsky 2007), while the remaining 17 studies (74%) reported adequate methods (low risk).

##### Allocation concealment

One trial (4.3%) used an unconcealed procedure (high risk) (Goodkin 1995). Eleven studies (47.8%) provided insufficient information to permit a risk of bias judgement (unclear risk) (Anderson 2004; Cheshmavar 2020; Hawker 2009; Hommes 2004; IMPACT 2002; Komori 2016; Leary 2003; Montalban 2009; NASP 2004; Pohlau 2007; Wolinsky 2007). The remaining 11 studies (47.8%) reported adequate methods of allocation concealment (low risk).

#### Blinding

##### Performance bias

Two studies (8.7%) were not blinded (high risk) (Cheshmavar 2020; Etemadifar 2019). Nine studies (39.1%) provided insufficient information to permit a risk of bias assessment (unclear risk) (Anderson 2004; Bornstein 1991; Hawker 2009; IMPACT 2002; Komori 2016; Montalban 2009; NASP 2004; ORATORIO 2017; Wolinsky 2007). The remaining 12 studies (52.2%) reported that participants and investigators were blinded (low risk).

##### Detection bias

One study (4.35%) was at high risk (Cheshmavar 2020). Four studies (17.39%) provided insufficient information to permit a risk of bias judgement (unclear risk) (Hawker 2009; Komori 2016; Montalban 2009; Pohlau 2007). The remaining 18 studies (78.26%) reported that outcome assessors were blinded, resulting in a judgement of low risk of detection bias.

#### Incomplete outcome data

Two studies (8.7%) were at high risk of bias due to incomplete outcome data (high number of dropouts, unbalanced across intervention groups) (Etemadifar 2019; Komori 2016). We assessed the remaining 21 studies (91.3%) to be at low risk of bias.

#### Selective reporting

We judged two studies (8.7%) as at high risk of bias for selective reporting because not all prespecified primary benefit outcomes were reported on (Cheshmavar 2020; PROMESS 2017). We judged 13 studies (56.5%) as at unclear risk of reporting bias due to lack of a protocol (Anderson 2004; Bornstein 1991; Ellison 1989; Etemadifar 2019; European Study Group 1998; Goodkin 1995; IMPACT 2002; Leary 2003; Montalban 2009; NASP 2004; ORATORIO 2017; SPECTRIMS 2001; Wolinsky 2007). The remaining eight studies (34.8%) reported all prespecified primary benefit outcomes and were judged as at low risk of bias.

#### Other potential sources of bias

We judged nine studies (39.1%) as at high risk of other bias because of the role of the sponsor in authorship of the study report or in data management or analysis (ARPEGGIO 2020; ASCEND 2018; EXPAND 2018; Hawker 2009; IMPACT 2002; INFORMS 2016; Montalban 2009; ORATORIO 2017; SPECTRIMS 2001ARPEGGIO 2020; ASCEND 2018; EXPAND 2018; Hawker 2009; IMPACT 2002; INFORMS 2016; Montalban 2009; ORATORIO 2017; SPECTRIMS 2001). We judged seven studies (30.4%) as at unclear risk of bias for this domain because the role of the study sponsor was unclear (Anderson 2004; Etemadifar 2019; European Study Group 1998; Leary 2003; NASP 2004; Pohlau 2007; Wolinsky 2007). We judged the remaining seven studies (30.4%) as at low risk of other bias.

### Effects of interventions

See: Table 1; Table 2; Table 3; Table 4; Table 5; Table 6; Table 7

The summary of findings tables provide overall estimates of treatment effects compared with placebo, and the certainty of the available evidence obtained through network meta‐analyses for the five efficacy outcomes (chance of experiencing one or more relapses over 12 months, chance of experiencing one or more relapses over 24 months, chance of experiencing one or more relapses over 36 months, chance of disability getting worse over 24 months, chance of disability getting worse over 36 months) and the two safety outcomes (discontinuation due to adverse events and SAEs). See Table 1; Table 2; Table 3; Table 4; Table 5; Table 6; Table 7.

The networks' geometry for the efficacy and safety of immunomodulators and immunosuppressants included in the review is shown in Figure 4; Figure 5. Each line links the treatments that have been directly compared in studies. The thickness of the line is proportional to the number of participants included in the comparison, and the width of each circle is proportional to the number of studies included in the comparison.

**4:**
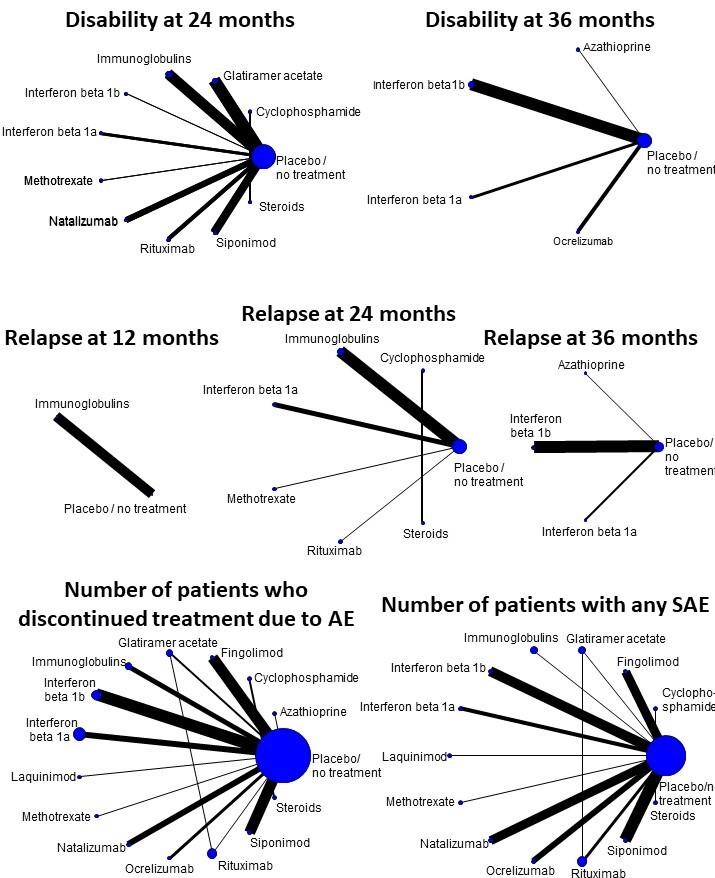
Network plots of treatment comparisons for benefit and safety ‐ primary outcomes. The width of the lines is proportional to the precision of each pair of treatments, and the size of every circle is proportional to the number of trials comparing every pair of treatments. AE, adverse events; SAE, serious adverse events

**5:**
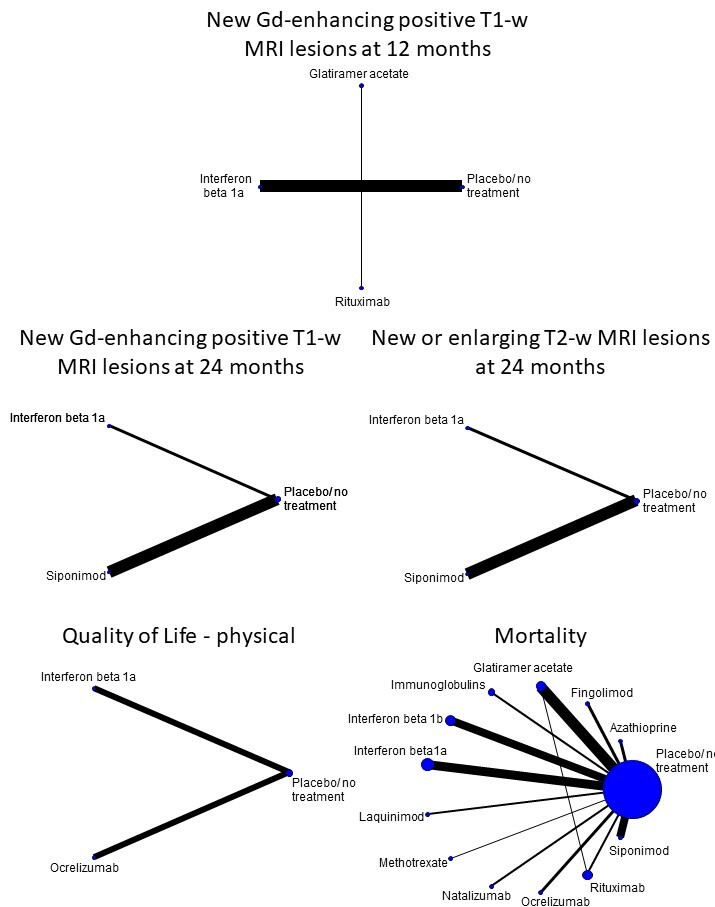
Network plots of treatment comparisons for benefit and acceptability ‐ secondary outcomes. The width of the lines is proportional to the precision of each pair of treatments, and the size of every circle is proportional to the number of trials comparing every pair of treatments. Gd, gadolinium; w, weighted

Network estimates of efficacy and safety of the primary outcomes for each treatment against placebo within the networks are shown in Figure 6. Network estimates of benefit and safety of primary outcomes for each treatment against placebo and against each other treatment included in the network are shown in Table 8; Table 9; Table 10; Table 11; Table 12; Table 13.

**6:**
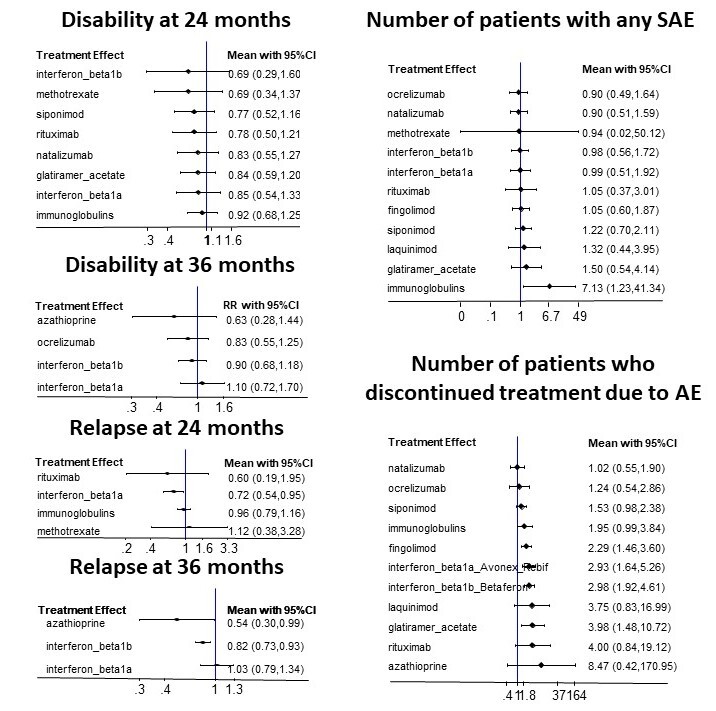
Network meta‐analysis estimates of treatment benefit against placebo. AE, adverse events; CI, confidence interval; RR, risk ratio; SAE, serious adverse events

**1:** Netleague: Relapse (24 months)*

**Rituximab**	1.66 (0.51,5.33)	1.86 (0.38,9.09)	1.19 (0.36,3.97)	1.58 (0.48,5.18)
0.60 (0.19,1.95)	**Placebo**	1.12 (0.38,3.28)	**0.72 (0.54,0.95)**	0.96 (0.79,1.16)
0.54 (0.11,2.63)	0.89 (0.30,2.61)	**Methotrexate**	0.64 (0.21,1.94)	0.85 (0.29,2.53)
0.84 (0.25,2.80)	**1.39 (1.05,1.85)**	1.56 (0.51,4.74)	**Interferon beta‐1a**	1.33 (0.95,1.87)
0.63 (0.19,2.07)	1.05 (0.87,1.27)	1.17 (0.39,3.49)	0.75 (0.53,1.06)	**Immunoglobulins**

*Significant results are bolded and underlined.

**2:** Netleague: Relapse (36 months)*

**Placebo**	**0.82 (0.73,0.93)**	1.03 (0.79,1.34)	**0.54 (0.30,0.99)**
**1.22 (1.08,1.37)**	**Interferon beta‐1b**	1.25 (0.94,1.67)	0.66 (0.36,1.21)
0.97 (0.75,1.26)	0.80 (0.60,1.06)	**Interferon beta‐1a**	0.53 (0.27,1.01)
**1.84 (1.01,3.35)**	1.52 (0.83,2.79)	1.90 (0.99,3.65)	**Azathioprine**

*Significant results are bolded and underlined.

**3:** Netleague: Disability (24 months)

**Siponimod**	1.00 (0.55,1.83)	1.29 (0.86,1.93)	1.08 (0.60,1.92)	0.89 (0.40,1.97)	0.88 (0.35,2.26)	1.09 (0.60,2.00)	1.19 (0.72,1.96)	1.08 (0.63,1.85)
1.00 (0.55,1.82)	**Rituximab**	1.29 (0.82,2.01)	1.07 (0.58,1.98)	0.88 (0.39,2.01)	0.88 (0.34,2.29)	1.09 (0.58,2.05)	1.18 (0.69,2.03)	1.08 (0.61,1.91)
0.77 (0.52,1.16)	0.78 (0.50,1.21)	**Placebo/no treatment**	0.83 (0.55,1.27)	0.69 (0.34,1.37)	0.69 (0.29,1.60)	0.85 (0.54,1.33)	0.92 (0.68,1.25)	0.84 (0.59,1.20)
0.93 (0.52,1.66)	0.93 (0.51,1.71)	1.20 (0.79,1.82)	**Natalizumab**	0.82 (0.37,1.84)	0.82 (0.32,2.11)	1.01 (0.55,1.87)	1.10 (0.66,1.84)	1.00 (0.58,1.74)
1.13 (0.51,2.51)	1.13 (0.50,2.57)	1.46 (0.73,2.90)	1.22 (0.54,2.72)	**Methotrexate**	1.00 (0.34,2.98)	1.23 (0.54,2.81)	1.34 (0.63,2.85)	1.22 (0.56,2.66)
1.13 (0.44,2.89)	1.13 (0.44,2.95)	1.46 (0.63,3.40)	1.22 (0.47,3.13)	1.00 (0.34,2.98)	**Interferon beta‐1b**	1.23 (0.47,3.22)	1.34 (0.55,3.30)	1.22 (0.49,3.07)
0.92 (0.50,1.68)	0.92 (0.49,1.73)	1.18 (0.75,1.86)	0.99 (0.53,1.83)	0.81 (0.36,1.85)	0.81 (0.31,2.12)	**Interferon beta‐1a**	1.09 (0.63,1.88)	0.99 (0.56,1.77)
0.84 (0.51,1.39)	0.84 (0.49,1.45)	1.09 (0.80,1.47)	0.91 (0.54,1.52)	0.75 (0.35,1.58)	0.74 (0.30,1.83)	0.92 (0.53,1.58)	**Immunoglobulins**	0.91 (0.57,1.46)
0.92 (0.54,1.58)	0.93 (0.52,1.64)	1.19 (0.83,1.71)	1.00 (0.57,1.73)	0.82 (0.38,1.78)	0.82 (0.33,2.05)	1.01 (0.57,1.80)	1.10 (0.69,1.76)	**Glatiramer acetate**

**4:** Netleague: Disability (36 months)

**Placebo/no treatment**	0.83 (0.55,1.25)	0.90 (0.68,1.18)	1.10 (0.72,1.70)	0.63 (0.28,1.44)
1.21 (0.80,1.82)	**Ocrelizumab**	1.08 (0.66,1.78)	1.33 (0.73,2.42)	0.77 (0.31,1.91)
1.11 (0.85,1.47)	0.92 (0.56,1.51)	**Interferon beta‐1b**	1.23 (0.74,2.05)	0.71 (0.30,1.67)
0.91 (0.59,1.40)	0.75 (0.41,1.36)	0.81 (0.49,1.36)	**Interferon beta‐1a**	0.58 (0.23,1.45)
1.58 (0.70,3.57)	1.31 (0.52,3.26)	1.42 (0.60,3.35)	1.74 (0.69,4.38)	**Azathioprine**

**5:** Netleague: Serious adverse events*

**Siponimod**	0.86 (0.26,2.83)	0.82 (0.48,1.42)	0.74 (0.33,1.67)	0.74 (0.34,1.63)	0.77 (0.01,42.76)	1.08 (0.32,3.70)	0.81 (0.37,1.77)	0.82 (0.35,1.93)	5.86 (0.93,36.92)	1.23 (0.39,3.91)	0.87 (0.39,1.91)
1.16 (0.35,3.79)	**Rituximab**	0.95 (0.33,2.73)	0.85 (0.25,2.87)	0.86 (0.26,2.83)	0.89 (0.01,54.67)	1.26 (0.27,5.74)	0.93 (0.28,3.08)	0.95 (0.27,3.27)	6.78 (0.86,53.29)	1.42 (0.36,5.65)	1.00 (0.30,3.32)
1.22 (0.70,2.11)	1.05 (0.37,3.01)	**Placebo/no treatment**	0.90 (0.49,1.64)	0.90 (0.51,1.59)	0.94 (0.02,50.12)	1.32 (0.44,3.95)	0.98 (0.56,1.72)	0.99 (0.51,1.92)	**7.13 (1.23,41.34)**	1.50 (0.54,4.14)	1.05 (0.60,1.87)
1.36 (0.60,3.06)	1.17 (0.35,3.94)	1.11 (0.61,2.04)	**Ocrelizumab**	1.00 (0.44,2.30)	1.04 (0.02,58.42)	1.47 (0.42,5.14)	1.09 (0.48,2.49)	1.11 (0.45,2.71)	**7.94 (1.24,50.92)**	1.67 (0.51,5.44)	1.17 (0.51,2.69)
1.35 (0.61,2.98)	1.17 (0.35,3.87)	1.11 (0.63,1.97)	1.00 (0.43,2.29)	**Natalizumab**	1.04 (0.02,57.99)	1.47 (0.43,5.05)	1.09 (0.49,2.43)	1.11 (0.46,2.64)	**7.92 (1.25,50.26)**	1.66 (0.52,5.34)	1.17 (0.52,2.62)
1.30 (0.02,72.20)	1.12 (0.02,68.87)	1.07 (0.02,57.14)	0.96 (0.02,53.69)	0.96 (0.02,53.56)	Methotrexate **	1.41 (0.02,87.48)	1.05 (0.02,58.41)	1.06 (0.02,59.99)	7.61 (0.10,590.11)	1.60 (0.03,97.14)	1.13 (0.02,62.74)
0.92 (0.27,3.14)	0.80 (0.17,3.64)	0.76 (0.25,2.27)	0.68 (0.19,2.38)	0.68 (0.20,2.35)	0.71 (0.01,44.05)	**Laquinimod**	0.74 (0.22,2.55)	0.75 (0.21,2.71)	5.40 (0.68,42.89)	1.13 (0.25,5.06)	0.80 (0.23,2.75)
1.24 (0.57,2.71)	1.07 (0.32,3.52)	1.02 (0.58,1.78)	0.91 (0.40,2.08)	0.92 (0.41,2.04)	0.95 (0.02,53.04)	1.34 (0.39,4.60)	**Interferon beta‐1b**	1.01 (0.43,2.40)	**7.25 (1.15,45.89)**	1.52 (0.48,4.87)	1.07 (0.48,2.39)
1.22 (0.52,2.88)	1.06 (0.31,3.66)	1.01 (0.52,1.94)	0.90 (0.37,2.21)	0.90 (0.38,2.16)	0.94 (0.02,53.19)	1.33 (0.37,4.77)	0.99 (0.42,2.35)	**Interferon beta‐1a**	**7.17 (1.10,46.83)**	1.50 (0.45,5.06)	1.06 (0.44,2.53)
0.17 (0.03,1.08)	0.15 (0.02,1.16)	**0.14 (0.02,0.81)**	**0.13 (0.02,0.81)**	**0.13 (0.02,0.80)**	0.13 (0.00,10.19)	0.19 (0.02,1.47)	**0.14 (0.02,0.87)**	**0.14 (0.02,0.91)**	**Immunoglobulins**	0.21 (0.03,1.60)	**0.15 (0.02,0.94)**
0.81 (0.26,2.59)	0.70 (0.18,2.79)	0.67 (0.24,1.85)	0.60 (0.18,1.96)	0.60 (0.19,1.94)	0.63 (0.01,38.11)	0.88 (0.20,3.95)	0.66 (0.21,2.10)	0.67 (0.20,2.24)	4.77 (0.62,36.43)	**Glatiramer acetate**	0.71 (0.22,2.27)
1.15 (0.52,2.55)	1.00 (0.30,3.30)	0.95 (0.54,1.68)	0.85 (0.37,1.95)	0.85 (0.38,1.91)	0.89 (0.02,49.52)	1.25 (0.36,4.31)	0.93 (0.42,2.08)	0.94 (0.39,2.26)	**6.76 (1.06,42.93)**	1.42 (0.44,4.56)	**Fingolimod**

*Significant results are bolded and underlined.  **Network meta‐analysis estimates including only available comparisons vs placebo (common comparator) are reported. The only available study on methotrexate vs placebo, Goodkin 1995, reported zero events in both groups relative to serious adverse events. Network meta‐analysis was performed by means of STATA. In order to retain methotrexate in the network for indirect comparisons, a value of 0.5 events was imputed. In Analysis 2.1 (pair‐wise meta‐analysis), the pairwise odds ratio was calculated using RevMan, allowing only the value of zero events. Therefore, the forest plot reports zero events and the 'not estimable' warning.

**6:** Netleague: Treatment discontinuation due to adverse events*

**Siponimod**	2.62 (0.52,13.29)	0.65 (0.42,1.02)	0.81 (0.32,2.08)	0.67 (0.31,1.43)	2.45 (0.51,11.83)	**1.95 (1.05,3.62)**	1.92 (0.92,3.98)	1.28 (0.57,2.86)	2.60 (0.88,7.70)	1.50 (0.80,2.82)	5.54 (0.27,115.42)
0.38 (0.08,1.94)	**Rituximab**	0.25 (0.05,1.19)	0.31 (0.05,1.82)	0.26 (0.05,1.38)	0.94 (0.11,8.24)	0.74 (0.15,3.77)	0.73 (0.14,3.89)	0.49 (0.09,2.68)	0.99 (0.21,4.64)	0.57 (0.11,2.92)	2.12 (0.07,62.62)
1.53 (0.98,2.38)	4.00 (0.84,19.12)	**Placebo/no treatment**	1.24 (0.54,2.86)	1.02 (0.55,1.90)	3.75 (0.83,16.99)	**2.98 (1.92,4.61)**	**2.93 (1.64,5.26)**	1.95 (0.99,3.84)	**3.98 (1.48,10.72)**	**2.29 (1.46,3.60)**	8.47 (0.42,170.95)
1.23 (0.48,3.17)	3.23 (0.55,19.02)	0.81 (0.35,1.86)	**Ocrelizumab**	0.83 (0.29,2.34)	3.02 (0.54,17.01)	2.40 (0.94,6.17)	2.37 (0.85,6.55)	1.58 (0.54,4.62)	3.21 (0.88,11.74)	1.85 (0.72,4.78)	6.84 (0.30,154.58)
1.49 (0.70,3.20)	3.91 (0.73,21.02)	0.98 (0.53,1.81)	1.21 (0.43,3.42)	**Natalizumab**	3.66 (0.71,18.75)	**2.91 (1.36,6.21)**	**2.86 (1.22,6.71**)	1.91 (0.76,4.77)	**3.89 (1.21,12.51)**	**2.24 (1.04,4.82)**	8.28 (0.39,177.86)
0.41 (0.08,1.97)	1.07 (0.12,9.41)	0.27 (0.06,1.21)	0.33 (0.06,1.86)	0.27 (0.05,1.40)	**Laquinimod**	0.79 (0.16,3.83)	0.78 (0.15,3.96)	0.52 (0.10,2.73)	1.06 (0.17,6.48)	0.61 (0.13,2.97)	2.26 (0.08,65.34)
**0.51 (0.28,0.96)**	1.34 (0.27,6.82)	**0.34 (0.22,0.52)**	0.42 (0.16,1.07)	**0.34 (0.16,0.73)**	1.26 (0.26,6.07)	**Interferon beta‐1b**	0.98 (0.47,2.04)	0.66 (0.29,1.47)	1.34 (0.45,3.95)	0.77 (0.41,1.44)	2.85 (0.14,59.27)
0.52 (0.25,1.08)	1.37 (0.26,7.25)	**0.34 (0.19,0.61)**	0.42 (0.15,1.17)	**0.35 (0.15,0.82)**	1.28 (0.25,6.46)	1.02 (0.49,2.11)	**Interferon beta‐1a**	0.67 (0.27,1.63)	1.36 (0.43,4.29)	0.78 (0.37,1.64)	2.89 (0.14,61.68)
0.78 (0.35,1.76)	2.05 (0.37,11.27)	0.51 (0.26,1.01)	0.63 (0.22,1.86)	0.52 (0.21,1.31)	1.92 (0.37,10.06)	1.53 (0.68,3.42)	1.50 (0.61,3.67)	**Immunoglobulins**	2.04 (0.61,6.77)	1.18 (0.52,2.65)	4.34 (0.20,94.43)
0.38 (0.13,1.14)	1.01 (0.22,4.70)	0.25 (0.09,0.68)	0.31 (0.09,1.14)	**0.26 (0.08,0.83)**	0.94 (0.15,5.74)	0.75 (0.25,2.21)	0.74 (0.23,2.33)	0.49 (0.15,1.63)	**Glatiramer acetate**	0.58 (0.19,1.71)	2.13 (0.09,50.37)
0.67 (0.36,1.25)	1.74 (0.34,8.88)	**0.44 (0.28,0.68)**	0.54 (0.21,1.39)	**0.45 (0.21,0.96)**	1.63 (0.34,7.91)	1.30 (0.69,2.43)	1.28 (0.61,2.67)	0.85 (0.38,1.92)	1.74 (0.58,5.15)	**Fingolimod**	3.69 (0.18,77.07)
0.18 (0.01,3.76)	0.47 (0.02,13.97)	0.12 (0.01,2.38)	0.15 (0.01,3.31)	0.12 (0.01,2.60)	0.44 (0.02,12.77)	0.35 (0.02,7.32)	0.35 (0.02,7.38)	0.23 (0.01,5.01)	0.47 (0.02,11.11)	0.27 (0.01,5.65)	**Azathioprine**

*Significant results are bolded and underlined.

Estimates of safety secondary outcomes of each treatment against placebo and against each other treatment included in the network are shown in Appendix 5.

#### 1. Primary outcomes

##### 1.1 Efficacy

###### Relapses over 12, 24, and 36 months and disability worsening over 24 and 36 months

####### Pair‐wise meta‐analysis (direct comparisons)

Treatment estimates for pair‐wise meta‐analyses are reported in Analysis 1.1; Analysis 1.2; Analysis 1.3; Analysis 1.4; Analysis 1.5.

####### Network meta‐analysis estimates (combination of direct and indirect comparisons) of treatment effects

See: Table 1; Table 2; Table 3; Table 4; Table 5.

a) Relapses over 12 months were reported in one study involving 318 participants with PMS (3.13% of the participants in this review) (Hommes 2004), and assessing one treatment. The network geometry for relapses over 12 months is shown in Figure 4. Compared to placebo, relapse rate may be trivially increased with immunoglobulins (risk ratio (RR) 1.04, 95% confidence interval (CI) 0.76 to 1.41; very low certainty evidence), but the evidence is very uncertain.

b) Relapses over 24 months were reported in six studies involving 1622 participants with PMS (16% of the participants in this review) (Goodkin 1995; Hawker 2009; Hommes 2004; IMPACT 2002; Pohlau 2007; PROMESS 2017), and assessing six treatments. The network geometry for relapses over 24 months is shown in Figure 4.

Four treatments, assessed in five studies, were compared to placebo. For two treatments, evaluated in one study, we found only head‐to‐head comparisons. Using placebo as the common comparator, interferon beta‐1a may moderately reduce relapse rate (RR 0.72, 95% CI 0.54 to 0.95; very low certainty evidence), but the evidence is very uncertain (see Figure 6). Rituximab probably results in a trivial reduction of relapse rate (RR 0.60, 95% CI 0.19 to 1.95; moderate certainty evidence). There may be a trivial increase in relapse rate with methotrexate (RR 1.12, 95% CI 0.38 to 3.28; very low certainty evidence) and a trivial reduction with immunoglobulins (RR 0.96, 95% CI 0.79 to 1.16; very low certainty evidence), but the evidence is very uncertain. Differences across treatments were found and are shown in Table 8.

c) Relapses over 36 months were reported in four studies involving 2095 participants with PMS (21% of the participants in this review) (Anderson 2004; Ellison 1989; European Study Group 1998; NASP 2004), and assessing three treatments. The network geometry for relapses over 36 months is shown in Figure 4.

Three treatments, assessed in four studies, were compared to placebo. Interferon beta‐1b probably results in a trivial reduction in relapse rate (RR 0.82, 95% CI 0.73 to 0.93; moderate certainty evidence). Interferon beta‐1a may be associated with a trivial increase in relapse rate (RR 1.03, 95% CI 0.79 to 1.34; very low certainty evidence), but the evidence is very uncertain. Azathioprine may result in a large reduction in relapse rate (RR 0.54, 95% CI 0.30 to 0.99; very low certainty evidence), but the evidence is very uncertain (see Figure 6). Differences across treatments were found and are shown in Table 9.

d) Disability worsening over 24 months was reported in 11 studies involving 5284 participants with PMS (52% of the participants in this review) (ASCEND 2018; Bornstein 1991; EXPAND 2018; Goodkin 1995; Hawker 2009; Hommes 2004; IMPACT 2002; Montalban 2009; Pohlau 2007; PROMESS 2017; Wolinsky 2007), and assessing 10 treatments. The network geometry for disability over 24 months is shown in Figure 4.

Eight treatments, assessed in 10 studies, were compared to placebo. For two treatments, evaluated in one study, we had only head‐to‐head comparison.

Compared to placebo, methotrexate may result in a large reduction in disability at 24 months (RR 0.69, 95% CI 0.34 to 1.37; very low certainty evidence), but the evidence is very uncertain (see Figure 6). There may be a moderate reduction in disability at 24 months with glatiramer acetate (RR 0.84, 95% CI 0.59 to 1.20; very low certainty evidence), interferon beta‐1b (RR 0.69, 95% CI 0.29 to 1.60; very low certainty evidence), siponimod (RR 0.77, 95% CI 0.52 to 1.16; very low certainty evidence), and rituximab (RR 0.78, 95% CI 0.50 to 1.21; very low certainty evidence), but the evidence is very uncertain for all these interventions. There may be a small reduction in disability at 24 months with immunoglobulins (RR 0.92, 95% CI 0.68 to 1.25; very low certainty evidence), interferon beta‐1a (RR 0.85, 95% CI 0.54 to 1.33; very low certainty evidence), and natalizumab (RR 0.83, 95% CI 0.55 to 1.27; very low certainty evidence), but the evidence is very uncertain. Differences across treatments were found and are shown in Table 10.

e) Disability worsening over 36 months was reported in five studies involving 2827 participants with PMS (28% of the participants in this review) (Anderson 2004; Ellison 1989; European Study Group 1998; NASP 2004; ORATORIO 2017), and assessing four treatments.

Four treatments, assessed in five studies, were compared to placebo.

Compared to placebo, azathioprine may result in a large reduction in disability at 36 months (RR 0.63, 95% CI 0.28 to 1.44; very low certainty evidence), but the evidence is very uncertain (see Figure 6). Ocrelizumab may result in a moderate reduction in disability at 36 months (RR 0.83, 95% CI 0.55 to 1.25; very low certainty evidence), but the evidence is very uncertain. Interferon beta‐1b may result in a small reduction in disability at 36 months (RR 0.90, 95% CI 0.68 to 1.18; very low certainty evidence), but the evidence is very uncertain. Interferon beta‐1a may be associated with a small increase in disability at 36 months (RR 1.10, 95% CI 0.72 to 1.70; very low certainty evidence), but the evidence is very uncertain. Differences across treatments were found and are shown in Table 11.

##### 1.2 Safety

###### Serious adverse events (SAEs) and treatment discontinuation due to adverse events (AEs)

####### Pair‐wise meta‐analysis (direct comparisons)

Treatment estimates for pair‐wise meta‐analyses are reported in Analysis 2.1; Analysis 2.2.

####### Network meta‐analysis estimates (combination of direct and indirect comparisons) of treatment effects

See: Table 6; Table 7.

a) SAEs were reported in 15 studies involving 8019 participants with PMS (79% of the participants in this review) (Anderson 2004; ARPEGGIO 2020; ASCEND 2018; Cheshmavar 2020; EXPAND 2018; Goodkin 1995; Hawker 2009; Hommes 2004; INFORMS 2016; Komori 2016; NASP 2004; ORATORIO 2017; Pohlau 2007; PROMESS 2017; Wolinsky 2007), and assessing 13 treatments. The network geometry for treatment discontinuation due to AEs is shown in Figure 4.

Eleven treatments, assessed in 13 studies, were compared to placebo. For two treatments, evaluated in one study, we had only head‐to‐head comparison. Using placebo as the common comparator (see Figure 6), treatment with interferon beta‐1b may result in a trivial reduction in SAEs (odds ratio (OR) 0.98, 95% CI 0.56 to 1.72; low certainty evidence). There may be a trivial reduction in SAEs with interferon beta‐1a (OR 0.99, 95% CI 0.51 to 1.92; very low certainty evidence), methotrexate (OR 0.94, 95% CI 0.02 to 50.12; very low certainty evidence), and natalizumab (OR 0.90, 95% CI 0.51 to 1.59; very low certainty evidence), but the evidence is very uncertain for all these interventions. Treatment with glatiramer acetate may result in a trivial increase in participants with SAEs (OR 1.50, 95% CI 0.54 to 4.14; low certainty evidence). The following interventions may result in a trivial increase in participants with SAEs, but the evidence is very uncertain: immunoglobulins (OR 7.13, 95% CI 1.23 to 41.34; very low certainty evidence), rituximab (OR 1.05, 95% CI 0.37 to 3.01; very low certainty evidence), siponimod (OR 1.22, 95% CI 0.70 to 2.10; very low certainty evidence), fingolimod (OR 1.05, 95% CI 0.60 to 1.87; very low certainty evidence), ocrelizumab (OR 0.90, 95% CI 0.49 to 1.64; very low certainty evidence), laquinimod (OR 1.32, 95% CI 0.44 to 3.95; very low certainty evidence). Differences across treatments were found and are shown in Table 12.

b) Treatment discontinuation due to AEs was reported in 21 studies involving 9981 participants with PMS (98.2% of the participants in this review) (Anderson 2004; ARPEGGIO 2020; ASCEND 2018; Cheshmavar 2020; Ellison 1989; European Study Group 1998; EXPAND 2018; Goodkin 1995; Hawker 2009; Hommes 2004; IMPACT 2002; INFORMS 2016; Komori 2016; Leary 2003; Montalban 2009; NASP 2004; ORATORIO 2017; Pohlau 2007; PROMESS 2017; SPECTRIMS 2001; Wolinsky 2007), and assessing 14 treatments. The network geometry for treatment discontinuation due to AEs is shown in Figure 4.

Eleven treatments, assessed in 17 studies, were compared to placebo. For two treatments, evaluated in one study, we had only head‐to‐head comparison. Using placebo as the common comparator, treatment with interferon beta‐1a results in a trivial increase in the number of participants who discontinued due to AEs (OR 2.93, 95% CI 1.64 to 5.26; high certainty evidence) (see Figure 6). The following interventions probably result in a trivial increase in the number of participants who discontinued due to AEs: rituximab (OR 4.00, 95% CI 0.84 to 19.12; moderate certainty evidence), interferon beta‐1b (OR 2.98, 95% CI 1.92 to 4.61; moderate certainty evidence), immunoglobulins (OR 1.95, 95% CI 0.99 to 3.84; moderate certainty evidence), glatiramer acetate (OR 3.98, 95% CI 1.48 to 10.72; moderate certainty evidence), natalizumab (OR 1.02, 95% CI 0.55 to 1.90; moderate certainty evidence), siponimod (OR 1.53, 95% CI 0.98 to 2.38; moderate certainty evidence), fingolimod (OR 2.29, 95% CI 1.46 to 3.60; moderate certainty evidence), ocrelizumab (OR 1.24, 95% CI 0.54 to 2.86; moderate certainty evidence). Treatment with laquinimod may result in a trivial increase in the number of participants who discontinued due to AEs (OR 3.75, 95% CI 0.83 to 16.99; low certainty evidence). Treatment with azathioprine may result in a trivial increase in the number of participants who discontinued due to AEs (OR 8.47, 95% CI 0.42 to 170.95; very low certainty evidence), but the evidence is very uncertain. Differences across treatments were found and are shown in Table 13.

Two studies (Goodkin 1995 on methotrexate and Montalban 2009 on interferon beta‐1b) with 0 events in both arms were excluded from the analyses.

#### 2. Secondary outcomes

##### 2.1 Efficacy

###### Cognitive decline; quality of life impairment; new or enlarging T2‐weighted MRI lesions and new gadolinium‐enhancing positive T1‐weighted MRI lesions at 12, 24, and 36 months

####### Pair‐wise meta‐analysis (direct comparisons)

Treatment estimates for pair‐wise meta‐analyses for each outcome are reported in Analysis 3.1; Analysis 3.2; Analysis 3.3; Analysis 3.4; Analysis 3.5; Analysis 3.6; Analysis 3.7; Analysis 3.8; Analysis 3.9.

####### Network meta‐analysis estimates (combination of direct and indirect comparisons) of treatment effects

The network geometry for each outcome is presented in Figure 5.

a) New gadolinium‐enhancing positive T1‐weighted MRI lesions at 12 months was reported in two studies involving 520 participants with PMS (5% of those included in this review) (Cheshmavar 2020; IMPACT 2002), and assessing three treatments.

One treatment assessed in one study was compared to placebo. For two treatments, evaluated in one study, we had only head‐to‐head comparison. Using placebo as the common comparator, treatment with interferon beta‐1a (Avonex, Rebif) probably results in a moderate reduction in new gadolinium‐enhancing positive T1‐weighted MRI lesions (RR 0.40, 95% CI 0.26 to 0.61).

b) New gadolinium‐enhancing positive T1‐weighted MRI lesions at 24 months was reported in two studies involving 2087 participants with PMS (21% of those included in this review) (EXPAND 2018; IMPACT 2002), and assessing two treatments.

Two treatments assessed in two studies were compared to placebo. Treatment with siponimod (RR 0.32, 95% CI 0.26 to 0.40) results in a moderate reduction in new gadolinium‐enhancing positive T1‐weighted MRI lesions. Treatment with interferon beta‐1a (Avonex, Rebif) (RR 0.46, 95% CI 0.29 to 0.71) probably results in a moderate reduction in new gadolinium‐enhancing positive T1‐weighted MRI lesions.

c) New gadolinium‐enhancing positive T1‐weighted MRI lesions at 36 months was reported in one study involving 823 participants with PMS (8% of those included in this review) (INFORMS 2016), and comparing fingolimod with placebo.

Treatment with fingolimod probably results in a small reduction in new gadolinium‐enhancing positive T1‐weighted MRI lesions (RR 0.58, 95% CI 0.41 to 0.82).

d) New or enlarging T2‐weighted MRI lesions at 12 months was reported in one study involving 436 participants with PMS (4% of those included in this review) (IMPACT 2002), and comparing interferon beta‐1a (Avonex, Rebif) with placebo.

Treatment with interferon beta‐1a (Avonex, Rebif) probably results in a moderate reduction in new or enlarging T2‐weighted MRI lesions (RR 0.53, 95% CI 0.39 to 0.73).

e) New or enlarging T2‐weighted MRI lesions at 24 months was reported in two studies involving 2087 participants with PMS (21% of those included in this review) (EXPAND 2018; IMPACT 2002), and comparing two treatments with placebo.

Treatment with siponimod (RR 0.68, 95% CI 0.62 to 0.75) results in a moderate reduction in new or enlarging T2‐weighted MRI lesions. Treatment with interferon beta‐1a (Avonex, Rebif) (RR 0.62, 95% CI 0.49 to 0.80) may result in a moderate reduction in new or enlarging T2‐weighted MRI lesions.

f) New or enlarging T2‐weighted MRI at 36 months was reported in one study involving 823 participants with PMS (8% of those included in this review) (INFORMS 2016), and comparing fingolimod with placebo.

Treatment with fingolimod probably results in a moderate reduction in new or enlarging T2‐weighted MRI lesions (RR 0.51, 95% CI 0.39 to 0.66).

g) No studies assessed cognitive decline.

h) Data for quality of life impairment were reported with different scales (non‐MS‐related quality of life questionnaires and MS‐related questionnaires) and subscales (physical and mental).

Quality of life impairment (MS related; total, measured with the MSIS‐29) was reported in one study involving 889 participants with PMS (9% of those included in this review) (ASCEND 2018), and comparing natalizumab with placebo.

Treatment with natalizumab probably results in a trivial increase in quality of life (mean difference (MD) 2.73, 95% CI 0.05 to 5.41) at 24 months (Analysis 3.7).

Quality of life impairment (non‐MS related; mental subscale of the SF‐36) was reported in one study involving 436 participants with PMS (4% of those included in this review) (IMPACT 2002), and comparing interferon beta‐1a with placebo.

Treatment with interferon beta‐1a (Avonex, Rebif) probably results in a trivial increase in quality of life (non‐MS related; mental subscale of the SF‐36) at 24 months (MD 1.99, 95% CI 0.22 to 3.76) (Analysis 3.8).

Quality of life impairment (non‐MS related; physical subscale of the SF‐36) was reported in two studies involving 1168 participants with PMS (11.5% of those included in this review) (IMPACT 2002; ORATORIO 2017), and comparing interferon beta‐1a and ocrelizumab with placebo.

Treatment with interferon beta‐1a may result in a trivial increase in quality of life at 24 months (non‐MS related; physical subscale of the SF‐36) (standardised mean difference (SMD) 0.10 standard deviation (SD), 95% CI −0.09 to 0.28), while treatment with ocrelizumab probably results in a trivial increase in quality of life (non‐MS related; physical subscale of the SF‐36) (SMD 0.04 SD, 95% CI −0.12 to 0.19) at 30 months (Analysis 3.9).

##### 2.2 Safety

###### Mortality

####### Pair‐wise meta‐analysis (direct comparisons)

Treatment estimates for pair‐wise meta‐analyses are reported in Analysis 3.10.

####### Network meta‐analysis estimates (combination of direct and indirect comparisons) of treatment effects

The network geometry is presented in Figure 5, and Appendix 5 shows the network estimates of each treatment against placebo or against another treatment within the network.

a) Mortality was reported in 21 studies involving 9316 participants with PMS (92% of those included in this review) (Anderson 2004; ARPEGGIO 2020; ASCEND 2018; Bornstein 1991; Cheshmavar 2020; Ellison 1989; European Study Group 1998; EXPAND 2018; Goodkin 1995; Hawker 2009; Hommes 2004; IMPACT 2002; INFORMS 2016; Komori 2016; Leary 2003; Montalban 2009; NASP 2004; ORATORIO 2017; Pohlau 2007; SPECTRIMS 2001; Wolinsky 2007), and assessing 11 treatments.

Eleven treatments assessed in 15 studies were compared to placebo. Treatment with glatiramer acetate probably results in a small reduction in number of deaths (OR 0.28, 95% CI 0.08 to 0.98).

Treatment with immunoglobulins may result in a trivial reduction in number of deaths (OR 0.33, 95% CI 0.01 to 8.20). Treatment with siponimod probably results in a trivial reduction in number of deaths (OR 0.49, 95% CI 0.12 to 1.98). Treatment with immunoglobulins may result in a trivial reduction in number of deaths (OR 0.25, 95% CI 0.02 to 2.78). Treatment with azathioprine may result in a small reduction in number of deaths (OR 0.50, 95% CI 0.04 to 5.79). Treatment with fingolimod may result in a trivial reduction in number of deaths (OR 0.72, 95% CI 0.07 to 8.02). Treatment with interferon beta‐1b may result in a trivial increase in number of deaths (OR 2.28, 95% CI 0.58 to 9.03). Treatment with interferon beta‐1a may result in a trivial increase in number of deaths (OR 1.55, 95% CI 0.41 to 5.91). Treatment with ocrelizumab may result in a trivial increase in number of deaths (OR 2.01, 95% CI 0.22 to 18.07). Treatment with laquinimod may result in a trivial increase in number of deaths (OR 1.81, 95% CI 0.07 to 44.62). Treatment with natalizumab may result in a trivial increase in number of deaths (OR 3.07, 95% CI 0.12 to 75.52).

Six studies (Bornstein 1991 on glatiramer acetate; Cheshmavar 2020 on rituximab and glatiramer acetate; Goodkin 1995 on methotrexate; Hommes 2004 on immunoglobulins; Komori 2016 on rituximab; and Leary 2003 on interferon beta‐1a (Avonex, Rebif)) with 0 events in both arms were excluded from the analyses. Relative treatment rankings (SUCRA and mean rank) for each primary and secondary outcome are presented in Appendix 6. Given the few number of studies for each comparison and the large number of treatments, these results should be interpreted with caution.

#### 3. Assessment of heterogeneity and incoherence within the network analyses

We performed an assessment of heterogeneity and incoherence within the network analyses for all analyses whenever possible. The values for common heterogeneity (Tau^2^) for the network for each outcome appear to show no evidence of heterogeneity (Appendix 7). Assessment of incoherence was possible for SAE and treatment discontinuation due to AE. We did not observe evidence of local and global incoherence (Appendix 8).

#### 4. Subgroup and sensitivity analyses

##### Subgroup analysis

We did not perform subgroup analysis for type of PMS because all the studies included participants with active PMS.

##### Sensitivity analysis

We did not perform sensitivity analysis including only studies at low risk of selection bias (allocation concealment) for the outcomes disability at 24 months and disability and relapses at 36 months because a few studies were at low risk of bias, and they provided the same evidence as the overall analysis; for relapses at 12 months, because only one study was included; and for relapses at 24 months because all studies but one were at high risk of bias.

We did not perform sensitivity analysis including only studies at low risk of attrition bias because all studies considering primary efficacy outcomes were at low risk for this domain.

We did not perform sensitivity analysis excluding studies with a sample size smaller than 50 randomised participants because only one study satisfied this criterion.

#### 5. Reporting bias

We did not produce a contour‐enhanced funnel plot for each pair‐wise comparison due to the low number of studies. We employed a comparison‐adjusted funnel plot for all placebo‐controlled trials for disability at 24 months, treatment discontinuation due to AEs, and SAEs, and small‐study effects (not necessarily due to reporting bias) did not appear to be present (data not shown).

## Discussion

### Summary of main results

This review of the effects of treatments for PMS included 23 studies involving 10,167 adult participants. Twenty studies (87%) were placebo‐controlled, and three studies (13%) were head‐to‐head comparisons. The median RCT duration was 24 months; 60% of studies were short‐term trials with follow‐up of 12 or 24 months, while the remaining studies had follow‐up duration ranging from 27 to 60 months, therefore the long‐term effects of these treatments remain uncertain.

#### 1. Recurrence of relapses

Using placebo as the common comparator, only one study on immunoglobulins assessed relapses at 12 months: relapses at 12 months may be trivially increased with immunoglobulins, but the evidence is very uncertain. Six studies provided data at 24 months follow‐up on four different treatments: relapses at 24 months were probably trivially reduced with rituximab, while the evidence is very uncertain whether relapses are reduced by interferon beta‐1a, trivially reduced with immunoglobulins, and trivially increased with methotrexate. Four studies provided data at 36 months follow‐up on three treatments: relapses at 36 months are probably trivially reduced with interferon beta‐1b, while they may be reduced with azathioprine and trivially reduced with interferon beta‐1a, but the evidence is very uncertain.

#### 2. Disability worsening

Using placebo as the common comparator, 11 studies on eight treatments provided data at 24 months follow‐up. Regarding numbers of people who experience disability worsening at 24 months, there may be a large reduction with methotrexate; a moderate reduction with glatiramer acetate, interferon beta‐1b, siponimod, and rituximab; and a small reduction with immunoglobulins, interferon beta‐1a, and natalizumab, but the evidence for all these interventions is very uncertain. Five studies on four treatments provided data at 36 months follow‐up. Regarding numbers of people who experience disability worsening at 36 months, there may be a large reduction with azathioprine; a moderate reduction with ocrelizumab; a small reduction with interferon beta‐1b; and a small increase with interferon beta‐1a, but the evidence for all these interventions is very uncertain.

#### 3. Safety

Using placebo as the common comparator, 13 studies on 11 treatments provided data on SAEs. The numbers of people who experience one or more SAEs may be trivially reduced with interferon beta‐1b and trivially increased with glatiramer acetate; may be trivially reduced with interferon beta‐1a, methotrexate, and natalizumab, but the evidence is very uncertain; and may be trivially increased with immunoglobulins, rituximab, siponimod, fingolimod, ocrelizumab, and laquinimod, but the evidence is very uncertain.

Using placebo as the common comparator, 17 studies on 11 treatments provided data on treatment discontinuation due to AEs. The number of people who discontinued treatment due to AEs during the trial is trivially increased with interferon beta‐1a; is probably trivially increased with rituximab, interferon beta‐1b, immunoglobulins, glatiramer acetate, natalizumab, siponimod, fingolimod, and ocrelizumab; may be trivially increased with laquinimod; and may be trivially increased with azathioprine, but the evidence is very uncertain.

### Overall completeness and applicability of evidence

Nine critical outcomes were identified by the Multiple Sclerosis International Federation (MSIF) Essential Medicines Panel (MEMP). These informed the current review, but the outcomes were further differentiated into primary and secondary outcomes and assessed solely for certainty and efficacy/harm, in line with standard Cochrane methodology. The data underlying the review, from all nine outcomes, served as the evidence base for the MEMP guideline panel, where it was contextualised with the perspective of low‐resource settings by considering further evidence related to other domains, in line with GRADE Evidence to Decision Framework methodology (Alonso‐Coello 2016a; Alonso‐Coello 2016b). The MEMP recommendations were used as the basis of an application for the inclusion of DMTs in the 23rd WHO Model List of Essential Medicines.

The majority of the evidence relating to new relapses, disability worsening, and adverse events that was included in this review was collected at 12 and 24 months follow‐up, with only four studies reporting at 36 months. MS is a chronic condition with a duration of 30 to 40 years. As such, the duration of the available evidence on efficacy and safety limits its applicability, especially for long‐term and uncommon AEs.

We identified evidence for 15 treatments from 23 studies, involving 10,167 adult participants, with all but three studies with 302 participants (3% of total) involving comparisons with placebo as opposed to head‐to‐head comparisons with other DMTs. It is therefore unclear if the results of the review fit into the context of current practice, since about 50% of people with MS are treated with at least one DMT (Carroll 2014).

The reasons why the 23 available randomised studies for PMS were mostly placebo‐controlled, and outcome data reported mainly over 24 months, are likely: i) comparison with placebo in one double‐blind, superiority RCT is sufficient for approval of DMTs for PMS by many national regulatory agencies; ii) the lack of interest of pharmaceutical companies in conducting longer expensive studies, given that only recently have some regulatory agencies recommended a duration of three years for confirmatory trials (EMA 2015); iii) the unlikely advantage of pharmaceutical companies in conducting head‐to‐head trials directly comparing active treatments.

Finally, it should be noted that certain drugs included in our review are not on‐label for treatment of progressive MS in different jurisdictions, which could impact the global applicability of our results.

### Quality of the evidence

Considering risk of bias, the most frequent concern was related to the role of the sponsor in the authorship of the study report or in data management and analysis, for which we judged 39% of the studies at high risk of bias. Other frequent concerns were performance, attrition, and selective reporting bias, with 8.7% of the studies at high risk of bias for all three of these domains.

We downgraded the certainty of evidence only for imprecision, across all the outcomes and comparisons. We assessed the certainty of the evidence for the outcomes relapses, disability, and SAEs as very low for most treatments, given that the CIs crossed several thresholds, according to the contextualised approach. We assessed the certainty of the evidence for the outcome discontinuation due to AEs as moderate for most treatments considered.

Across outcomes, rituximab and interferon beta‐1b had the highest certainty of evidence, except for treatment discontinuation due to AEs, for which interferon beta‐1a had the highest certainty.

### Potential biases in the review process

#### 1. Transitivity assumption

We assumed that any patient who met the inclusion criteria was, in principle, equally likely to have been randomised to any of the eligible interventions. We evaluated the assumption of transitivity by assessing differences in patient characteristics such as age, disease duration, and baseline Expanded Disability Status Scale (EDSS) scores across the trials, and by comparing the predefined potential effect modifiers across the different comparisons in the networks. We did not find any evidence that important variables varied across comparisons or altered the effectiveness of the treatments; although some confounders may be hidden and unmeasured, it might be reasonable to analyse the network as a whole. We thus assumed that the transitivity held, and a network meta‐analytical approach was reasonable. However, few studies per comparison were available, and limitations in study reporting cannot exclude the possibility of intransitivity.

#### 2. Heterogeneity and incoherence

We did not find any strong evidence of the presence of heterogeneity either in direct pair‐wise comparisons or in the entire networks. Similarly, the loop‐specific approach, node‐splitting approach, and the 'design‐by‐treatment' model did not provide any clear indication of the presence of incoherence either locally or in the entire networks. We thus believe that the coherence assumption is reasonable for this type of data. However, the power of these tests and approaches to detect incoherence is low, particularly for networks with few included studies per comparison.

#### 3. Subgroup and sensitivity analyses

We did not perform subgroup analysis for type of PMS because all studies included people with active PMS, showing that our studies were homogenous in terms of type of PMS.

#### 4. Reporting bias

The comparison‐adjusted funnel plot for comparisons versus placebo conducted for disability at 24 months, discontinuation due to AEs, and SAEs did now show possible presence of reporting bias.

#### 5. Certainty of the evidence

As reported in Summary of findings and assessment of the certainty of the evidence, the certainty of the evidence for this review was assessed using a fully contextualised approach, involving the definition of quantitative thresholds to determine the magnitude ('trivial', 'small', 'moderate', and 'large') of each health effect measured by each outcome. Quantitative thresholds between magnitudes of health effects were considered when assessing imprecision, one of the domains contributing to the certainty of the evidence. Thresholds were calculated from outcome‐specific numerical health state utility values (HSUVs). Whenever HSUVs were not obtainable from published evidence, they were set through panel judgement, thereby reflecting the panel members' potentially biased views and expectations.

### Agreements and disagreements with other studies or reviews

A previous Cochrane review with NMA investigated the efficacy and safety of DMTs in people with relapsing‐remitting MS (RRMS) and with PMS (Filippini 2013). All 15 studies considered in that review and eight additional studies published afterwards were included in our review, providing evidence on six DMTs (laquinimod, natalizumab, rituximab, siponimod, fingolimod, and ocrelizumab) in people with PMS that had not been considered in Filippini 2013. Unlike the review by Filippini and colleagues, which found no difference in terms of relapse frequency for any of the considered DMTs, we found evidence that rituximab at two years and interferon beta‐1b after three years of treatment probably reduce relapses in people with PMS. Ten years after the publication of Filippini 2013, our conclusions on disability progression remain similar, that is none of the considered DMTs is more effective than placebo over two to three years. Regarding safety, the previous Cochrane review found a higher rate of withdrawals due to AEs than placebo for all the considered DMTs, while we found this result for interferon beta‐1a, interferon beta‐1b, rituximab, immunoglobulins, glatiramer acetate, natalizumab, fingolimod, siponimod, and ocrelizumab. Filippini 2013 also found that SAEs were significantly more frequent among people treated with interferons than placebo, while we did not observe this difference in our review. However, making such comparisons between the two reviews is challenging, since for safety outcomes, Filippini and colleagues provided pooled estimates from studies on both RRMS and PMS.

A recent systematic review with NMA compared the efficacy on disability worsening at two years of three DMTs commonly used off‐label in PMS (rituximab, natalizumab, and fingolimod), and the two DMTs licenced for the treatment of primary progressive MS (PPMS) (ocrelizumab) and secondary progressive MS (SPMS) (siponimod) (Silva 2022). The literature search was from 1990 to December 2021. Five RCTs were included in the analysis, one for each of the five DMTs considered. All of them are also included in our NMA. Finding that between off‐label and licenced DMTs there is no significant difference compared to placebo, the authors concluded that any of the three DMTs without registered indications could be a fair, less expensive alternative to ocrelizumab and siponimod. Their main finding of a modest effect of all DMTs on disability progression, with no significant differences between licenced and off‐label treatments, may be considered to be in agreement with our results, that is showing little to no effect on disability worsening after 24 or 36 months of treatment. However, some major differences exist between that review and our review regarding the time frame of literature search and the choice of the interventions (both of which were much broader in our review), the type of comparisons, the type of populations (all RCTs with a mix of people with RRMS and PMS were not included in Silva 2022), and the quality assessment of the retrieved studies (limited to risk of bias without assessing the certainty in the estimates by means of the other domains according to the GRADE methodology), making it difficult to evaluate agreements and disagreements between the two reviews. Moreover, no safety outcomes were considered in the review by Silva and colleagues, a considerable limitation for an overall assessment of different treatment strategies, especially in relation to potential implications for practice.

In order to investigate the determinants of the modest effect of DMTs on disease progression in PMS, some authors postulated that immunomodulating treatments, instead of exerting a specific action on degenerative mechanisms typical of progressive phenotypes of MS, mostly target the inflammatory process causing relapses (Capanna 2022). Therefore, they systematically searched for RCTs on PMS providing clinical data at baseline that allowed the assumption of which people with MS had residual inflammatory activity. Pooled data from six RCTs on interferon beta‐1b, rituximab, fingolimod, ocrelizumab, and siponimod showed that the number of people with MS with confirmed disease progression was lower among those with residual inflammatory activity, therefore supporting the hypothesis that DMTs in PMS act mainly on inflammatory mechanisms, and their action in forms where degeneration prevails is less apparent. Although exploring an interesting hypothesis, such conclusions should be interpreted cautiously, since the analysis has the main limitation that all extracted data came from post hoc, hypothesis‐generating subgroup analyses of the included studies, which were not powered to demonstrate any difference.

## Authors' conclusions

**Implications for practice**

The results of this review should be interpreted with caution, since most of the included treatments have been evaluated in relatively few trials, and in many cases only one placebo‐controlled trial. Moreover, according to the GRADE approach, estimates of effect based on low to very low certainty evidence may be altered by future research, therefore implications for practice should be based mainly on evidence of moderate to high certainty.

Our results show that, when compared to placebo, rituximab after two years and interferon beta‐1b after three years of treatment probably slightly reduce relapses among people with progressive multiple sclerosis (PMS). However, both drugs are also associated with a slight increase in the proportion of people who withdraw due to adverse events; interferon beta‐1a, immunoglobulins, glatiramer acetate, natalizumab, fingolimod, siponimod, and ocrelizumab share this disadvantage. Unfortunately, we are uncertain about the effect of any treatment versus placebo on disability worsening at any time point or on the number of people with serious adverse events.

In making implications for practice, there are some important considerations. Firstly, few licenced disease‐modifying treatments (DMTs) are available for people with PMS, narrowing the range of choice among available medicines and the possibility of a patient‐tailored treatment. Secondly, the follow‐up duration (two to three years) of the available studies is relatively short, considering that the course of PMS is one that unfolds over decades; this is a limitation to the applicability of trial results in clinical practice. Thirdly, relatively short follow‐up time frames do not allow for the evaluation of severe and uncommon adverse events that may impact the clinical usefulness of a given DMT. Fourthly, the lack of head‐to‐head studies makes it challenging to evaluate the effectiveness and safety of each DMT relative to therapeutic alternatives. Finally, most studies included in this review were pivotal trials sponsored by pharmaceutical companies aimed at obtaining market licencing from regulatory agencies, which may have influenced their results.

**Implications for research**

Randomised trials with direct comparisons between active agents and with longer follow‐up (at least 36 months) are warranted in future research on DMTs for PMS. Given the relatively low incidence and prevalence of PMS, national and international registries and other types of non‐randomised studies on large populations might be additional valuable sources of data on the long‐term benefit and safety of DMTs for this condition.

Moreover, clinical research on PMS may benefit from long‐term data on outcomes deemed as relevant by people with MS, such as cognitive status and quality of life, as well as definition and validation of health state utility values.

Finally, the choice of outcomes and their assessment methods should be consistent across studies, particularly pivotal trials. Clinical events such as relapses and disability progression are commonly used in multiple sclerosis research as efficacy outcomes, but the heterogeneity in the way such outcomes are measured (e.g. mean Expanded Disability Status Scale (EDSS) scores rather than number of people with disability progression) makes it challenging to compare relative effectiveness and safety among DMTs.

## What's new

**Table:** 

**Date**	**Event**	**Description**
18 December 2024	Amended	Correction to Acknowledgements.

## History

Protocol first published: Issue 11, 2022 Review first published: Issue 9, 2024

**Table:** 

**Date**	**Event**	**Description**
10 November 2022	Amended	Publishing an amendment to correct author order error.

## Appendices

### Appendix 1. CENTRAL search strategy

**Table:**

#	Query
#1	MeSH descriptor: [Demyelinating Autoimmune Diseases, CNS] this term only
#2	MeSH descriptor: [Demyelinating Diseases] this term only
#3	MeSH descriptor: [Multiple Sclerosis] explode all trees
#4	MeSH descriptor: [Myelitis, Transverse] explode all trees
#5	MeSH descriptor: [Optic Neuritis] explode all trees
#6	("clinically isolated" NEXT syndrome*):ti,ab
#7	(devic OR "devic s" OR devics):ti,ab
#8	(disseminated NEXT sclerosis*):ti,ab
#9	(demyelinating NEXT (disease* OR disorder*)):ti,ab
#10	((demyelinating OR necrotising OR necrotizing OR transverse) NEXT myelitis*):ti,ab
#11	multiple sclerosis:ti,ab OR MS:ti
#12	(neuropapilliti* OR ((optic OR retrobulbar) NEXT neuriti*)):ti,ab
#13	((neuromyelitis NEXT optica*) OR ("nmo spectrum" NEXT disorder*)):ti,ab
#14	{OR #1‐#13}
#15	MeSH descriptor: [Adrenal Cortex Hormones] this term only and with qualifier(s): [therapeutic use ‐ TU, adverse effects ‐ AE]
#16	MeSH descriptor: [Alemtuzumab] explode all trees
#17	MeSH descriptor: [Azathioprine] explode all trees
#18	MeSH descriptor: [Cladribine] explode all trees
#19	MeSH descriptor: [Cyclophosphamide] explode all trees
#20	MeSH descriptor: [Daclizumab] explode all trees
#21	MeSH descriptor: [Dimethyl Fumarate] explode all trees
#22	MeSH descriptor: [Fingolimod Hydrochloride] explode all trees
#23	MeSH descriptor: [Glatiramer Acetate] explode all trees
#24	MeSH descriptor: [Immunoglobulins] this term only and with qualifier(s): [therapeutic use ‐ TU, adverse effects ‐ AE, drug effects ‐ DE]
#25	MeSH descriptor: [Immunoglobulins, Intravenous] explode all trees
#26	MeSH descriptor: [Interferon‐beta] explode all trees
#27	MeSH descriptor: [Interferon Type I] this term only
#28	MeSH descriptor: [Methotrexate] explode all trees
#29	MeSH descriptor: [Methylprednisolone] this term only
#30	MeSH descriptor: [Mitoxantrone] explode all trees
#31	MeSH descriptor: [Natalizumab] explode all trees
#32	MeSH descriptor: [Prednisolone] this term only
#33	MeSH descriptor: [Rituximab] explode all trees
#34	(("adrenal cortex" NEXT hormone*) OR corticoid*):ti,ab
#35	(corticosteroid* OR (cortico NEXT steroid*)):ti
#36	(alemtuzumab* OR campath* OR lemtrada*):ti,ab
#37	(avonex* OR rebif*):ti,ab
#38	(aubagio* OR teriflunomide*):ti,ab
#39	(azathioprine* OR azothioprine* OR imurel* OR imuran* OR immuran*):ti,ab
#40	(bafiertam* OR (monomethyl NEXT fumarate*) OR ("methyl hydrogen" NEXT fumarate*) OR methylhydrogenfumarate*):ti,ab
#41	((beta* NEAR/2 interferon*) OR fiblaferon* OR (fibroblast NEXT interferon*) OR IFNbeta* OR (IFN NEXT beta*)):ti,ab OR interferon*:ti
#42	(betaferon* OR betaseron* OR (beta NEXT seron*) OR extavia*):ti,ab
#43	(copaxone* OR "Cop 1" OR "copolymer 1" OR glatiramer* OR glatopa* OR "TV 5010" OR "TV5010"):ti,ab
#44	(cladribine* OR leustatin* OR mavenclad* OR movectro*):ti,ab
#45	(cyclophosphamide* OR cyclophosphane* OR cytophosphan* OR cytoxan* OR endoxan* OR neosar* OR procytox* OR sendoxan*):ti,ab
#46	(daclizumab* OR zinbryta* OR zenapax*):ti,ab
#47	(dimethylfumarate* OR (dimethyl NEXT fumarate*) OR "BG 00012" OR "BG00012" OR "BG12" OR (diroximel NEXT fumarate*) OR tecfidera* OR vumerity*):ti,ab
#48	(fingolimod* OR gilenya* OR gilenia* OR "FTY 720" OR "FTY720"):ti,ab
#49	(kesimpta* OR ofatumumab* OR "HUMAX CD20 2F2" OR "GSK 1841157" OR "GSK1841157"):ti,ab
#50	(immunoglobulin*):ti OR ((intravenous NEXT immunoglobulin*) OR (IV NEXT immunoglobulin*) OR IVIG):ti,ab
#51	(laquinimod* OR "ABR 215062" OR "ABR215062"):ti,ab
#52	(mayzent* OR siponimod* OR "BAF 312" OR "BAF312"):ti,ab
#53	(methotrexate* OR amethopterin* OR mexate*):ti,ab
#54	(methylprednisolone* OR metipred*):ti,ab
#55	(mitoxantrone* OR mitozantrone* OR ralenova* OR novantron* OR onkotrone*):ti,ab
#56	(natalizumab* OR tysabri* OR antegren*):ti,ab
#57	(ocrelizumab* OR ocrevus* OR "R 1594" OR "PR070769"):ti,ab
#58	(ozanimod* OR zeposia* OR "RPC1063"):ti,ab
#59	(peginterferon* OR (pegylated NEXT interferon*) OR plegridy* OR ("peg ifn" NEXT beta*)):ti,ab
#60	(prednisolone* OR predonine*):ti,ab
#61	(rituximab* OR rituxan* OR mabthera* OR "IDEC C2B8"):ti,ab
#62	{OR #15‐#61}
#63	#14 AND #62
#64	#14 AND #62 in Trials

### Appendix 2. MEDLINE (PubMed) search strategy

**Table:**

#	Query
1	("adverse effects" [Subheading]) AND "Multiple Sclerosis/drug therapy"[Majr]
2	"demyelinating autoimmune diseases, cns"[MeSH Terms:noexp]
3	"Demyelinating Diseases"[MeSH Terms:noexp]
4	"Multiple Sclerosis"[MeSH Terms]
5	"myelitis, transverse"[MeSH Terms]
6	"Optic Neuritis"[MeSH Terms]
7	"clinically isolated syndrome*"[Title/Abstract]
8	"devic"[Title/Abstract] OR "devic s"[Title/Abstract] OR "devics"[Title/Abstract]
9	"disseminated sclerosis*"[Title/Abstract]
10	"demyelinating disease*"[Title/Abstract] OR "demyelinating disorder*"[Title/Abstract]
11	"demyelinating myelitis*"[Title/Abstract] OR "necrotising myelitis*"[Title/Abstract] OR "necrotizing myelitis*"[Title/Abstract] OR "transverse myel*"[Title/Abstract]
12	"multiple sclerosis*"[Title/Abstract] OR "MS"[Title]
13	"neuropapilliti*"[Title/Abstract] OR "optic neuriti*"[Title/Abstract] OR "retrobulbar neuriti*"[Title/Abstract]
14	"neuromyelitis optica*"[Title/Abstract] OR "nmo spectrum disorder*"[Title/Abstract]
15	#2 OR #3 OR #4 OR #5 OR #6 OR #7 OR #8 OR #9 OR #10 OR #11 OR #12 OR #13 OR #14
16	( "Adrenal Cortex Hormones/adverse effects"[Mesh:NoExp] OR "Adrenal Cortex Hormones/drug effects"[Mesh:NoExp] OR "Adrenal Cortex Hormones/drug therapy"[Mesh:NoExp] OR "Adrenal Cortex Hormones/therapeutic use"[Mesh:NoExp] )
17	"Alemtuzumab"[MeSH Terms]
18	"Azathioprine"[MeSH Terms]
19	"Cladribine"[MeSH Terms]
20	"Cyclophosphamide"[MeSH Terms:noexp]
21	"Daclizumab"[MeSH Terms]
22	"Dimethyl Fumarate"[MeSH Terms]
23	"Fingolimod Hydrochloride"[MeSH Terms]
24	"Glatiramer Acetate"[MeSH Terms]
25	( "Immunoglobulins/adverse effects"[Mesh:NoExp] OR "Immunoglobulins/drug effects"[Mesh:NoExp] OR "Immunoglobulins/therapeutic use"[Mesh:NoExp] OR "Immunoglobulins, Intravenous"[MeSH Terms])
26	"Interferon‐beta"[MeSH Terms]
27	"Interferon Type I"[MeSH Terms:noexp]
28	"Methotrexate"[MeSH Terms]
29	"Methylprednisolone"[MeSH Terms:noexp]
30	"Mitoxantrone"[MeSH Terms]
31	"Natalizumab"[MeSH Terms]
32	"Prednisolone"[MeSH Terms:noexp]
33	"Rituximab"[MeSH Terms]
34	"adrenal cortex hormone*"[Title/Abstract] OR "corticosteroid*"[Title] OR "cortico steroid*"[Title] OR "corticoid*"[Title/Abstract]
35	"alemtuzumab*"[Title/Abstract] OR "campath*"[Title/Abstract] OR "lemtrada*"[Title/Abstract]
36	avonex*[Title/Abstract] OR rebif*[Title/Abstract]
37	"aubagio*"[Title/Abstract] OR "teriflunomide*"[Title/Abstract]
38	"azathioprine*"[Title/Abstract] OR "azothioprine*"[Title/Abstract] OR "imurel*"[Title/Abstract] OR "imuran*"[Title/Abstract] OR "immuran*"[Title/Abstract]
39	"bafiertam*"[Title/Abstract] OR "monomethyl fumarate*"[Title/Abstract] OR "methyl hydrogen fumarate*"[Title/Abstract] OR "methylhydrogenfumarate*"[Title/Abstract]
40	"beta interferon*"[Title/Abstract] OR "beta 1 interferon*"[Title/Abstract] OR "interferon beta*"[Title/Abstract] OR "fiblaferon*"[Title/Abstract] OR "fibroblast interferon*"[Title/Abstract] OR "IFNbeta*"[Title/Abstract] OR "IFN beta*"[Title/Abstract] OR "interferon*"[Title]
41	"betaferon*"[Title/Abstract] OR "betaseron*"[Title/Abstract] OR "beta seron*"[Title/Abstract] OR "extavia*"[Title/Abstract]
42	"copaxone*"[Title/Abstract] OR "Cop 1"[Title/Abstract] OR "copolymer 1"[Title/Abstract] OR "glatiramer*"[Title/Abstract] OR "glatopa*"[Title/Abstract] OR "TV 5010"[Title/Abstract] OR "TV5010"[Title/Abstract]
43	"cladribine*"[Title/Abstract] OR "leustatin*"[Title/Abstract] OR "mavenclad*"[Title/Abstract] OR "movectro*"[Title/Abstract]
44	"cyclophosphamide*"[Title/Abstract] OR "cyclophosphane*"[Title/Abstract] OR "cytophosphan*"[Title/Abstract] OR "cytoxan*"[Title/Abstract] OR "endoxan*"[Title/Abstract] OR "neosar*"[Title/Abstract] OR "procytox*"[Title/Abstract] OR "sendoxan*"[Title/Abstract]
45	"daclizumab*"[Title/Abstract] OR "zinbryta*"[Title/Abstract] OR "zenapax*"[Title/Abstract]
46	"dimethylfumarate"[Title/Abstract] OR "dimethyl fumarate*"[Title/Abstract] OR "BG 00012"[Title/Abstract] OR "BG00012"[Title/Abstract] OR "BG 12"[Title/Abstract] OR "diroximel fumarate*"[Title/Abstract] OR "tecfidera*"[Title/Abstract] OR "vumerity*"[Title/Abstract]
47	"fingolimod*"[Title/Abstract] OR "gilenya*"[Title/Abstract] OR "gilenia*"[Title/Abstract] OR "FTY 720"[Title/Abstract] OR "FTY720"[Title/Abstract]
48	"immunoglobulin*"[Title] OR "intravenous immunoglobulin*"[Title/Abstract] OR "IV immunoglobulin*"[Title/Abstract] OR "IVIG"[Title/Abstract]
49	"kesimpta*"[Title/Abstract] OR "ofatumumab*"[Title/Abstract] OR "HUMAX CD20 2F2"[Title/Abstract] OR "GSK 1841157"[Title/Abstract] OR "GSK1841157"[Title/Abstract]
50	"laquinimod*"[Title/Abstract] OR "ABR 215062"[Title/Abstract] OR "ABR215062"[Title/Abstract]
51	"mayzent*"[Title/Abstract] OR "siponimod*"[Title/Abstract] OR "BAF 312"[Title/Abstract] OR "BAF312"[Title/Abstract]
52	"methotrexate*"[Title/Abstract] OR "amethopterin*"[Title/Abstract] OR "mexate*"[Title/Abstract]
53	"methylprednisolone*"[Title/Abstract] OR "metipred*"[Title/Abstract]
54	"mitoxantrone*"[Title/Abstract] OR "mitozantrone*"[Title/Abstract] OR "ralenova*"[Title/Abstract] OR "novantron*"[Title/Abstract] OR "onkotrone*"[Title/Abstract]
55	"natalizumab*"[Title/Abstract] OR "tysabri*"[Title/Abstract] OR "antegren*"[Title/Abstract]
56	"ocrelizumab*"[Title/Abstract] OR "ocrevus*"[Title/Abstract] OR "R 1594"[Title/Abstract] OR "PR070769"[Title/Abstract]
57	"ozanimod*"[Title/Abstract] OR "zeposia*"[Title/Abstract] OR "RPC1063"[Title/Abstract]
58	"peginterferon*"[Title/Abstract] OR "pegylated interferon*"[Title/Abstract] OR "plegridy*"[Title/Abstract] OR "peg ifn beta*"[Title/Abstract]
59	"prednisolone*"[Title/Abstract] OR "predonine*"[Title/Abstract]
60	"rituximab*"[Title/Abstract] OR "rituxan*"[Title/Abstract] OR "mabthera*"[Title/Abstract] OR "IDEC C2B8"[Title/Abstract]
61	#16 OR #17 OR #18 OR #19 OR #20 OR #21 OR #22 OR #23 OR #24 OR #25 OR #26 OR #27 OR #28 OR #29 OR #30 OR #31 OR #32 OR #33 OR #34 OR #35 OR #36 OR #37 OR #38 OR #39 OR #40 OR #41 OR #42 OR #43 OR #44 OR #45 OR #46 OR #47 OR #48 OR #49 OR #50 OR #51 OR #52 OR #53 OR #54 OR #55 OR #56 OR #57 OR #58 OR #59 OR #60
62	#15 AND #61
63	#1 OR #62
64	randomized controlled trial [pt]
65	controlled clinical trial [pt]
66	randomized [tiab]
67	placebo [tiab]
68	"Clinical Trials as Topic"[Mesh:NoExp]
69	randomly [tiab]
70	trial [ti]
71	#64 OR #65 OR #66 OR #67 OR #68 OR #69 OR #70
72	animals [mh] NOT humans [mh]
73	#71 NOT #72
74	#63 AND #73

### Appendix 3. Embase search strategy

**Table:**

1	'demyelinating disease'/de
2	'multiple sclerosis'/de
3	'optic neuritis'/de
4	'transverse myelitis'/exp
5	'clinically isolated syndrome*':ab,ti
6	devic:ab,ti OR 'devic s':ab.ti OR devics:ab,it
7	'disseminated sclerosis*':ab,ti
8	(demyelinating NEAR/1 (disease* OR disorder*)):ab,ti
9	((demyelinating OR necrotising OR necrotizing OR transverse) NEAR/1 myelitis*):ab,ti
10	'multiple sclerosis*':ab,ti OR 'MS':ti
11	neuropapilliti*:ab,ti OR ((optic OR retrobulbar) NEAR/1 neuriti*"):ab,ti
12	'neuromyelitis optica*':ab,ti OR 'nmo spectrum disorder*':ab,ti
13	#1 OR #2 OR #3 OR #4 OR #5 OR #6 OR #7 OR #8 OR #9 OR #10 OR #11 OR #12
14	'alemtuzumab'/de
15	'azathioprine'/de
16	'beta interferon'/exp
17	'cladribine'/de
18	'corticosteroid'/de/ae OR 'corticosteroid'/de/dt
19	'cyclophosphamide'/de/ae OR 'cyclophosphamide'/de/dt
20	'daclizumab'/de
21	'dimethyl fumarate'/de
22	'fingolimod'/de
23	'glatiramer'/de
24	'immunoglobulin'/de/ae or 'immunoglobulin'/de/dt or 'immunoglobulin'/de/iv
25	'methotrexate'/de/ae or 'methotrexate'/de/dt
26	'methylprednisolone'/de
27	'mitoxantrone'/de
28	'natalizumab'/de
29	'prednisolone'/de
30	'rituximab'/de
31	'adrenal cortex hormone*':ab,ti OR 'corticosteroid*':ti OR 'cortico steroid*':ti OR 'corticoid*':ab,ti
32	'alemtuzumab*':ab,ti OR 'campath*':ab,ti OR 'lemtrada*':ab,ti
33	avonex*:ab,ti OR rebif*:ab,ti
34	'aubagio*':ab,ti OR 'teriflunomide*':ab,ti
35	'azathioprine*':ab,ti OR 'azothioprine*':ab,ti OR 'imurel*':ab,ti OR 'imuran*':ab,ti OR 'immuran*':ab,ti
36	'bafiertam*':ab,ti OR 'monomethyl fumarate*':ab,ti OR 'methyl hydrogen fumarate*':ab,ti OR 'methylhydrogenfumarate*':ab,ti
37	'beta interferon*':ab,ti OR 'beta 1 interferon*':ab,ti OR 'interferon beta*':ab,ti OR 'fiblaferon*':ab,ti OR 'fibroblast interferon*':ab,ti OR 'IFNbeta*':ab,ti OR 'IFN beta*':ab,ti OR 'interferon':ti
38	'betaferon*':ab,ti OR 'betaseron*':ab,ti OR 'beta seron*':ab,ti OR 'extavia*':ab,ti
39	'copaxone*':ab,ti OR 'Cop 1':ab,ti OR 'copolymer 1':ab,ti OR 'glatiramer*':ab,ti OR 'glatopa*':ab,ti OR 'TV 5010':ab,ti OR 'TV5010':ab,ti
40	'cladribine*':ab,ti OR 'leustatin*':ab,ti OR 'mavenclad*':ab,ti OR 'movectro*':ab,ti
41	'cyclophosphamide*':ab,ti OR 'cyclophosphane*':ab,ti OR 'cytophosphan*':ab,ti OR 'cytoxan*':ab,ti OR 'endoxan*':ab,ti OR 'neosar*':ab,ti OR 'procytox*':ab,ti OR 'sendoxan*':ab,ti
42	'daclizumab*':ab,ti OR 'zinbryta*':ab,ti OR 'zenapax*':ab,ti
43	'dimethylfumarate*':ab,ti OR 'dimethyl fumarate*':ab,ti OR 'BG 00012':ab,ti OR 'BG00012':ab,ti OR 'BG 12':ab,ti OR 'diroximel fumarate*':ab,ti OR 'tecfidera*':ab,ti OR 'vumerity*':ab,ti
44	'fingolimod*':ab,ti OR 'gilenya*':ab,ti OR 'gilenia*':ab,ti OR 'FTY 720':ab,ti OR 'FTY720':ab,ti
45	'immunoglobulin*':ti OR 'intravenous immunoglobulin*':ab,ti OR "IV immunoglobulin*":ab,ti OR "IVIG":ab,ti
46	'kesimpta*':ab,ti OR 'ofatumumab*':ab,ti OR 'HUMAX CD20 2F2':ab,ti OR 'GSK 1841157':ab,ti OR 'GSK1841157':ab,ti
47	'laquinimod*':ab,ti OR 'ABR 215062':ab,ti OR 'ABR215062':ab,ti
48	'mayzent*':ab,ti OR 'siponimod*':ab,ti OR 'BAF 312':ab,ti OR 'BAF312':ab,ti
49	'methotrexate*':ab,ti OR 'amethopterin*':ab,ti OR 'mexate*':ab,ti
50	'methylprednisolone*':ab,ti OR 'metipred*':ab,ti
51	'mitoxantrone*':ab,ti OR 'mitozantrone*':ab,ti OR 'ralenova*':ab,ti OR 'novantron*':ab,ti OR 'onkotrone*':ab,ti
52	'natalizumab*':ab,ti OR 'tysabri*':ab,ti OR 'antegren*':ab,ti
53	'ocrelizumab*':ab,ti OR 'ocrevus*':ab,ti OR 'R 1594':ab,ti OR 'PR070769':ab,ti
54	'ozanimod*':ab,ti OR 'zeposia*':ab,ti OR 'RPC1063':ab,ti
55	'peginterferon*':ab,ti OR 'pegylated interferon*':ab,ti OR 'plegridy*':ab,ti OR 'peg ifn beta*':ab,ti
56	'prednisolone*':ab,ti OR 'predonine*':ab,ti
57	'rituximab*':ab,ti OR 'rituxan*':ab,ti OR 'mabthera*':ab,ti OR 'IDEC C2B8':ab,ti
58	#14 OR #15 OR #16 OR #17 OR #18 OR #19 OR #20 OR #21 OR #22 OR #23 OR #24 OR #25 OR #26 OR #27 OR #28 OR #29 OR #30 OR #31 OR #32 OR #33 OR #34 OR #35 OR #36 OR #37 OR #38 OR #39 OR #40 OR #41 OR #42 OR #43 OR #44 OR #45 OR #46 OR #47 OR #48 OR #49 OR #50 OR #51 OR #52 OR #53 OR #54 OR #55 OR #56 OR #57
59	#13 AND #58
60	'randomized controlled trial'/de
61	'controlled clinical trial'/de
62	random*:ti,ab,tt
63	'randomization'/de
64	'intermethod comparison'/de
65	placebo:ti,ab,tt
66	(compare:ti,tt OR compared:ti,tt OR comparison:ti,tt)
67	((evaluated:ab OR evaluate:ab OR evaluating:ab OR assessed:ab OR assess:ab) AND (compare:ab OR compared:ab OR comparing:ab OR comparison:ab))
68	(open NEXT/1 label):ti,ab,tt
69	((double OR single OR doubly OR singly) NEXT/1 (blind OR blinded OR blindly)):ti,ab,tt
70	'double blind procedure'/de
71	(parallel NEXT/1 group*):ti,ab,tt
72	(crossover:ti,ab,tt OR 'cross over':ti,ab,tt)
73	((assign* OR match OR matched OR allocation) NEAR/6 (alternate OR group OR groups OR intervention OR interventions OR patient OR patients OR subject OR subjects OR participant OR participants)):ti,ab,tt
74	(assigned:ti,ab,tt OR allocated:ti,ab,tt)
75	(controlled NEAR/8 (study OR design OR trial)):ti,ab,tt
76	(volunteer:ti,ab,tt OR volunteers:ti,ab,tt)
77	'human experiment'/de
78	trial:ti,tt
79	#60 OR #61 OR #62 OR #63 OR #64 OR #65 OR #66 OR #67 OR #68 OR #69 OR #70 OR #71 OR #72 OR #73 OR #74 OR #75 OR #76 OR #77 OR #78
80	(((random* NEXT/1 sampl* NEAR/8 ('cross section*' OR questionnaire* OR survey OR surveys OR database or databases)):ti,ab,tt) NOT ('comparative study'/de OR 'controlled study'/de OR 'randomised controlled':ti,ab,tt OR 'randomized controlled':ti,ab,tt OR 'randomly assigned':ti,ab,tt))
81	('cross‐sectional study'/de NOT ('randomized controlled trial'/de OR 'controlled clinical study'/de OR 'controlled study'/de OR 'randomised controlled':ti,ab,tt OR 'randomized controlled':ti,ab,tt OR 'control group':ti,ab,tt OR 'control groups':ti,ab,tt))
82	('case control*':ti,ab,tt AND random*:ti,ab,tt NOT ('randomised controlled':ti,ab,tt OR 'randomized controlled':ti,ab,tt))
83	('systematic review':ti,tt NOT (trial:ti,tt OR study:ti,tt))
84	(nonrandom*:ti,ab,tt NOT random*:ti,ab,tt)
85	'random field*':ti,ab,tt
86	('random cluster' NEAR/4 sampl*):ti,ab,tt
87	(review:ab AND review:it) NOT trial:ti,tt
88	('we searched':ab AND (review:ti,tt OR review:it))
89	'update review':ab
90	(databases NEAR/5 searched):ab
91	((rat:ti,tt OR rats:ti,tt OR mouse:ti,tt OR mice:ti,tt OR swine:ti,tt OR porcine:ti,tt OR murine:ti,tt OR sheep:ti,tt OR lambs:ti,tt OR pigs:ti,tt OR piglets:ti,tt OR rabbit:ti,tt OR rabbits:ti,tt OR cat:ti,tt OR cats:ti,tt OR dog:ti,tt OR dogs:ti,tt OR cattle:ti,tt OR bovine:ti,tt OR monkey:ti,tt OR monkeys:ti,tt OR trout:ti,tt OR marmoset*:ti,tt) AND 'animal experiment'/de)
92	('animal experiment'/de NOT ('human experiment'/de OR 'human'/de))
93	#80 OR #81 OR #82 OR #83 OR #84 OR #85 OR #86 OR #87 OR #88 OR #89 OR #90 OR #91 OR #92
94	#77 NOT #93
95	#59 AND #94
95	([medline]/lim OR [pubmed‐not‐medline]/lim)
96	#95 NOT #96

### Appendix 4. State utility values and effect thresholds for the primary outcomes in progressive multiple sclerosis

**Table:**

**Primary outcomes**	**Reference or utility description**	**Utility**	**1‐Utility**	**T1 Absolute risk per 1000**	**95% CI Lower Absolute**	**95% CI Upper Absolute**	**T2 Absolute risk per 1000**	**95% CI Lower Absolute**	**95% CI Upper Absolute**	**T3 Absolute risk per 1000**	**95% CI Lower Absolute**	**95% CI Upper Absolute**	**T1 Absolute Complete**	**T2 Absolute Complete**	**T3 Absolute Complete**
Relapse of multiple sclerosis 12 and 24 months	Hawton 2016	0.62	0.38	33	19	48	66	47	84	128	99	156	33 (19 to 48)	66 (47 to 84)	128 (99 to 156)
Relapse of multiple sclerosis 36 months	None, assumed	0.62	0.38	41	23	59	81	58	103	157	122	191	41 (23 to 59)	81 (58 to 103)	157 (122 to 191)
Disability or dependency (EDSS = 6) 24 and 36 months	Chataway 2021	0.481	0.519	30	17	43	59	43	76	115	89	140	30 (17 to 43)	59 (43 to 76)	115 (89 to 140)
Serious adverse events	None, assumed	0.600	0.400	39	22	56	77	55	98	149	116	182	39 (22 to 56)	77 (55 to 98)	149 (116 to 182)
Discontinuation of treatment due to adverse events	None, assumed	0.850	0.150	104	59	149	205	147	262	397	309	485	104 (59 to 149)	205 (147 to 262)	397 (309 to 485)

**CI:** confidence interval; **EDSS:** Expanded Disability Status Scale

### Appendix 5. Netleague table: Mortality

**Table:**

**Siponimod**	0.34 (0.01,11.17)	2.03 (0.51,8.15)	4.08 (0.30,54.89)	6.23 (0.19,204.71)	3.67 (0.11,120.89)	4.65 (0.66,32.91)	3.16 (0.46,21.71)	0.67 (0.02,22.01)	0.58 (0.09,3.70)	1.47 (0.09,23.64)	1.02 (0.06,16.98)
2.95 (0.09,97.15)	**Rituximab**	5.99 (0.24,147.94)	12.03 (0.25,586.59)	18.38 (0.20,1709.16)	10.81 (0.12,1008.49)	13.72 (0.42,449.83)	9.31 (0.29,300.33)	1.96 (0.02,183.49)	1.70 (0.05,52.77)	4.34 (0.08,238.65)	2.99 (0.05,169.43)
0.49 (0.12,1.98)	0.17 (0.01,4.12)	**Placebo/no treatment**	2.01 (0.22,18.07)	3.07 (0.12,75.52)	1.81 (0.07,44.62)	2.29 (0.58,9.08)	1.55 (0.41,5.91)	0.33 (0.01,8.13)	0.28 (0.08,0.98)	0.72 (0.07,8.02)	0.50 (0.04,5.79)
0.25 (0.02,3.30)	0.08 (0.00,4.05)	0.50 (0.06,4.48)	**Ocrelizumab**	1.53 (0.03,74.29)	0.90 (0.02,43.86)	1.14 (0.09,15.24)	0.77 (0.06,10.12)	0.16 (0.00,7.98)	0.14 (0.01,1.76)	0.36 (0.01,9.36)	0.25 (0.01,6.69)
0.16 (0.00,5.27)	0.05 (0.00,5.06)	0.33 (0.01,8.02)	0.65 (0.01,31.83)	**Natalizumab**	0.59 (0.01,54.74)	0.75 (0.02,24.40)	0.51 (0.02,16.29)	0.11 (0.00,9.96)	0.09 (0.00,2.86)	0.24 (0.00,12.95)	0.16 (0.00,9.19)
0.27 (0.01,8.99)	0.09 (0.00,8.63)	0.55 (0.02,13.69)	1.11 (0.02,54.28)	1.70 (0.02,158.15)	**Laquinimod**	1.27 (0.04,41.63)	0.86 (0.03,27.79)	0.18 (0.00,16.98)	0.16 (0.01,4.88)	0.40 (0.01,22.08)	0.28 (0.00,15.68)
0.21 (0.03,1.52)	0.07 (0.00,2.39)	0.44 (0.11,1.73)	0.88 (0.07,11.71)	1.34 (0.04,43.76)	0.79 (0.02,25.84)	**Interferon beta‐1b**	0.68 (0.10,4.62)	0.14 (0.00,4.71)	0.12 (0.02,0.79)	0.32 (0.02,5.05)	0.22 (0.01,3.63)
0.32 (0.05,2.18)	0.11 (0.00,3.47)	0.64 (0.17,2.45)	1.29 (0.10,16.91)	1.97 (0.06,63.53)	1.16 (0.04,37.52)	1.47 (0.22,10.05)	**Interferon beta‐1a**	0.21 (0.01,6.83)	0.18 (0.03,1.13)	0.47 (0.03,7.30)	0.32 (0.02,5.24)
1.50 (0.05,49.70)	0.51 (0.01,47.65)	3.05 (0.12,75.71)	6.13 (0.13,299.97)	9.37 (0.10,873.61)	5.51 (0.06,515.47)	6.99 (0.21,230.13)	4.74 (0.15,153.65)	**Immunoglobulins**	0.87 (0.03,27.00)	2.21 (0.04,122.03)	1.53 (0.03,86.63)
1.74 (0.27,11.16)	0.59 (0.02,18.31)	3.53 (1.03,12.14)	7.09 (0.57,88.12)	10.83 (0.35,335.44)	6.37 (0.20,198.11)	8.08 (1.27,51.42)	5.48 (0.89,33.85)	1.16 (0.04,36.07)	**Glatiramer acetate**	2.55 (0.17,38.14)	1.76 (0.11,27.43)
0.68 (0.04,10.93)	0.23 (0.00,12.69)	1.38 (0.12,15.30)	2.77 (0.11,72.04)	4.24 (0.08,232.66)	2.49 (0.05,137.33)	3.17 (0.20,50.55)	2.15 (0.14,33.61)	0.45 (0.01,25.00)	0.39 (0.03,5.85)	**Fingolimod**	0.69 (0.02,21.39)
0.98 (0.06,16.46)	0.33 (0.01,18.89)	2.00 (0.17,23.18)	4.02 (0.15,107.88)	6.14 (0.11,346.21)	3.61 (0.06,204.36)	4.58 (0.28,76.13)	3.11 (0.19,50.64)	0.66 (0.01,37.19)	0.57 (0.04,8.81)	1.45 (0.05,44.83)	**Azathioprine**

### Appendix 6. Relative treatment ranking (SUCRA and mean rank)

**Relapses over 24 months**

**Table:**

Treatment	SUCRA	PrBest	MeanRank
placebo_notreatment	28.1	0.1	3.9
immunoglobulins	39.1	1.2	3.4
interferon_beta1a_Avonex_Rebif	77.8	30.5	1.9
methotrexate	31.3	12.4	3.7
rituximab	73.6	55.8	2.1

**Relapses over 36 months**

**Table:**

Treatment	SUCRA	PrBest	MeanRank
placebo_notreatment	20.3	0.0	3.4
azathioprine	95.5	91.2	1.1
interferon_beta1b_Betaferon	67.3	8.2	2.0
interferon_beta1a_Avonex_Rebif	16.8	0.6	3.5

**Disability over 24 months**

**Table:**

Treatment	SUCRA	PrBest	MeanRank
placebo_notreatment	18.3	0.0	7.5
glatiramer_acetate	49.3	3.8	5.1
immunoglobulins	35.4	1.0	6.2
interferon_beta1b_Betaferon	64.9	33.9	3.8
interferon_beta1a_Avonex_Rebif	47.1	5.6	5.2
methotrexate	68.7	31.0	3.5
natalizumab	49.1	5.4	5.1
rituximab	58.1	10.4	4.4
siponimod	59.0	8.8	4.3

**Disability over 36 months**

**Table:**

Treatment	SUCRA	PrBest	MeanRank
placebo_notreatment	30.6	0.4	3.8
azathioprine	80.8	66.5	1.8
interferon_beta1b_Betaferon	54.1	9.2	2.8
interferon_beta1a_Avonex_Rebif	21.1	2.9	4.2
ocrelizumab	63.4	21.0	2.5

**Serious adverse events**

**Table:**

Treatment	SUCRA	PrBest	MeanRank
placebo_notreatment	59.1	0.2	5.5
fingolimod	54.4	3.9	6.0
glatiramer_acetate	35.0	3.2	8.2
immunoglobulins	4.4	0.2	11.5
interferon_beta1b_Betaferon	60.0	5.4	5.4
interferon_beta1a_Avonex_Rebif	58.5	7.0	5.6
laquinimod	41.8	6.1	7.4
methotrexate	56.3	42.3	5.8
natalizumab	66.7	9.0	4.7
ocrelizumab	66.8	9.8	4.7
rituximab	54.4	11.7	6.0
siponimod	42.7	1.1	7.3

**Treatment discontinuation due to adverse events**

**Table:**

Treatment	SUCRA	PrBest	MeanRank
placebo_notreatment	90.8	29.0	2.0
azathioprine	20.3	6.3	9.8
fingolimod	47.3	0.0	6.8
glatiramer_acetate	24.4	0.2	9.3
immunoglobulins	56.5	1.3	5.8
interferon_beta1b_Betaferon	32.8	0.0	8.4
interferon_beta1a_Avonex_Rebif	34.4	0.0	8.2
laquinimod	30.6	3.0	8.6
natalizumab	87.3	34.4	2.4
ocrelizumab	78.3	22.0	3.4
rituximab	27.9	2.6	8.9
siponimod	69.4	1.2	4.4

**T1 over 24 months**

**Table:**

Treatment	SUCRA	PrBest	MeanRank
placebo_notreatment	5.1	0.1	2.9
interferon_beta1a_Avonex_Rebif	67.3	35.6	1.7
siponimod	77.6	64.3	1.4

**T2 over 24 months**

**Table:**

Treatment	SUCRA	PrBest	MeanRank
placebo_notreatment	36.2	12.8	2.3
interferon_beta1a_Avonex_Rebif	59.4	44.4	1.8
siponimod	54.4	42.7	1.9

**Quality of life (physical subscale)**

**Table:**

Treatment	SUCRA	PrBest	MeanRank
placebo_notreatment	70.2	48.4	1.6
interferon_beta1a_Avonex_Rebif	30.1	14.4	2.4
ocrelizumab	49.7	37.2	2.0

**Mortality**

**Table:**

Treatment	SUCRA	PrBest	MeanRank
placebo_notreatment	45.5	0.0	7.0
azathioprine	61.9	10.2	5.2
fingolimod	53.8	6.0	6.1
glatiramer_acetate	78.9	11.7	3.3
immunoglobulins	68.1	22.8	4.5
interferon_beta1b_Betaferon	23.4	0.0	9.4
interferon_beta1a_Avonex_Rebif	32.9	0.1	8.4
laquinimod	34.8	3.8	8.2
natalizumab	25.5	1.9	9.2
ocrelizumab	30.0	0.7	8.7
rituximab	79.3	39.3	3.3
siponimod	66.0	3.6	4.7

### Appendix 7. Heterogeneity results within the network analyses

**Table:**

	**Standard deviation heterogeneity**	**Tau^2^ heterogeneity**
Disability worsening at 24 months	0.18	0.032
Disability worsening at 36 months	0.17748017	0.031
Relapse at 24 months	1.16E‐09	0.000
Relapse at 36 months	1.54E‐12	0.000
Number of participants with any serious adverse events	0.2403426	0.058
Discontinuation due to adverse events	1.03E‐07	0.000
Mortality	2.78E‐10	0.000

### Appendix 8. Incoherence results within the network analyses

**Serious adverse events**

Loop specific approach

**Table:**

Loop	IF	seIF	z_value	p_value	CI_95	Loop_Heterog_tau2
placebo_notreatment‐glatiramer_acetate‐rituximab	0.275	2.085	0.132	0.895	(0.00,4.36)	0.000

Node‐splitting method

**Table:**

01 placebo; 03 glatiramer acetate; 11 rituximab								
Side	Direct		Indirect		Difference			tau
	Coef.	Std. Err.	Coef.	Std. Err.	Coef.	Std. Err.	P>	|z|
01 02	**.**	**.**	**.**	**.**	**.**	**.**	**.**	**.**
01 03	.4233663	.6328295	.0024651	2.142835	.4209012	2.234326	0.851	.416393
01 04	**.**	**.**	**.**	**.**	**.**	**.**	**.**	**.**
01 05	**.**	**.**	**.**	**.**	**.**	**.**	**.**	**.**
01 06	**.**	**.**	**.**	**.**	**.**	**.**	**.**	**.**
01 07	**.**	**.**	**.**	**.**	**.**	**.**	**.**	**.**
01 08	**.**	**.**	**.**	**.**	**.**	**.**	**.**	**.**
01 09	**.**	**.**	**.**	**.**	**.**	**.**	**.**	**.**
01 10	**.**	**.**	**.**	**.**	**.**	**.**	**.**	**.**
01 11	‐.0446027	.6093807	.3762873	2.14962	‐.42089	2.234325	0.851	.4163926
01 12	.	.	.	.	.	.	.	.
03 11	‐.0470675	2.054378	‐.4679702	.8785329	.4209027	2.234343	0.851	.4163951

Global test: 'design‐by‐treatment' approach

chi2( 1) = 0.04

Prob > chi2 = 0.8506

**Treatment discontinuation due to adverse events**

Loop specific approach

**Table:**

Loop	IF	seIF	z_value	p_value	CI_95	Loop_Heterog_tau2
placebo_notreatment‐glatiramer_acetate‐rituximab	0.135	1.598	0.085	0.932	(0.00,3.27)	0.000

Node‐splitting method

**Table:**

01 placebo; 04 glatiramer acetate; 11 rituximab								
Side	Direct		Indirect		Difference			tau
	Coef.	Std. Err.	Coef.	Std. Err.	Coef.	Std. Err.	P>	|z|
01 02	**.**	**.**	**.**	**.**	**.**	**.**	**.**	**.**
01 03	**.**	**.**	**.**	**.**	**.**	**.**	**.**	**.**
01 04	1.365989	.5368552	1.501096	1.504653	‐.1351071	1.597559	0.933	9.66e‐11
01 05	**.**	**.**	**.**	**.**	**.**	**.**	**.**	**.**
01 06	**.**	**.**	**.**	**.**	**.**	**.**	**.**	**.**
01 07	**.**	**.**	**.**	**.**	**.**	**.**	**.**	**.**
01 08	**.**	**.**	**.**	**.**	**.**	**.**	**.**	**.**
01 09	**.**	**.**	**.**	**.**	**.**	**.**	**.**	**.**
01 10	**.**	**.**	**.**	**.**	**.**	**.**	**.**	**.**
01 11	1.451426	1.102026	1.315939	1.156822	.1354864	1.597716	0.932	1.18e‐10
01 12	**.**	**.**	**.**	**.**	**.**	**.**	**.**	**.**
04 11	‐.0499352	1.024697	.0853703	1.225828	‐.1353054	1.597688	0.933	9.77e‐11

Global test: 'design‐by‐treatment' approach

chi2( 1) = 0.01

Prob > chi2 = 0.9324

### Appendix 9. Clinical trial registers search strategy

**World Health Organization (WHO) International Clinical Trials Registry Platform (ICTRP) (apps.who.int/trialsearch)**

Search terms: relapsing multiple sclerosis, filtered for "Phase 2" "Phase 3" trials.

**US National Institutes of Health clinical trial register (www.clinicaltrials.gov)**

Search term: "relapsing multiple sclerosis".

## Data and analyses

**Comparison 1:** Treatment efficacy (primary outcomes): pairwise comparisons

Outcome or subgroup title	No. of studies	No. of participants	Statistical method	Effect size
1.1 Relapse (12 months)	1		Risk Ratio (IV, Random, 95% CI)	Subtotals only
1.1.1 Immunoglobulins versus placebo/no treatment	1	318	Risk Ratio (IV, Random, 95% CI)	1.04 [0.76, 1.41]
1.2 Relapse (24 months)	6		Risk Ratio (IV, Random, 95% CI)	Subtotals only
1.2.1 Immunoglobulins versus placebo/no treatment	2	549	Risk Ratio (IV, Random, 95% CI)	0.96 [0.79, 1.16]
1.2.2 Interferon beta 1a (Avonex, Rebif) versus placebo/no treatment	1	436	Risk Ratio (IV, Random, 95% CI)	0.72 [0.54, 0.95]
1.2.3 Methotrexate versus placebo/no treatment	1	60	Risk Ratio (IV, Random, 95% CI)	1.12 [0.38, 3.28]
1.2.4 Rituximab versus placebo/no treatment	1	439	Risk Ratio (IV, Random, 95% CI)	0.60 [0.19, 1.95]
1.2.5 Steroids versus cyclophosphamide	1	138	Risk Ratio (IV, Random, 95% CI)	1.55 [0.96, 2.51]
1.3 Relapse (36 months)	4		Risk Ratio (IV, Random, 95% CI)	Subtotals only
1.3.1 Azathioprine versus placebo/no treatment	1	67	Risk Ratio (IV, Random, 95% CI)	0.54 [0.30, 0.99]
1.3.2 Interferon beta 1b (Betaferon) versus placebo/no treatment	2	1657	Risk Ratio (IV, Random, 95% CI)	0.82 [0.73, 0.93]
1.3.3 Interferon beta 1a (Avonex, Rebif) versus placebo/no treatment	1	371	Risk Ratio (IV, Random, 95% CI)	1.03 [0.79, 1.34]
1.4 Disability worsening (24 months)	11		Risk Ratio (IV, Random, 95% CI)	Subtotals only
1.4.1 Glatiramer acetate versus placebo/no treatment	2	1049	Risk Ratio (IV, Random, 95% CI)	0.87 [0.74, 1.01]
1.4.2 Immunoglobulins versus placebo/no treatment	2	549	Risk Ratio (IV, Random, 95% CI)	0.92 [0.65, 1.30]
1.4.3 Interferon beta1b (Betaferon) versus placebo/no treatment	1	73	Risk Ratio (IV, Random, 95% CI)	0.69 [0.32, 1.48]
1.4.4 Interferon Beta1a (Avonex, Rebif) versus placebo/no treatment	1	436	Risk Ratio (IV, Random, 95% CI)	0.85 [0.64, 1.12]
1.4.5 Methotrexate versus placebo/no treatement	1	60	Risk Ratio (IV, Random, 95% CI)	0.69 [0.38, 1.24]
1.4.6 Natalizumab versus placebo/no treatment	1	889	Risk Ratio (IV, Random, 95% CI)	0.83 [0.67, 1.04]
1.4.7 Siponimod versu placebo/no treatment	1	1651	Risk Ratio (IV, Random, 95% CI)	0.77 [0.64, 0.93]
1.4.8 Rituximab versus placebo / no treatment	1	439	Risk Ratio (IV, Random, 95% CI)	0.78 [0.59, 1.02]
1.4.9 Steroids versus cyclophosphamide	1	138	Risk Ratio (IV, Random, 95% CI)	1.76 [0.96, 3.23]
1.5 Disability worsening (36 months)	5		Risk Ratio (IV, Random, 95% CI)	Subtotals only
1.5.1 Azathioprine versus placebo/no treatment	1	67	Risk Ratio (IV, Random, 95% CI)	0.63 [0.30, 1.33]
1.5.2 Interferon Beta 1b (Betaferon) versus placebo/no treatment	2	1657	Risk Ratio (IV, Random, 95% CI)	0.90 [0.68, 1.18]
1.5.3 Interferon Beta 1a (Avonex, Rebif) versus placebo/no treatement	1	371	Risk Ratio (IV, Random, 95% CI)	1.10 [0.85, 1.42]
1.5.4 Ocrelizumab versus placebo/no treatment	1	732	Risk Ratio (IV, Random, 95% CI)	0.83 [0.67, 1.03]

**Comparison 2:** Treatment safety (primary outcomes): pairwise comparisons

Outcome or subgroup title	No. of studies	No. of participants	Statistical method	Effect size
2.1 Serious adverse events	14	7914	Odds Ratio (IV, Random, 95% CI)	0.97 [0.74, 1.26]
2.1.1 Fingolimod versus placebo/no treatment	1	823	Odds Ratio (IV, Random, 95% CI)	1.05 [0.76, 1.46]
2.1.2 Glatiramer acetate versus placebo/no treatment	1	943	Odds Ratio (IV, Random, 95% CI)	1.53 [0.60, 3.89]
2.1.3 Immunoglobulins versus placebo/no treatment	2	549	Odds Ratio (IV, Random, 95% CI)	7.11 [1.27, 39.83]
2.1.4 Interferon beta‐1b versus placebo/no treatment	1	939	Odds Ratio (IV, Random, 95% CI)	0.41 [0.29, 0.57]
2.1.5 Interferon beta‐1a versus placebo/no treatment	1	364	Odds Ratio (IV, Random, 95% CI)	0.99 [0.63, 1.58]
2.1.6 Laquinimod versus placebo/no treatment	1	373	Odds Ratio (IV, Random, 95% CI)	1.32 [0.49, 3.55]
2.1.7 Natalizumab versus placebo/no treatment	1	888	Odds Ratio (IV, Random, 95% CI)	0.90 [0.65, 1.24]
2.1.8 Ocrelizumab versus placebo/no treatment	1	725	Odds Ratio (IV, Random, 95% CI)	0.90 [0.62, 1.31]
2.1.9 Rituximab versus placebo/no treatment	2	466	Odds Ratio (IV, Random, 95% CI)	0.88 [0.29, 2.65]
2.1.10 Siponimod versus placebo/no treatment	1	1646	Odds Ratio (IV, Random, 95% CI)	1.22 [0.92, 1.61]
2.1.11 Methotrexate versus placebo/no treatment	1	60	Odds Ratio (IV, Random, 95% CI)	Not estimable
2.1.12 Steroids versus cyclophosphamide	1	138	Odds Ratio (IV, Random, 95% CI)	0.86 [0.38, 1.95]
2.2 Discontinuations due to adverse events	19	9818	Odds Ratio (IV, Random, 95% CI)	1.97 [1.43, 2.70]
2.2.1 Azathioprine versus placebo/no treatment	1	67	Odds Ratio (IV, Random, 95% CI)	8.42 [0.42, 169.73]
2.2.2 Fingolimod versus placebo / no treatment	1	823	Odds Ratio (IV, Random, 95% CI)	2.29 [1.46, 3.60]
2.2.3 Glatiramer acetate versus placebo / no treatment	1	943	Odds Ratio (IV, Random, 95% CI)	3.92 [1.37, 11.23]
2.2.4 Immunoglobulins versus placebo / no treatements	2	549	Odds Ratio (IV, Random, 95% CI)	1.95 [0.99, 3.84]
2.2.5 Interferon Beta 1b versus placebo / no treatment	2	1657	Odds Ratio (IV, Random, 95% CI)	2.98 [1.92, 4.61]
2.2.6 Interferon Beta 1a versus placebo/no treatment	4	1455	Odds Ratio (IV, Random, 95% CI)	2.93 [1.63, 5.25]
2.2.7 Laquinimod versus placebo/no treatment	1	373	Odds Ratio (IV, Random, 95% CI)	3.75 [0.83, 16.99]
2.2.8 Natalizumab versus placebo/no treatment	1	888	Odds Ratio (IV, Random, 95% CI)	1.02 [0.55, 1.90]
2.2.9 Ocrelizumab versus placebo/no treatment	1	725	Odds Ratio (IV, Random, 95% CI)	1.24 [0.54, 2.86]
2.2.10 Rituximab versus placebo / no treatment	2	470	Odds Ratio (IV, Random, 95% CI)	4.35 [0.50, 37.65]
2.2.11 Rituximab versus glatiramer acetate	1	84	Odds Ratio (IV, Random, 95% CI)	0.95 [0.13, 7.09]
2.2.12 Siponimod versus placebo / no treatment	1	1646	Odds Ratio (IV, Random, 95% CI)	1.53 [0.98, 2.38]
2.2.13 Steroids versus cyclophosphamide	1	138	Odds Ratio (IV, Random, 95% CI)	0.21 [0.07, 0.61]

**Comparison 3:** Treatment efficacy and safety (secondary outcomes): pairwise comparisons

Outcome or subgroup title	No. of studies	No. of participants	Statistical method	Effect size
3.1 New gadolinium‐enhancing positive T1‐weighted MRI lesions (12 months)	2		Risk Ratio (IV, Random, 95% CI)	Subtotals only
3.1.1 Interferon beta‐1a versus placebo/no treatment	1	436	Risk Ratio (IV, Random, 95% CI)	0.40 [0.26, 0.61]
3.1.2 Rituximab versus glatiramer acetate	1	84	Risk Ratio (IV, Random, 95% CI)	0.72 [0.17, 3.00]
3.2 New gadolinium‐enhancing positive T1‐weighted MRI lesions (24 months)	2		Risk Ratio (IV, Random, 95% CI)	Subtotals only
3.2.1 Interferon beta‐1a versus placebo/no treatment	1	436	Risk Ratio (IV, Random, 95% CI)	0.46 [0.29, 0.71]
3.2.2 Siponimod versus placebo/no treatment	1	1651	Risk Ratio (IV, Random, 95% CI)	0.32 [0.26, 0.40]
3.3 New gadolinium‐enhancing positive T1‐weighted MRI lesions (36 months)	1		Risk Ratio (IV, Random, 95% CI)	Subtotals only
3.3.1 Fingolimod versus placebo	1	823	Risk Ratio (IV, Random, 95% CI)	0.58 [0.41, 0.82]
3.4 New or enlarging T2‐weighted MRI lesions (12 months)	1		Risk Ratio (IV, Random, 95% CI)	Subtotals only
3.4.1 Interferon beta‐1a (Avonex, Rebif) versus placebo	1	436	Risk Ratio (IV, Random, 95% CI)	0.53 [0.39, 0.73]
3.5 New or enlarging T2‐weighted MRI lesions (24 months)	2		Risk Ratio (IV, Random, 95% CI)	Subtotals only
3.5.1 Interferon beta‐1a versus placebo/no treatment	1	436	Risk Ratio (IV, Random, 95% CI)	0.62 [0.49, 0.80]
3.5.2 Siponimod versus placebo / no treatment	1	1651	Risk Ratio (IV, Random, 95% CI)	0.68 [0.62, 0.75]
3.6 New or enlarging T2‐weighted MRI lesions (36 months)	1		Risk Ratio (IV, Random, 95% CI)	Subtotals only
3.6.1 Fingolimod versus placebo	1	823	Risk Ratio (IV, Random, 95% CI)	0.51 [0.39, 0.66]
3.7 QoL total (MSIS‐29)	1		Mean Difference (IV, Random, 95% CI)	Subtotals only
3.7.1 Natalizumab versus placebo	1	889	Mean Difference (IV, Random, 95% CI)	2.73 [0.05, 5.41]
3.8 QoL Mental (SF‐36)	1		Mean Difference (IV, Random, 95% CI)	Subtotals only
3.8.1 Interferon beta‐1a (Avonex, Rebif) versus placebo	1	436	Mean Difference (IV, Random, 95% CI)	1.99 [0.22, 3.76]
3.9 QoL physical (SF‐36)	2		Std. Mean Difference (IV, Random, 95% CI)	Subtotals only
3.9.1 Interferon beta‐1a versus placebo/no treatment	1	436	Std. Mean Difference (IV, Random, 95% CI)	0.10 [‐0.09, 0.28]
3.9.2 Ocrelizumab versu placebo	1	732	Std. Mean Difference (IV, Random, 95% CI)	0.04 [‐0.12, 0.19]
3.10 Mortality	15		Odds Ratio (IV, Random, 95% CI)	Subtotals only
3.10.1 Azathioprine versus placebo/no treatment	1	67	Odds Ratio (IV, Random, 95% CI)	0.50 [0.04, 5.79]
3.10.2 Fingolimod versus placebo/no treatment	1	823	Odds Ratio (IV, Random, 95% CI)	0.72 [0.07, 8.02]
3.10.3 Glatiramer acetate versus placebo/no treatment	1	943	Odds Ratio (IV, Random, 95% CI)	0.28 [0.08, 0.98]
3.10.4 Immunoglobulins versus placebo/no treatment	1	230	Odds Ratio (IV, Random, 95% CI)	0.33 [0.01, 8.20]
3.10.5 Interferon beta‐1b versus placebo/no treatment	3	1731	Odds Ratio (IV, Random, 95% CI)	2.28 [0.58, 9.03]
3.10.6 Interferon beta‐1a versus placebo/no treatment	3	1425	Odds Ratio (IV, Random, 95% CI)	1.55 [0.41, 5.91]
3.10.7 Laquinimod versus placebo/no treatment	1	374	Odds Ratio (IV, Random, 95% CI)	1.81 [0.07, 44.62]
3.10.8 Natalizumab versus placebo/no treatment	1	889	Odds Ratio (IV, Random, 95% CI)	3.07 [0.12, 75.52]
3.10.9 Ocrelizumab versus placebo/no treatment	1	732	Odds Ratio (IV, Random, 95% CI)	2.01 [0.22, 18.07]
3.10.10 Rituximab versus placebo/no treatment	1	441	Odds Ratio (IV, Random, 95% CI)	0.25 [0.02, 2.78]
3.10.11 Siponimod versus placebo/no treatment	1	1651	Odds Ratio (IV, Random, 95% CI)	0.49 [0.12, 1.98]

## Characteristics of studies

### Characteristics of included studies [ordered by study ID]

**Table:** Anderson 2004

***Study characteristics***		
Methods	RCT	
Participants	Individuals with PMS, number randomised: 371. Prior use of DMT not reported. Age, years, mean (SD): placebo = 46.4 (NR); interferon beta‐1a = 45.1 (NR). Female sex: placebo = 107; interferon beta‐1a = 112	
Interventions	Interferon beta‐1a, 0.022 mg, once weekly, number randomised: 188 Placebo, once weekly, number randomised: 183 Treatment duration: 36 months Follow‐up: 36 months	
Outcomes	Mortality Relapses at 36 months Disability at 36 months SAEs Treatment discontinuation due to AEs	
Notes	Industry funding: Serono International, Geneva, Switzerland	
***Risk of bias***		
**Bias**	**Authors' judgement**	**Support for judgement**
Random sequence generation (selection bias)	Unclear risk	no information provided
Allocation concealment (selection bias)	Unclear risk	no information provided
Blinding of participants and personnel (performance bias) All outcomes	Unclear risk	only stated as double blind
Blinding of outcome assessment (detection bias) All outcomes	Low risk	Neurologists blinded to dose assignment were responsible for neurological assessments
Incomplete outcome data (attrition bias) All outcomes	Low risk	ITT analysis
Selective reporting (reporting bias)	Unclear risk	No protocol available
Other bias	Unclear risk	No information provided about role of funding intsitution

**Table:** ARPEGGIO 2020

***Study characteristics***		
Methods	RCT	
Participants	Individuals with PMS, number randomised: 374. Prior use of DMT at any time prior to the start of study: 24% (20% in laquinimod 0.6 mg, 32% in laquinimod 1.5 mg, and 22% in placebo). Age, years, mean (SD): placebo = 46.6 (7.2); laquinimod = 46.1 (7). Female sex, n (%): placebo = 67 (47.8); laquinimod = 102 (43.6)	
Interventions	Laquinimod, 1.5 mg + 0.6 mg, once daily, number randomised: 234 Placebo, once daily, number randomised: 140 Treatment duration: 12 months Follow‐up: 12 months	
Outcomes	Mortality Treatment discontinuation due to AEs SAEs	
Notes	Funding: Teva Pharmaceutical Industries	
***Risk of bias***		
**Bias**	**Authors' judgement**	**Support for judgement**
Random sequence generation (selection bias)	Unclear risk	no information provided
Allocation concealment (selection bias)	Low risk	quote: "Randomization was performed centrally using an independent interactive Web‐based or voice response system"
Blinding of participants and personnel (performance bias) All outcomes	Low risk	quote: " Patients, investigators, the sponsor, and designated personnel were blinded to treatment assignments".
Blinding of outcome assessment (detection bias) All outcomes	Low risk	quote: "EDSS and FSS assessments were performed by an examining neurologist who remained unaware of the patient’s safety status and was strictly instructed not to discuss safety issues with the treating physician, to assure an accurate and objective evaluation."
Incomplete outcome data (attrition bias) All outcomes	Low risk	ITT analysis
Selective reporting (reporting bias)	Low risk	all the outcomes listed in the protocol were reported in the final publication
Other bias	High risk	Industry funded. Quote: " Thomas Li performed the statistical analysis and is affiliated with Teva Pharmaceutical Industries Ltd". All the other authors are employees of Teva Pharm.

**Table:** ASCEND 2018

***Study characteristics***		
Methods	RCT	
Participants	Individuals with PMS, number randomised: 889. Prior use of DMT not reported. Age, years, mean (SD): placebo = 47.2 (7.8); natalizumab = 47.3 (7.4). Female sex, n (%): placebo = 280 (63); natalizumab = 270 (62)	
Interventions	Natalizumab, 300 mg every 4 weeks, number randomised: 440 Placebo, every 4 weeks, number randomised: 449 Treatment duration: 24 months Length of follow‐up: 24 months	
Outcomes	Mortality Disability at 24 months SAEs QoL Treatment discontinuation due to AEs	
Notes	Funding: Biogen	
***Risk of bias***		
**Bias**	**Authors' judgement**	**Support for judgement**
Random sequence generation (selection bias)	Unclear risk	no information provided
Allocation concealment (selection bias)	Low risk	quote. "randomly assigned by an interactive voice/web response system"
Blinding of participants and personnel (performance bias) All outcomes	Low risk	quote: "Only the pharmacists preparing the infusion and the pharmacy study monitors were not masked to the study treatment, which was stored in a secure location and accounted for by the investigator."
Blinding of outcome assessment (detection bias) All outcomes	Low risk	quote: "Only the pharmacists preparing the infusion and the pharmacy study monitors were not masked to the study treatment, which was stored in a secure location and accounted for by the investigator."
Incomplete outcome data (attrition bias) All outcomes	Low risk	ITT analysis
Selective reporting (reporting bias)	Low risk	all the outcomes listed in the protocol were reported in the final publication
Other bias	High risk	Private funding. All the authors were employees or received personal compensation by BIOGEN who sponsored the study. Quote: "The funder was also involved in study design, data collection, data analysis, and data interpretation, and reviewed and provided feedback on this manuscript.

**Table:** Bornstein 1991

***Study characteristics***		
Methods	RCT	
Participants	Individuals with PMS, number randomised: 106. Prior use of DMT not reported. Age, years, mean (SD): placebo = 41.6 (NR); glatiramer acetate = 41.6 (NR). Female sex, n (%): placebo = 30 (54.5); glatiramer acetate = 23 (45.5)	
Interventions	Glatiramer acetate 30 mg subcutaneous twice a day, number randomised: 51 Placebo, saline alone, subcutaneous twice a day, number randomised: 55	
Outcomes	Mortality Disability at 24 months	
Notes		
***Risk of bias***		
**Bias**	**Authors' judgement**	**Support for judgement**
Random sequence generation (selection bias)	Low risk	quote: allocation was " randomized block design with two baseline EDSS strata, t 5. 0 and 5.0 or greater"
Allocation concealment (selection bias)	Low risk	Quote: "When a patient became eligible, the investigator notified the statistical center, which validated the patient's eligibility and assigned a randomization code number. Only the statistician and the clinical assistant at Albert Einstein College of Medicine, who distributed medication, were aware of patient assignments."
Blinding of participants and personnel (performance bias) All outcomes	Unclear risk	only stated as double blind
Blinding of outcome assessment (detection bias) All outcomes	Low risk	quote: "The blinded neurologist performed a complete neurologic examination at each study visit. Another blinded neurologist was available to examine patients with severe or unusual side effects"
Incomplete outcome data (attrition bias) All outcomes	Low risk	ITT analysis
Selective reporting (reporting bias)	Unclear risk	no protocol available
Other bias	Low risk	Public funding

**Table:** Cheshmavar 2020

***Study characteristics***		
Methods	RCT	
Participants	Individuals with PMS, number randomised: 84. Prior use of DMT not reported. Age, years, mean (SD): rituximab = 40.95 (8.3); glatiramer acetate = 44.85 (7.95). Female sex, n (%): rituximab = 34 (79.1); glatiramer acetate = 26 (63.4)	
Interventions	Rituximab, 1000 mg, 3 courses of intravenous infusion of 1000 mg each 6 months apart, number randomised: 43 Glatiramer acetate, 40 mg, 3 times per week, number randomised: 41 Treatment duration: 12 months Follow‐up: 12 months	
Outcomes	Mortality SAEs Treatment discontinuation due to AEs Lesions on T1‐weighted MRI	
Notes	Public funding: Isfahan University of Medical Sciences	
***Risk of bias***		
**Bias**	**Authors' judgement**	**Support for judgement**
Random sequence generation (selection bias)	Unclear risk	no information provided
Allocation concealment (selection bias)	Unclear risk	no information provided
Blinding of participants and personnel (performance bias) All outcomes	High risk	Open label
Blinding of outcome assessment (detection bias) All outcomes	High risk	Open label
Incomplete outcome data (attrition bias) All outcomes	Low risk	ITT analysis
Selective reporting (reporting bias)	High risk	primary outcomes changed after study completion
Other bias	Low risk	public funding

**Table:** Ellison 1989

***Study characteristics***		
Methods	RCT	
Participants	Individuals with PMS, number randomised: 67. Prior use of DMT not reported. Age, years, mean (SD): placebo = 33.4 (9.5); azathioprine = 30.7 (10.5). Female sex, n (%): placebo = 15 (44); azathioprine = 17 (51)	
Interventions	Azathioprine 3 mg/kg body weight oral in 4 doses daily for 36 weeks (n = 33) Placebo in 4 oral doses daily (n = 34) Treatment duration: 36 months Length of follow‐up: 36 months	
Outcomes	Mortality Relapse at 36 months Disability at 36 months Treatment discontinuation due to AEs	
Notes	The study was funded by Wellcome Company, and Upjohn Company supplied the methylprednisolone and placebo.	
***Risk of bias***		
**Bias**	**Authors' judgement**	**Support for judgement**
Random sequence generation (selection bias)	Low risk	Block randomization process. Quote: " a master list was computed in which treatment was assigned according to the patinet sequence number".
Allocation concealment (selection bias)	Low risk	Quote: " the statistician told the examining neurologist tha the tretatment would be allocacted by a randomization process to block of four successive patients,, but the assigmne trules were not revealed".
Blinding of participants and personnel (performance bias) All outcomes	Low risk	Placebo controlled study. Quote "both observer and monitor neurologist, the study nurse, the clinic coordinator anf the patients were masked to the treatment assigned".
Blinding of outcome assessment (detection bias) All outcomes	Low risk	Placebo controlled study. Quote "both observer and monitor neurologist, the study nurse, the clinic coordinator anf the patients were masked to the treatment assigned".
Incomplete outcome data (attrition bias) All outcomes	Low risk	16% and 18% lost at follow‐up; balanced between groups; reasons provided.
Selective reporting (reporting bias)	Unclear risk	no protocol available
Other bias	Low risk	The study was funded by Wellcome Company and Upjohn Company. Burroughs Wellcome Co. provided azathioprine and appropriate placebos. The Upjohn Co. supplied the methylprednisolone and placebos" (Page 1025).

**Table:** Etemadifar 2019

***Study characteristics***		
Methods	RCT	
Participants	Individuals with PMS, number randomised: 80. Prior use of DMT not reported. Age, years, mean (SD): rituximab = 31.9 (7.7); cyclophosphamide = 37.9 (7.5). Female sex, n (%): rituximab = 35 (27); cyclophosphamide = 22 (73)	
Interventions	Rituximab, 1000 mg on days 1 and 15 every 6 months, number randomised: 40 Cyclophosphamide, 1000 mg every month, number randomised: 40 Treatment duration: 24 months Follow‐up: 24 months	
Outcomes	At 12 months: Disability measured by the EDSS Relapse New T2 lesion on MRI Gadolinium‐enhancing lesions on MRI Number of participants with AEs	
Notes	Funding: NR	
***Risk of bias***		
**Bias**	**Authors' judgement**	**Support for judgement**
Random sequence generation (selection bias)	Low risk	random allocation software
Allocation concealment (selection bias)	Low risk	each patient was given a number in a concealed envelope
Blinding of participants and personnel (performance bias) All outcomes	High risk	open label
Blinding of outcome assessment (detection bias) All outcomes	Low risk	Quote: "The second neurologist who evaluated patients during each visit was blinded to the consumed drug and recorded his examination assessment in a previously designed checklist. The second blinded neurologist was the same for all patients and checked possible medication side effects during each session regardless of the consumed drug. "
Incomplete outcome data (attrition bias) All outcomes	High risk	25% drop out from the endoxon group; 2.5% for the RTX groups, unbalaced; hgh drop out rate from one group; unbalanced
Selective reporting (reporting bias)	Unclear risk	no protocol available
Other bias	Unclear risk	no information provided about funding

**Table:** European Study Group 1998

***Study characteristics***		
Methods	RCT	
Participants	Individuals with PMS, number randomised: 718. Prior use of DMT not reported. Age, years, mean (SD): placebo = 40.9 (7.2); interferon beta‐1b = 41.1 (7.2). Female sex, n (%): placebo = 229 (64.2); interferon beta‐1b = 209 (58.1)	
Interventions	Interferon beta‐1b (Betaferon), 0.25 mg, 3 doses per week, number randomised: 360 Placebo, 3 doses per week, number randomised: 358 Treatment duration: 36 months Follow‐up: 33 months	
Outcomes	Mortality Relapse at 36 months Disability at 36 months Treatment discontinuation due to AEs	
Notes	Funding: Schering AG, Berlin	
***Risk of bias***		
**Bias**	**Authors' judgement**	**Support for judgement**
Random sequence generation (selection bias)	Low risk	block randomization process
Allocation concealment (selection bias)	Low risk	quote: "central randomisation schedule assigned placebo or interferon ‐1b to blocks of six patients in a 1/1 ratio. Access to the code was strictly limited according to study protocol"
Blinding of participants and personnel (performance bias) All outcomes	Low risk	double blind; quote: " Interferon ‐1b was indistinguishable from placebo"
Blinding of outcome assessment (detection bias) All outcomes	Low risk	quote: "To avoid unmasking as a result of the well‐characterised sideeffects of interferon ‐1b,1,2 designated treating physicians were responsible only for general medical care, safety assessments, and treatment of relapses, while designated EDSS physicians did the standardised neurological tests. EDSS physicians received no potentially unmasking information from the treating physicians, and were allowed to speak to patients only as necessary to carry out neurological tests."
Incomplete outcome data (attrition bias) All outcomes	Low risk	9 % lost at follow‐up for each group: balanced
Selective reporting (reporting bias)	Unclear risk	no protocol available
Other bias	Unclear risk	private funding. Quote: "The study was supervised by a steering committee of investigators and sponsor staff who were masked from the results throughout the study"

**Table:** EXPAND 2018

***Study characteristics***		
Methods	RCT	
Participants	Individuals with PMS, number randomised: 1651. Prior use of DMT: 21.7% (22% in siponimod, and 21% in placebo groups). Age, years, mean (SD): placebo = 48.1 (7.9); siponimod = 48 (7.8). Female sex, n (%): placebo = 323 (59); siponimod = 669 (61)	
Interventions	Siponimod, 2 mg daily, number randomised: 1105 Placebo, once daily, number randomised: 546 Treatment duration: 24 months Follow‐up: 24 months	
Outcomes	Mortality Disability at 24 months Treatment discontinuation due to AEs MRI outcomes Cognitive decline	
Notes	Funding: Novartis Pharma AG	
***Risk of bias***		
**Bias**	**Authors' judgement**	**Support for judgement**
Random sequence generation (selection bias)	Low risk	Quote: "blocked randomisation with a block size of 6. Randomisation was stratified for each of the 31 countries.
Allocation concealment (selection bias)	Low risk	quote: "randomisation list was produced by an interactive response technology provider (Parexel, Billerica, MA, USA) using a validated system automating the random assignment of patient numbers to randomisation numbers."
Blinding of participants and personnel (performance bias) All outcomes	Low risk	quote: "Study drug and placebo were identical in packaging, labelling, schedule of administration, appearance, taste, and odour" "Patients and study staff remained masked to treatment assignment for the duration of the core part of the study"
Blinding of outcome assessment (detection bias) All outcomes	Low risk	quote: "Patients and study staff remained masked to treatment assignment for the duration of the core part of the study" "All EDSS scores were obtained by trained, certified assessors who were not otherwise involved in patient management"
Incomplete outcome data (attrition bias) All outcomes	Low risk	ITT analysis
Selective reporting (reporting bias)	Low risk	all the outcomes listed in the protocol were reported in the final publication
Other bias	High risk	private funder. Quote: "The funder participated in the study design and conduct, data collection, management, analysis and interpretation, and the writing of the study report"

**Table:** Goodkin 1995

***Study characteristics***		
Methods	RCT	
Participants	Individuals with PMS, number randomised: 60. Prior use of DMT not reported. Age, years, mean (SD): placebo = 46 (8.8); methotrexate = 43 (9.3). Female sex, n (%): placebo = 15 (51.7); methotrexate = 20 (64.4)	
Interventions	Methotrexate, 7.5 mg once a week, number randomised: 31 Placebo, once a week, number randomised: 29 Treatment duration: 24 months Follow‐up: 24 months	
Outcomes	Mortality Relapse at 24 months Disability at 24 months Treatment discontinuation due to AEs	
Notes	Funding: National Multiple Sclerosis Society	
***Risk of bias***		
**Bias**	**Authors' judgement**	**Support for judgement**
Random sequence generation (selection bias)	Low risk	quote: "randomization scheme was developed for each strata prior to the initiation of the study and was blocked in groups of 10"
Allocation concealment (selection bias)	High risk	quote: "Treatment assignments were made by the unblinded study coordinator (M.M.D.) once the eligibility of the patient was confirmed"
Blinding of participants and personnel (performance bias) All outcomes	Low risk	double blind; quote: " visually indistinguishable PLC tablets"
Blinding of outcome assessment (detection bias) All outcomes	Low risk	quote:"blinded examining neurologist"
Incomplete outcome data (attrition bias) All outcomes	Low risk	ITT analysis
Selective reporting (reporting bias)	Unclear risk	no protocol available
Other bias	Low risk	public funding

**Table:** Hawker 2009

***Study characteristics***		
Methods	RCT	
Participants	Individuals with PMS, number randomised: 439. Use of DMT at any time prior to the start of study: 35%. Age, years, mean (SD): placebo = 49.6 (8.7); rituximab = 50.1 (9). Female sex, n (%): placebo = 81 (55.1); rituximab = 140 (47.9)	
Interventions	Rituximab, 1000 mg on days 1 and 15, 4 courses in 24 h, number randomised: 292 Placebo, 4 courses in 24 h, number randomised: 147 Treatment duration: 22 months Follow‐up: 24 months	
Outcomes	Mortality Relapse at 24 months Disability at 24 months SAEs Treatment discontinuation due to AEs	
Notes	Private funding: Genentech	
***Risk of bias***		
**Bias**	**Authors' judgement**	**Support for judgement**
Random sequence generation (selection bias)	Unclear risk	no information provided
Allocation concealment (selection bias)	Unclear risk	no information provided
Blinding of participants and personnel (performance bias) All outcomes	Unclear risk	only stated as double blind
Blinding of outcome assessment (detection bias) All outcomes	Unclear risk	only stated as double blind
Incomplete outcome data (attrition bias) All outcomes	Low risk	ITT analysis
Selective reporting (reporting bias)	Low risk	protocol available; all outcomes reported in the publication
Other bias	High risk	Private funding

**Table:** Hommes 2004

***Study characteristics***		
Methods	RCT	
Participants	Individuals with PMS, number randomised: 318. Prior use of DMT not reported. Age, years, mean (SD): placebo = 43.4 (6.8); immunoglobulins = 44 (7.2). Female sex, n (%): placebo = 93 (58.5); immunoglobulins = 100 (62.9)	
Interventions	Immunoglobulins, 70,000 mg per month, number randomised: 159 Placebo, monthly, number randomised: 159 Treatment duration: 27 months Follow‐up: 27 months	
Outcomes	Mortality Relapse at 12 and 24 months Disability at 24 months Treatment discontinuations due to AEs	
Notes	Funding: Bayer Corporation	
***Risk of bias***		
**Bias**	**Authors' judgement**	**Support for judgement**
Random sequence generation (selection bias)	Low risk	quote: "randomisation was done centrally as block randomisation with stratification by centre"
Allocation concealment (selection bias)	Unclear risk	no information provided
Blinding of participants and personnel (performance bias) All outcomes	Low risk	quote:"Concealment of treatment was guaranteed by use of an albumin solution identical in appearance to the study medication, with identical labelling and opaque plastic wrapping"; "Physicians and study nurses were unaware of treatment allocation."
Blinding of outcome assessment (detection bias) All outcomes	Low risk	quote: "To maintain masking, a treating neurologist or a study nurse administered the study drug, and the clinical assessment of the patients was made by an evaluating neurologist, who was not allowed to discuss therapy or potential adverse effects with the patients. Physicians and study nurses were unaware of treatment allocation."
Incomplete outcome data (attrition bias) All outcomes	Low risk	ITT analysis
Selective reporting (reporting bias)	Low risk	authors reported and justified changes of primary outcomes form the 1st version of the protocol
Other bias	Low risk	Private funding: "the study ws conducted under the control of the Independent Data and Safety Monitoring Board. The analysis of the data followed the statistical analysis plan and was supervised by the Steering Committee in collaboration with the sponsor."

**Table:** IMPACT 2002

***Study characteristics***		
Methods	RCT	
Participants	Individuals with PMS, number randomised: 436. Prior use of DMT not reported. Age, years, mean (SD): placebo = 47.9 (7.7); interferon beta‐1a (Avonex, Rebif) = 47.2 (8.2). Female sex, n (%): placebo = 141 (64); interferon beta‐1a (Avonex, Rebif) = 138 (64)	
Interventions	Interferon beta‐1a (Avonex, Rebif), 0.06 mg, 1 dose per week, number randomised: 217 Placebo, 1 dose per week, number randomised: 219 Treatment duration: 24 months Follow‐up: 24 months	
Outcomes	Mortality Relapse at 24 months Disability at 24 months SAEs Quality of life (SF‐36) MRI measures (T1, T2 lesions) Treatment discontinuation due to AEs	
Notes	Funding: Biogen Inc	
***Risk of bias***		
**Bias**	**Authors' judgement**	**Support for judgement**
Random sequence generation (selection bias)	Low risk	quote: "A minimization procedure was used to balance the treatment groups. The contract research organization computer generated two minimization schemes, one for North America and one for Europe and Israel."
Allocation concealment (selection bias)	Unclear risk	no information provided
Blinding of participants and personnel (performance bias) All outcomes	Unclear risk	only stated as double blind
Blinding of outcome assessment (detection bias) All outcomes	Low risk	quote: " Neither the examining technician nor the examining neurologist was involved with any other aspect of subject care, and neither had access to the results of prior examinations or to clinical information that might compromise blinding."
Incomplete outcome data (attrition bias) All outcomes	Low risk	ITT analysis
Selective reporting (reporting bias)	Unclear risk	no protocol available
Other bias	High risk	private funding. Four study authors are fully employees of Pharmaceutical compamy funding the study.

**Table:** INFORMS 2016

***Study characteristics***		
Methods	RCT	
Participants	Individuals with PMS, number randomised: 823. Prior use of DMT not reported. Age, years, mean (SD): placebo = 48.5 (8.3); fingolimod = 48.5 (8.6). Female sex, n (%): placebo = 235 (48); fingolimod = 163 (49)	
Interventions	Fingolimod, 0.5 mg daily, number randomised: 336 Placebo, once daily, number randomised: 487 Treatment duration: 60 months Follow‐up: 60 months	
Outcomes	Mortality Treatment discontinuation due to AEs SAEs MRI outcomes	
Notes	Funding: Novartis Pharma	
***Risk of bias***		
**Bias**	**Authors' judgement**	**Support for judgement**
Random sequence generation (selection bias)	Low risk	quote: "computer "generated blocks to receive either fi ngolimod or placebo. The randomisation sequence was automatically generated"
Allocation concealment (selection bias)	Low risk	quote: "Allocation was concealed through the use of blinded code‐break cards with removable, scratch‐off cover for the whole double‐blind treatment period"
Blinding of participants and personnel (performance bias) All outcomes	Low risk	quote: " We achieved masking by use of identical packaging and identical capsule colour and size for treatment and placebo". "All randomised drug assignments remained masked to patients, investigator staff, people performing the assessments, and data analysts for the whole double‐blind treatment period."
Blinding of outcome assessment (detection bias) All outcomes	Low risk	quote: " "All randomised drug assignments remained masked to patients, investigator staff, people performing the assessments, and data analysts for the whole double‐blind treatment period." "Employees of the funder who were independent of the study team monitored first dose safety and were masked to study allocation."
Incomplete outcome data (attrition bias) All outcomes	Low risk	ITT analysis
Selective reporting (reporting bias)	Low risk	all the outcomes listed in the protocol were reported in the final publication
Other bias	High risk	Industry funded. The funder participated in the design and conduct of the study; data collection, management, analysis, and interpretation; and preparation, review, and approval of the paper

**Table:** Komori 2016

***Study characteristics***		
Methods	RCT	
Participants	Individuals with PMS, number randomised: 27. Prior use of DMT not reported. Age, years, median (range): placebo = 60.1 (39.3 to 64.8); rituximab = 55.2 (42.0 to 66.0). Female sex, n (%): placebo = 7 (77.8); rituximab = 7 (50)	
Interventions	Rituximab, intrathecal injection of 25 mg (1:1 dilution in normal saline) and intravenous infusion of 200 mg at month zero, followed by an additional 200 mg intravenously at month 0.5 and another 25 mg intrathecal at months 1.5 and 12, number randomised: 18 Placebo, 3 courses, number randomised: 9 Treatment duration: 12 months Follow‐up: 24 months	
Outcomes	Mortality SAEs Treatment discontinuation due to AEs	
Notes	Funding: National Institutes of Health (NIH), Bethesda, Maryland, USA	
***Risk of bias***		
**Bias**	**Authors' judgement**	**Support for judgement**
Random sequence generation (selection bias)	Low risk	Quote: "Randomization was done by the NIH pharmacy using a table of random numbers". Block randomization
Allocation concealment (selection bias)	Unclear risk	no information
Blinding of participants and personnel (performance bias) All outcomes	Unclear risk	only stated as double blind
Blinding of outcome assessment (detection bias) All outcomes	Unclear risk	only stated as double blind
Incomplete outcome data (attrition bias) All outcomes	High risk	22% drop out for the intervention group only; unbalanced
Selective reporting (reporting bias)	Low risk	protocol available; all outcomes reported in the publication
Other bias	Low risk	public funding

**Table:** Leary 2003

***Study characteristics***		
Methods	RCT	
Participants	Individuals with PMS, number randomised: 50. Prior use of DMT not reported. Age, years, median (range): placebo = 43 (30 to 59); interferon beta‐1a = 46.7 (25 to 59). Female sex, n (%): placebo = 5 (25); interferon beta‐1a = 13 (43.33)	
Interventions	Interferon beta‐1a (Avonex, Rebif), 0.03 mg + 0.06 mg, 1 dose per week, number randomised: 30 Placebo, 1 dose per week, number randomised: 20	
Outcomes	Mortality SAEs Treatment discontinuation due to AEs	
Notes	Funding: Biogen	
***Risk of bias***		
**Bias**	**Authors' judgement**	**Support for judgement**
Random sequence generation (selection bias)	Low risk	quote:"randomized by the block method"
Allocation concealment (selection bias)	Unclear risk	no information provided
Blinding of participants and personnel (performance bias) All outcomes	Low risk	stated as double blind: quote: "Subjects and study personnel were blinded to treatment status. EDSS assessments were performed by an independent evaluating physician blinded to all clinical information"
Blinding of outcome assessment (detection bias) All outcomes	Low risk	quote:"EDSS assessments were performed by an independent evaluating physician blinded to all clinical information"
Incomplete outcome data (attrition bias) All outcomes	Low risk	ITT analysis
Selective reporting (reporting bias)	Unclear risk	no protocol available
Other bias	Unclear risk	Private funding. No information about the role of sponsor

**Table:** Montalban 2009

***Study characteristics***		
Methods	RCT	
Participants	Individuals with PMS, number randomised: 73. All participants were previously untreated. Age, years, mean (SD): placebo = 48.6 (8.7); interferon beta‐1b = 48.8 (7.5). Female sex, n (%): placebo = 22 (60); interferon beta‐1b = 14 (39)	
Interventions	Interferon beta‐1b, 0.25 mg, 3 doses per week, number randomised: 36 Placebo, 3 doses per week, number randomised: 37 Treatment duration: 24 months Follow‐up: 24 months	
Outcomes	Disability at 24 months Treatment discontinuation due to AEs	
Notes	Funding: Schering Espana S.A.	
***Risk of bias***		
**Bias**	**Authors' judgement**	**Support for judgement**
Random sequence generation (selection bias)	Low risk	quote:"randomized (using a randomization list) into blocks of six"
Allocation concealment (selection bias)	Unclear risk	no information provided
Blinding of participants and personnel (performance bias) All outcomes	Unclear risk	only stated as double blind
Blinding of outcome assessment (detection bias) All outcomes	Unclear risk	no information provided
Incomplete outcome data (attrition bias) All outcomes	Low risk	ITT analysis
Selective reporting (reporting bias)	Unclear risk	no protocol available
Other bias	High risk	quote"The study sponsor (SCHERING ESPAN˜ A S.A), participated in the study design, monitored data collection and approved the final version and the decision to submit the paper for publication"

**Table:** NASP 2004

***Study characteristics***		
Methods	RCT	
Participants	Individuals with PMS, number randomised: 939. Prior use of DMT not reported. Age, years, mean (SD): placebo = 47.6 (0.46); interferon beta‐1b = 46.45 (0.45). Female sex, n (%): placebo = 185 (60); interferon beta‐1b = 403 (66.8)	
Interventions	Interferon beta‐1b, 0.25 mg, 3 doses per week, number randomised: 631 Placebo, 3 doses per week, number randomised: 308 Treatment duration: 36 months Follow‐up: 36 months	
Outcomes	Mortality Relapse at 36 months Disability at 36 months SAEs Treatment discontinuation due to AEs Quality of life: MSQOL‐54	
Notes	Funding: Berlex Laboratories (Richmond, CA, USA)	
***Risk of bias***		
**Bias**	**Authors' judgement**	**Support for judgement**
Random sequence generation (selection bias)	Low risk	quote: "Randomization allocation was by blocks of six"
Allocation concealment (selection bias)	Unclear risk	no information provided
Blinding of participants and personnel (performance bias) All outcomes	Unclear risk	only stated as double blind
Blinding of outcome assessment (detection bias) All outcomes	Low risk	quote: "Examining physicians were responsible for completing standardized neurologic evaluations and were not permitted access to previous examination results or any other information that could potentially unblind them to treatment assignment"
Incomplete outcome data (attrition bias) All outcomes	Low risk	ITT analysis
Selective reporting (reporting bias)	Unclear risk	no protocol available
Other bias	Unclear risk	Private funding: no information provided on the role of funder

**Table:** ORATORIO 2017

***Study characteristics***		
Methods	RCT	
Participants	Individuals with PMS, number randomised: 732. Use of DMT in the 2 years prior to the start of study: 11.6% (11.3% in ocrelizumab and 12.3% in placebo groups). Age, years, mean (SD): placebo = 44.4 (8.3); ocrelizumab = 44.7 (7.9). Female sex, n (%): placebo = 124 (50.8); ocrelizumab = 237 (48.6)	
Interventions	Ocrelizumab, 600 mg total via dual infusion of 300 mg every 24 weeks, number randomised: 488 Placebo, 600 mg total via dual infusion of 300 mg every 24 weeks, number randomised: 244 Treatment duration: 30 months Follow‐up: 30 months	
Outcomes	Mortality Disability at 36 months Treatment discontinuation due to AEs SAEs QoL	
Notes	Funding: Hoffmann‐La Roche	
***Risk of bias***		
**Bias**	**Authors' judgement**	**Support for judgement**
Random sequence generation (selection bias)	Low risk	quote. "Randomization was stratified according to geographic region and age" was performed centrally by an independent interactive Web‐response system"
Allocation concealment (selection bias)	Low risk	quote: " Randomization was performed centrally by an independent interactive Web‐response system"
Blinding of participants and personnel (performance bias) All outcomes	Unclear risk	only stated as double blind
Blinding of outcome assessment (detection bias) All outcomes	Low risk	quote: "An independent, trained investigator who was unaware of the trial‐group assignments and was certified in administering the EDSS conducted the neurologic examination and scored the EDSS.". "MRI scans were analyzed independently at a central MRI reading center by staff members who were unaware of the trial group assignments"
Incomplete outcome data (attrition bias) All outcomes	Low risk	ITT analysis
Selective reporting (reporting bias)	Unclear risk	no protocol available
Other bias	High risk	Industry funded. quote: " the sponsor desgined the trial; data were collected by the investigators and analyzed by the sponsor; the results were reviewed by the sponsor and steering committee"

**Table:** Pohlau 2007

***Study characteristics***		
Methods	RCT	
Participants	Individuals with PMS, number randomised: 231. Prior use of DMT not reported. Age, years, mean (SD): placebo = 48.1 (9.3); immunoglobulins = 47.8 (9.6). Female sex, n (%): placebo = 68 (59); immunoglobulins = 68 (59)	
Interventions	Immunoglobulins, 28,000 mg monthly, number randomised: 116 Placebo, number randomised: 116 Treatment duration: 24 months Follow‐up: 24 months	
Outcomes	Relapse at 24 months DIsability at 24 months SAEs Treatment discontinuation due to AEs	
Notes	Funding: Novartis Pharma GmbH and ZLB Behring	
***Risk of bias***		
**Bias**	**Authors' judgement**	**Support for judgement**
Random sequence generation (selection bias)	Low risk	quote: "randomization scheme provided balanced blocks of patient numbers for both treatment groups and the two diagnostic layers"
Allocation concealment (selection bias)	Unclear risk	no information provided
Blinding of participants and personnel (performance bias) All outcomes	Low risk	quote: "placebo could not visually be distinguished."
Blinding of outcome assessment (detection bias) All outcomes	Unclear risk	no information provided
Incomplete outcome data (attrition bias) All outcomes	Low risk	ITT analysis
Selective reporting (reporting bias)	Low risk	all the outcomes listed in the protocol were reported in the final publication
Other bias	Unclear risk	private funding: no information provided on the role of funder

**Table:** PROMESS 2017

***Study characteristics***		
Methods	RCT	
Participants	Individuals with PMS, number randomised: 138. Prior use of DMT not reported. Age, years, mean (SD): placebo = 46.8 (9); cyclophosphamide = 48.6 (9). Female sex, n (%): placebo = 45 (62); cyclophosphamide = 67 (47.8)	
Interventions	Cyclophosphamide, 750 mg/m^2^, once every 4 weeks during the first 12 months and every 8 weeks during the second 12 months, number randomised: 72 Steroids, 1000 mg, once every 4 weeks during the first 12 months and every 8 weeks during the second 12 months, number randomised: 66 Treatment duration: 24 months Follow‐up: 24 months	
Outcomes	Relapse at 24 months Disability at 24 months Treatment discontinuation due to AEs SAEs	
Notes	Funding: French Ministry of Health (Programme Hospitalier de Recherche Clinique 2004)	
***Risk of bias***		
**Bias**	**Authors' judgement**	**Support for judgement**
Random sequence generation (selection bias)	Low risk	quote: "Randomization, with a 1:1 ratio, was stratified by centre, with blocks of size four for small sites and size six for others"
Allocation concealment (selection bias)	Low risk	quote. "web‐based secured system according to a randomization list generated and kept confidential by the statistician of the Clinical Trials Unit"
Blinding of participants and personnel (performance bias) All outcomes	Low risk	quote. "Study drugs were prepared in hospital pharmacies in similar infusion vials that precluded the identification of the group assignment by patients and study personnel"
Blinding of outcome assessment (detection bias) All outcomes	Low risk	quote: "Because the study drugs have potential adverse effects that may make patients and clinicians guess the treatment received, outcome assessments were performed by an evaluating neurologist (EN);"
Incomplete outcome data (attrition bias) All outcomes	Low risk	ITT analysis. High rate of drop out. Quote: "In the analyses of secondary endpoints, missing data due to early discontinuation of treatment because of adverse events were considered treatment failures as were progressions."
Selective reporting (reporting bias)	High risk	some outcomes declared in the protcol not reported in the publication
Other bias	Low risk	public funder

**Table:** SPECTRIMS 2001

***Study characteristics***		
Methods	RCT	
Participants	Individuals with PMS, number randomised: 618. Prior use of DMT not reported. Age, years, mean (SD): placebo = 42.7 (6.8); interferon beta‐1a = 42.85 (7.3). Female sex, n (%): placebo = 123 (60); interferon beta‐1a = 267 (64.6)	
Interventions	Interferon beta‐1a, 0.022 mg + 0.044 mg, 3 doses per week, number randomised: 413 Placebo, 3 doses per week, number randomised: 205 Treatment duration: 36 months Follow‐up: 36 months	
Outcomes	Mortality Treatment discontinuation due to AEs	
Notes	Funding: Serono International, Geneva, Switzerland	
***Risk of bias***		
**Bias**	**Authors' judgement**	**Support for judgement**
Random sequence generation (selection bias)	Low risk	quote: computer generated randomization list provided by Serono, stratified by center"
Allocation concealment (selection bias)	Low risk	quote: "Treatment assignments were provided to investigators in sealed envelopes. The manufacturer labeled containers of study medication with patient identification numbers based on the randomization list, and patients received the medication labeled with their numbers"
Blinding of participants and personnel (performance bias) All outcomes	Low risk	quote: "Solutions of IFNb‐1a and placebo were physically indistinguishable, and packaging and labeling were prepared to preserve blinding"
Blinding of outcome assessment (detection bias) All outcomes	Low risk	quote: "Because IFN side effects are well recognized, a treating physician supervised drug administration, monitored safety, and managed adverse events, and a separate evaluating physician conducted neurologic assessments and followed‐up exacerbations"
Incomplete outcome data (attrition bias) All outcomes	Low risk	ITT analysis
Selective reporting (reporting bias)	Unclear risk	no protocol available
Other bias	High risk	Private funding, Serono performed statistical analyses

**Table:** Wolinsky 2007

***Study characteristics***		
Methods	RCT	
Participants	Individuals with PMS, number randomised: 943. Prior use of DMT not reported. Age, years, mean (SD): placebo = 50.2 (8.1); glatiramer acetate = 50.4 (8.4). Female sex, n (%): placebo = 152 (48.1); glatiramer acetate = 331 (52.8)	
Interventions	Glatiramer acetate, 20 mg, 7 doses/week, number randomised: 627 Placebo, 7 doses/week, number randomised: 316 Treatment duration: 36 months Follow‐up: 36 months	
Outcomes	Mortality Disability at 24 months SAEs Treatment discontinuation due to AEs	
Notes	Funding: Teva Pharmaceutical	
***Risk of bias***		
**Bias**	**Authors' judgement**	**Support for judgement**
Random sequence generation (selection bias)	Unclear risk	no information provided
Allocation concealment (selection bias)	Unclear risk	no information provided
Blinding of participants and personnel (performance bias) All outcomes	Unclear risk	no information provided
Blinding of outcome assessment (detection bias) All outcomes	Low risk	quote: " examining neurologist who were blinded to treatment."
Incomplete outcome data (attrition bias) All outcomes	Low risk	ITT analysis
Selective reporting (reporting bias)	Unclear risk	no protocol available
Other bias	Unclear risk	Industry funded: no information privided on the the role of sponsor

AE, adverse events; DMT, disease‐modifying treatment; EDSS, Expanded Disability Status Scale; MRI, magnetic resonance imaging; MSQOL‐54, Multiple Sclerosis Quality of Life‐54; NR, not reported; PMS, progressive multiple sclerosis; QoL, quality of life; RCT, randomised controlled trial; SAE, serious adverse events; SD, standard deviation; SF‐36, 36‐Item Short Form Health Survey

### Characteristics of excluded studies [ordered by study ID]

**Table:**

Study	Reason for exclusion
British and Dutch 1988	Mixed sample with < 80% of people with progressive forms of MS
CCMSSG 1991	Treatment duration 22 weeks
Edan 1997	Treatment duration 6 months
Evdoshenko 2019	Subanalysis of registrative RCT
Fox 2018	Exploratory subanalysis, outcome not of interest
Ghezzi 1989	Wrong publication type: abstract
Hartung 2002	Mixed sample with < 80% of people with progressive forms of MS
Kuhle 2016	Open‐label extension of EU Study Group RCT
Milanese 1993	Mixed sample with < 80% of people with progressive forms of MS
Miller 1961	Phenotype (relapsing/progressive multiple sclerosis) unclear
Wolinsky 2018	Post hoc analysis, outcome not of interest

MS, multiple sclerosis; RCT, randomised controlled trial

### Characteristics of ongoing studies [ordered by study ID]

**Table:** EUCTR2012‐003056‐36

Study name	Study of efficacy, safety and tolerability data for BAF312 compared to placebo in patients with secondary progressive multiple sclerosis
Methods	Multicentre, randomised, double‐blind, parallel‐group, placebo‐controlled variable treatment duration study
Participants	Individuals with secondary progressive multiple sclerosis
Interventions	BAF312 (siponimod)
Outcomes	BAF312 (siponimod) Placebo
Starting date	31 October 2012
Contact information	www.clinicaltrialsregister.eu/ctr-search/search?query=eudract_number:2012-003056-36
Notes

**Table:** EUCTR2014‐003021‐18‐PL

Study name	A safety and efficacy study of BG00012 in slowing the progression of disability in patients with secondary progressive multiple sclerosis
Methods	Controlled trial
Participants	Individuals with secondary progressive multiple sclerosis
Interventions	Dimethyl fumarate Placebo
Outcomes	Safety and efficacy
Starting date	17 May 2015
Contact information	trialsearch.who.int/Trial2.aspx?TrialID=EUCTR2014%E2%80%90003021%E2%80%9018%E2%80%90PL
Notes

**Table:** EUCTR2018‐001511‐73‐ES

Study name	A study to evaluate the efficacy and safety of ocrelizumab in adults with primary progressive multiple sclerosis
Methods	RCT
Participants	Adults with primary progressive multiple sclerosis
Interventions	Ocrelizumab Placebo
Outcomes	Efficacy and safety
Starting date	21 August 2019
Contact information	trialsearch.who.int/Trial2.aspx?TrialID=EUCTR2018%E2%80%90001511%E2%80%9073%E2%80%90ES
Notes

**Table:** EUCTR2018‐001511‐73‐GB

Study name	A phase IIIb multicenter, randomized, double‐blind, placebo‐controlled study to evaluate the efficacy and safety of ocrelizumab in adults with primary progressive multiple sclerosis
Methods	RCT
Participants	Primary progressive multiple sclerosis
Interventions	Ocrelizumab Placebo
Outcomes	Efficacy and safety
Starting date	23 December 2019
Contact information	www.clinicaltrialsregister.eu/ctr-search/search?query=eudract_number:2018-001511-73
Notes

**Table:** EUCTR2018‐005038‐39‐GB

Study name	A phase 2b study of cladribine to halt deterioration in people with advanced multiple sclerosis
Methods	RCT
Participants	Individuals with advanced multiple sclerosis
Interventions	Cladribine Placebo
Outcomes	Disability, limb function
Starting date	2 December 2020
Contact information	trialsearch.who.int/Trial2.aspx?TrialID=EUCTR2018%E2%80%90005038%E2%80%9039%E2%80%90GB
Notes

**Table:** EUCTR2020‐002981‐15‐DK

Study name	Non‐inferiority study of ocrelizumab and rituximab in active multiple sclerosis
Methods	RCT
Participants	Individuals with active multiple sclerosis
Interventions	Ocrelizumab and rituximab
Outcomes	Relapse Disability MRI outcomes
Starting date	15 December 2020
Contact information	trialsearch.who.int/Trial2.aspx?TrialID=EUCTR2020%E2%80%90002981%E2%80%9015%E2%80%90DK
Notes

**Table:** IRCT20130812014333N125

Study name	Comparison of effectiveness and complication of rituximab and fingolimod in improvement disability motion
Methods	Controlled trial
Participants	Individuals with multiple sclerosis
Interventions	Rituximab and fingolimod
Outcomes	Disability
Starting date	2018‐05‐10
Contact information	trialsearch.who.int/Trial2.aspx?TrialID=IRCT20130812014333N125
Notes

**Table:** NCT04035005

Study name	A study to evaluate the efficacy and safety of ocrelizumab in adults with primary progressive multiple sclerosis
Methods	RCT
Participants	Primary progressive multiple sclerosis
Interventions	Ocrelizumab Placebo
Outcomes	Efficacy and safety
Starting date	12 August 2019
Contact information	clinicaltrials.gov/ct2/show/NCT04035005
Notes

**Table:** NCT04688788

Study name	Non‐inferiority Study of Ocrelizumab and Rituximab in Active Multiple Sclerosis (DanNORMS)
Methods	RCT
Participants	Active multiple sclerosis
Interventions	Ocrelizumab and rituximab
Outcomes	MRI and clinical outcomes
Starting date	28 April 2021
Contact information	clinicaltrials.gov/ct2/show/NCT04688788
Notes

**Table:** NCT04695080

Study name	ChariotMS ‐ Cladribine to Halt Deterioration in People With Advanced Multiple Sclerosis (ChariotMS)
Methods	RCT
Participants	Individuals with advanced multiple sclerosis
Interventions	Cladribine Placebo
Outcomes	Disability
Starting date	
Contact information	clinicaltrials.gov/study/NCT04695080
Notes	

MRI, magnetic resonance imaging; RCT, randomised controlled trial

## Differences between protocol and review

We added further detail to the Why it is important to do this review section specifying the data in the current review serving as an evidence base for a multistakeholder panel appointed by the Multiple Sclerosis International Federation (MSIF) (MSIF Essential Medicines Panel (MEMP)).

We updated the Objectives to specify the individual immunomodulators and immunosuppressants.

We moved the outcome 'treatment discontinuation due to adverse events' from secondary to primary outcomes.

We added further detail to the Summary of findings and assessment of the certainty of the evidence section explaining the fully contextualised approach and the health state utility values used to inform it.

Insufficient information precluded our planned subgroup analyses. Namely, all studies included people with active progressive multiple sclerosis, and the definition of previous lack of response to treatment varied among studies.

We were unable to perform our planned sensitivity analyses because insufficient numbers of studies met the definitions specified in Sensitivity analysis.
